# Underlying Framework of All-optical Controlled Synaptic Devices for Neuromorphic Computing

**DOI:** 10.1007/s40820-026-02171-2

**Published:** 2026-04-07

**Authors:** Dunan Hu, Ruqi Yang, Zhizhen Ye, Jianguo Lu

**Affiliations:** State Key Laboratory of Silicon and Advanced Semiconductor Materials, School of Materials Science and Engineering, Zhejiang University, Hangzhou, 310058 People’s Republic of China

**Keywords:** All-optical control, Artificial synapse, Device mechanisms, Design framework, Neuromorphic computing

## Abstract

The rapid expansion of artificial intelligence has led to significant challenges in energy consumption and computational efficiency. To address these issues, the exploration and development of all-optical controlled (AOC) synaptic devices represents a promising leap forward in neuromorphic computing, offering potential solutions to the inherent limitations of traditional von Neumann architectures. AOC synaptic devices, utilizing exclusively optical signals to emulate bidirectional modulation of synaptic weights, bypass the complexity and additional energy costs associated with conventional electrical or electro-optical hybrid signals. This review articulates the underlying framework and fundamental motivations for studying AOC synapses, while systematically reviewing current research progress. We particularly highlight the synergistic relationships among physical mechanisms, material behaviors, and device architectures, as well as neuromorphic computing based on optical writing and optical erasing of information. By systematically interpreting these multidimensional correlations, we propose scalable and reproducible strategies for device design. This work will certainly herald a substantial direction of AOC synapses, providing an ideal platform for exploring neuromorphic computing for artificial intelligence.

## Highlights

- Neuromorphic computing, using artificial synaptic devices, mimics the structure and function of biological neural networks, offering a solution to the von Neumann bottleneck for the next-generation of artificial intelligence.
- All-optical controlled synapses enable bidirectional modulation of conductivity through light pulses for both optical writing and optical erasing of information, crucial for hardware breakthroughs in neuromorphic computing.
- A scalable and replicable design framework for all-optical controlled synaptic devices could play a pivotal role in advancing neuromorphic computing for a new era of artificial intelligence.

## Graphical abstract

**Figure:**
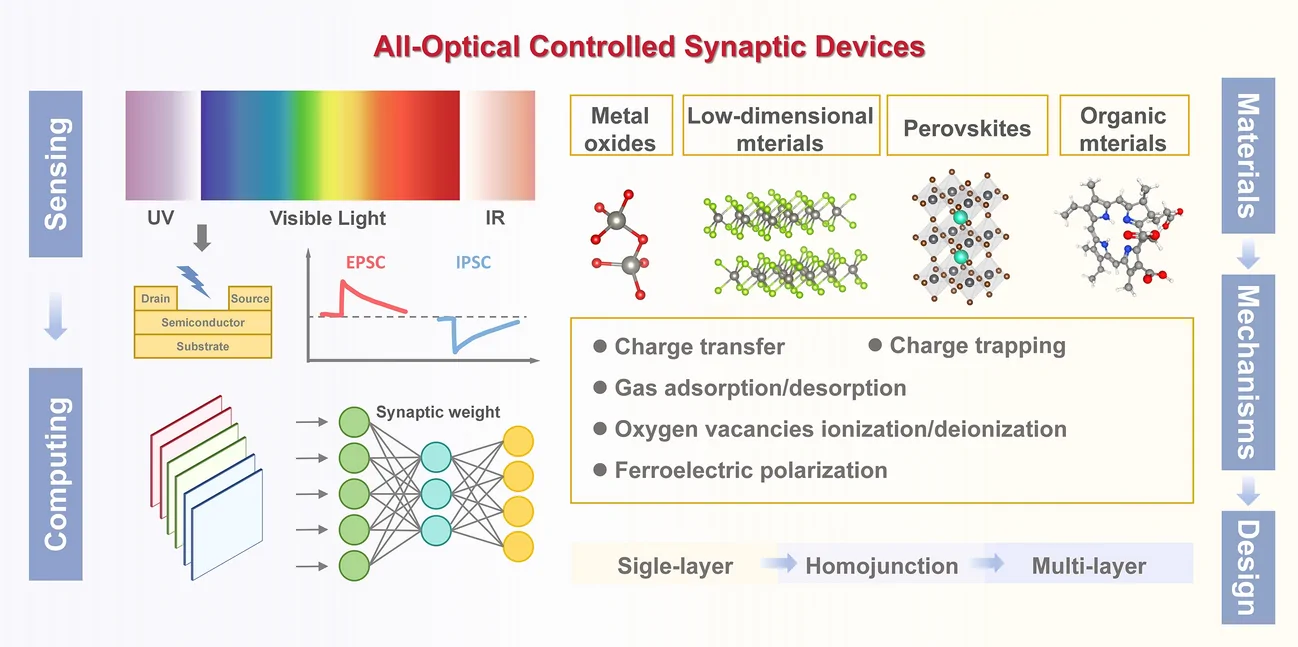

## Introduction

Driven by breakthroughs in deep learning and large-scale models like GPT-4, artificial intelligence (AI) has transformed fields such as natural language processing and autonomous systems [1–3]. The emergence of DeepSeek in 2025 has once again ignited the hot topic of artificial intelligence around the world. By 2023, AI-driven applications had contributed significantly to multi-billion-dollar industries, with the AI hardware sector projected to grow at a compound annual growth rate (CAGR) exceeding 20% by 2030[4]. However, with the rapid development of artificial intelligence, the traditional network architecture and system have been challenged unprecedented [5]. For example, training models like GPT-3 with 175 billion parameters consumes around 1287 MWh of energy, with demands escalating as models grow larger [6]. As AI systems expand, the separation of memory and processing in von Neumann architectures leads to energy inefficiencies and increased latency, exacerbating the von Neumann bottleneck [7–11]. To support the continued growth of AI, there is an urgent need for more efficient computing paradigms that can handle increasing complexity while minimizing power consumption.

Inspired by the structure and function of biological neural networks, neuromorphic computing has emerged as a promising solution [12–17]. Artificial synaptic devices, endowed with integrated sensing, memory, and neuromorphic functionalities, have become the core components of neuromorphic computing, with significant research progress achieved [18]. This approach offers a new avenue to overcome the limitations of the von Neumann architecture in terms of energy efficiency and computational performance [19]. Initially, synaptic devices could only regulate conductance through simple voltage control, resulting in limited performance and flexibility [20]. With further research, optoelectronic synergistic control was introduced into synaptic devices [21–23], typically employing optical writing and electrical erasing to achieve synaptic behaviors. However, the dual-signal approach introduced additional complexities, such as Joule heating and physical deformation of the devices [24]. To overcome this limitation, all-optical controlled (AOC) synaptic devices have become a crucial area of current research [25].

Compared with conventional electrically controlled synaptic devices and optoelectronic hybrid synapses, AOC synaptic devices enable bidirectional modulation of device conductance solely through optical pulses, allowing both optical writing and optical erasing of synaptic states. This fully optical operation fundamentally eliminates Joule heating, electrical crosstalk, and parasitic power dissipation associated with current-driven synapses, thereby enabling ultralow energy consumption. In addition, optical signals intrinsically offer extremely high bandwidth and fast response speeds, allowing AOC synaptic devices to operate on nanosecond-to-microsecond timescales. Wavelength-selective optical excitation further introduces an additional degree of freedom for synaptic modulation, enabling bidirectional conductance control, multi-level weight states, and parallel addressing without increasing circuit complexity. More importantly, AOC synaptic devices integrate sensing, memory, and computation within a single device architecture, enabling in-sensor perception and preprocessing of visual information at the sensor level. This significantly reduces system integration complexity and is particularly advantageous for large-scale neuromorphic arrays, in-sensor computing, and biomimetic vision systems. Table 1 summarizes the key differences among conventional electrical synapses, optoelectronic hybrid synapses, and fully optically controlled synaptic devices. Owing to all-optical operation, AOC synaptic devices outperform conventional counterparts in terms of energy efficiency, response speed, and ease of integration. In particular, eliminating electrical programming not only reduces power consumption and thermal effects but also suppresses signal interference and simplifies array-level interconnections. Collectively, these attributes position AOC synaptic devices as a promising hardware platform for next-generation low-power, high-speed, and highly integrated neuromorphic computing systems.

**Table 1:** Comparison of electrical, optoelectronic hybrid, and all-optical controlled synaptic devices

Feature	Electrical synaptic devices	Optoelectronic hybrid synaptic devices	All-optical controlled synaptic devices
Input stimulus	Electrical pulses only	Electrical and optical signals	Optical signals only
Synaptic weight control	Voltage-driven	Optical writing and electrical erasing (or vice versa)	Optical writing and optical erasing
Energy consumption per event	pJ-nJ, depending on device type and circuitry	fJ-pJ, set by photo-carrier modulation and low-power gating	fJ-pJ, energy scales with illumination and bias
Response speed	μs-ms	μs-ms	ns-μs
Device complexity	Simple device, complex wiring	Increased circuit complexity	Simplified interconnect, wavelength multiplexing
Integration density	Limited by wiring	Limited by hybrid routing	High-density, optics-friendly integration
Sensing- memory-computing integration	Separated	Partially integrated	Fully integrated (in-sensor computing)
Neuromorphic system compatibility	Electrical neuromorphic systems	Transitional architecture	Optical neuromorphic and vision systems
Key advantage	Mature fabrication (CMOS compatible)	Non-contact writing, multi-modal sensing	Parallelism, high speed, low latency, non-contact control

**Fig. 1:**
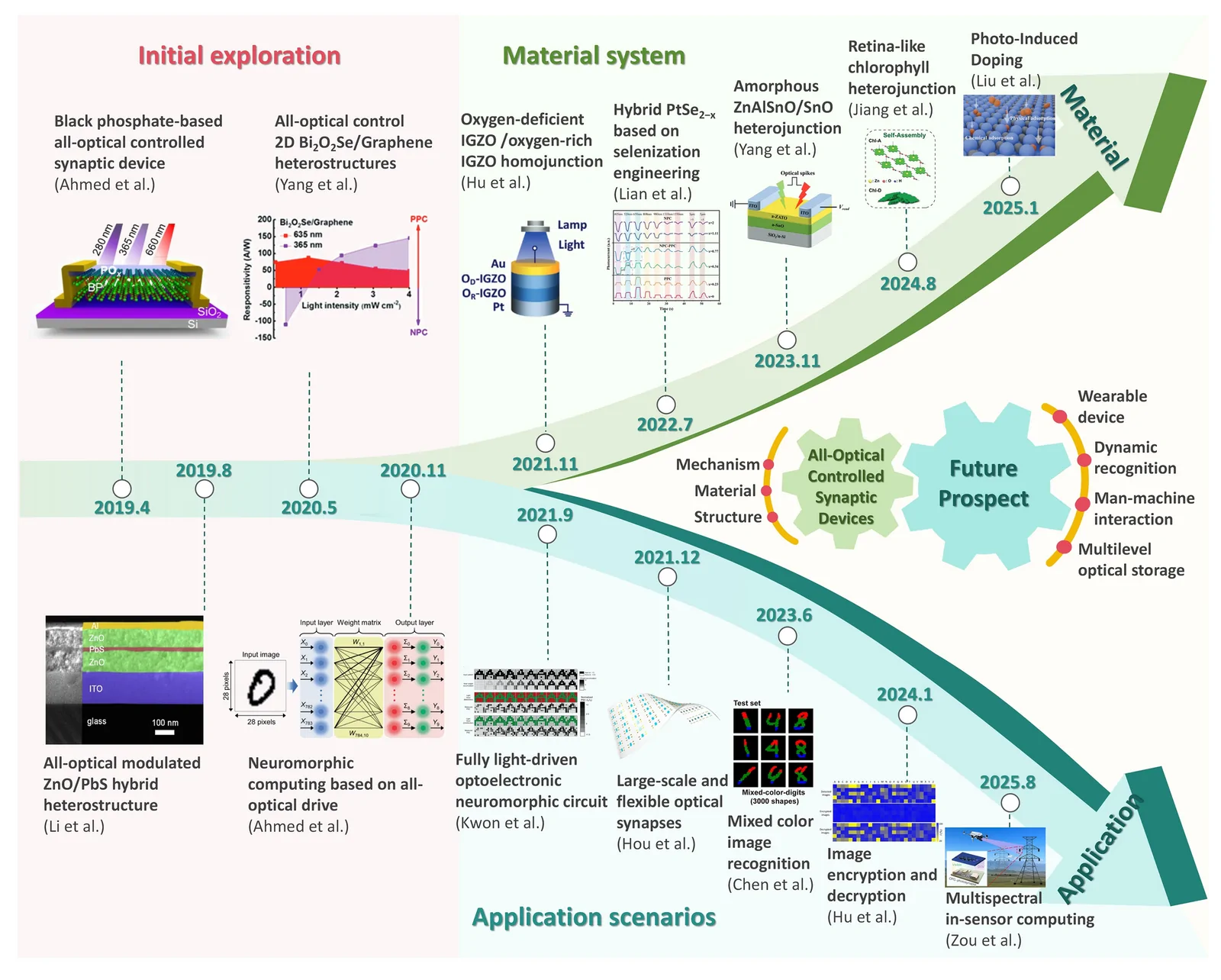
Development of AOC synaptic devices during 2019–2025 Copyright 2021, American Chemical Society [24]; Copyright 2019, WILEY‐VCH Verlag GmbH & Co. KGaA, Weinheim [27]; Copyright 2019, Elsevier Ltd [28]; Copyright 2020, WILEY–VCH Verlag GmbH & Co. KGaA, Weinheim [29]; Copyright 2020, Wiley–VCH GmbH [30]; Copyright 2021, Wiley–VCH GmbH [31]; Copyright 2022, Wiley–VCH GmbH [32]; Copyright 2023, American Chemical Society [33]; Copyright 2020, The Authors. Advanced Functional Materials published by Wiley–VCH GmbH [34]; Copyright 2024, Wiley–VCH GmbH [35]; Rights managed by AIP Publishing [36]; Copyright 2023, Wiley–VCH GmbH [37]; Copyright 2025, American Chemical Society [38]; Copyright 2024, Wiley–VCH GmbH [39]

In 2018, Han et al*.* [26] reported a graphene-semiconductor heterojunction that, for the first time, demonstrated bidirectional (positive and negative) photoresponses under illumination at different wavelengths. This work laid a foundational basis for the development of optoelectronic synaptic devices exhibiting wavelength-dependent bidirectional photoactivity. In 2019, Ahmed et al*.* [27] introduced a photonic synaptic device based on black phosphorus, marking the first proposal of an AOC synapse. Notably, this device could emulate both excitatory and inhibitory synaptic behaviors under 280 nm and 365 nm light stimuli, respectively, without the need to reverse the polarity of the applied electric field. Also in 2019, Li et al*.* [28] simulated both long- and short-wavelength-induced synaptic excitation and inhibition in a ZnO/PbS neuromorphic device. Ultraviolet light triggered excitatory plasticity, while infrared light-induced inhibitory effects, and several other characteristic synaptic functionalities were also emulated. Since 2020, AOC synaptic devices have witnessed substantial advances across material systems, device architectures, mechanistic understanding, and neuromorphic computing applications, highlighting the dynamic evolution of this emerging field (Fig. 1) [24, 27–39]. The operation of AOC synaptic devices in the visible range with three primary colors of red, green, and blue (RGB) is important for artificial neural networks [37]. A broad range of material platforms, such as oxide semiconductors, low-dimensional materials, perovskites, and organic compounds, have been explored for their ability to support AOC synaptic behavior. These materials have enabled diverse and task-specific synaptic functionalities, facilitating progress from logic processing and behavioral emulation to visual perception and bioinspired visual systems. Collectively, these developments underscore the potential of AOC synaptic hardware in next-generation neuromorphic computing paradigms.

The AOC synapse is a very novel and creative issue, which is now at the early stage of its development. Rapid progress has occurred in this field in recent years. To date, however, a thorough, universally recognized underlying framework is still absent to encompass both the inherent mechanisms and design strategies of AOC devices, which has hindered the broader development of this discipline. Existing classifications are predominantly material- or mechanism-oriented and often focus on unidirectional synaptic responses, which are insufficient to guide scalable device design, and system-level integration. This gap makes it essential to summarize and review the latest advancements, particularly discussing their bidirectional optical response mechanism, material design principles and algorithms-devices co-development, which have not been adequately addressed in existing literatures. To achieve neuromorphic computing based on AOC synaptic devices, in-depth research on the interaction between external stimuli and materials is required, as well as new insights into physics, materials, device structures, applications, and integration technologies. It is also necessary to summarize in detail the combination mode of the algorithm and AOC synaptic devices, which is the core essence of the development of neuromorphic computing. This review provides a systematic introduction of all-optical controlled synaptic devices for neuromorphic computing in recent years, with detailed analyses of structures, materials, mechanisms, and applications. Most importantly, it proposes a forward-looking strategy of integrating mechanisms, device design, material selection, structure optimization, algorithm collaboration, and application scenarios, filling the gaps in related research. The underlying framework proposed in this work is expected to greatly accelerate the development of high-performance, low power, and scalable AOC synaptic devices, providing an ideal hardware platform for neuromorphic computing.

## Device Characteristics and Performance Metrics

**Fig. 2:**
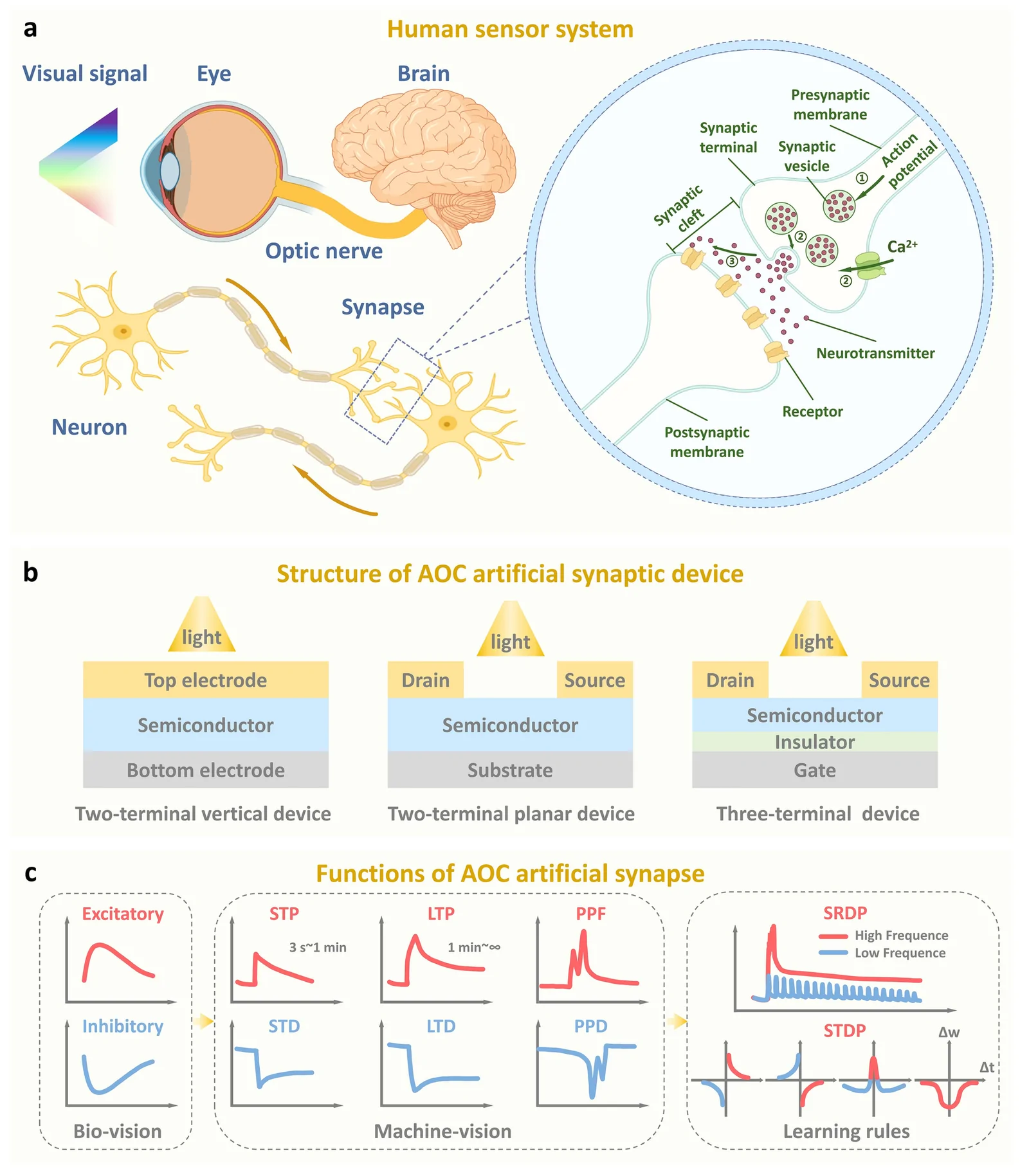
Device Characteristics of AOC synaptic device. **a** Schematics of a biological synapse. **b** Structure of AOC artificial synaptic device. **c** Functions of AOC artificial synapse

AOC artificial synaptic devices simulate biological synapses to process and store information [40]. Synapses, fundamental units in the nervous system, facilitate signal exchange between neurons through a detailed chemical and electrical signaling process, as depicted in Fig. 2a. Briefly, a strong stimulus prompts the presynaptic neuron to generate an action potential, which travels to the synaptic terminal. The action potential opens voltage-gated Ca^2+^ channels, causing Ca^2+^ influx and neurotransmitter release into the synaptic cleft. Neurotransmitters bind to postsynaptic receptors, altering ion channel states and membrane potential. Depending on the type of neurotransmitter and receptor involved, synaptic function can be either excitatory or inhibitory.

### Device Structure

In particular, the AOC synaptic device replicates synaptic plasticity, a bionic device feature that enables neural computing functions. It utilizes optical rather than electrical signals to modulate synaptic behavior. The structure of a two- or three-terminal AOC synaptic device is depicted in Fig. 2b. As an example, in the two-terminal planar synaptic device structure, the source and drain electrodes correspond to the presynaptic and postsynaptic membranes, respectively. The light pulse can be regarded as acting on the presynaptic action potential, while the carriers involved in conductivity in the active material between the two electrodes are equivalent to neurotransmitters. Based on the photoelectric effect, the light signal stimulates the active material to produce photogenerated electron and hole pairs. The transport and accumulation of these carriers in the device mimic the signaling of biological synapses. The wavelength, intensity, frequency, and duration of the light signal directly influence the enhancement and decrease of the electrical conductivity of the active material, realizing the synaptic response.

In terms of device structure, the choice between two-terminal and three-terminal architectures is crucial and largely depends on the target application. To provide a comprehensive guide for device design, we categorize AOC devices into three distinct architectures: two-terminal vertical, two-terminal planar, and three-terminal structures (see Table 2). Two-terminal vertical devices, such as memristors, typically exhibit a vertical metal–insulator–metal (MIM) stack structure. Due to their simple cross-point geometrical structure, they have the highest potential in terms of integration density and three-dimensional stacking. This makes them particularly suitable for constructing high-density cross-strip arrays to achieve large-scale parallel vector–matrix multiplication (VMM) and large-scale pattern recognition tasks. However, they often face challenges such as signal crosstalk and limited linearity of synaptic weight updates. While three-terminal devices, such as transistors, introduce a gate terminal, which provides additional freedom for precisely adjusting channel conductivity. Compared to the two-terminal vertical architecture, they offer better linearity, weight update symmetry, and signal-to-noise ratio. They are more suitable for applications requiring high precision and complex signal processing. However, gate control still has certain complexity. Therefore, a two-terminal planar device structure without a gate can also be adopted. This structure is simpler, but at the cost of lower integration density. It is suitable for in-sensor computing, adaptive visual perception systems, and other scenarios. In practical usage scenarios, the appropriate device architecture should be selected based on physical mechanisms, integration density, and functional controllability.

**Table 2:** Structural characteristics, operational merits, and application scopes of different AOC synaptic device architectures

Device architecture	Structural configuration	Key structural characteristics	Operational merits and limitations	Typical application scenarios
Two-terminal vertical	Sandwich geometry: The active layer is vertically stacked between a top and bottom electrode	Minimal device footprint	Highest integration density and 3D stackability	High-density crossbar arrays
		Short carrier transit distance	Fast vertical carrier transport	Large-scale vector–matrix multiplication
		Encapsulated active region	Prone to sneak-path currents in arrays	Neural networks requiring massive connectivity
Two-terminal planar	Lateral geometry: Two electrodes are deposited laterally on the surface of the active material. Light directly illuminates the exposed channel surface between the electrodes	Simple fabrication Lithography-friendly	Maximum optical exposure and absorption efficiency	In-sensor computing with pre-processing
		Large light–matter interaction area	Ease of fabrication and material characterization	Proof-of-concept synaptic emulation
		Surface-dominated mechanism	Low integration density	Flexible electronics
Three-terminal	Field-effect geometry: Consists of source, drain, and gate electrodes. The active channel connects source and drain, with conductance modulated by both light and the gate electric field	Gate terminal adds a third control node	Excellent signal-to-noise ratio and linearity	Logic-in-memory circuits
		Physically separated write (gate/light) and read (drain) paths	Gate-tunable sensitivity and gain	High-precision artificial vision systems
				Spiking neural networks
			Suppressed sneak currents	
			Complex circuit design and larger footprint	
		Planar or vertical channel variants		

### Basic Functionalities

The strength of a neuron’s signal depends on its synaptic weights with other neurons, which dynamically adjust through a process known as synaptic plasticity [41]. Synaptic plasticity is essential for neural systems, enabling learning, memory, image recognition, and reasoning by modifying synaptic weights [42–44]. AOC synaptic devices use light pulses to simulate synaptic plasticity [45]. According to Hebbian’s theory, short-term plasticity (STP), long-term plasticity (LTP), spike-timing-dependent plasticity (STDP), and spike-rate-dependent plasticity (SRDP) are typical forms of synaptic plasticity. Figure 2c depicts the basic synaptic function of the AOC device. STP and LTP are fundamental in adjusting the synaptic strength over different time scales. STP involves rapid, temporary changes in synaptic strength, helping synapses adjust dynamically to neural activity, marked by phenomena like paired-pulse facilitation (PPF) and depression (PPD). Over time, repeated stimuli can convert STP into LTP. AOC devices control this transition by varying the frequency, number, or duration of optical pulses. These mechanisms ensure that synaptic modulation is finely tuned to both individual and collective neural dynamics.

### Evaluation Metrics

To effectively simulate the synaptic functions, AOC synaptic devices must not only emulate the dynamic behaviors of biological synapses but also fulfill specific performance criteria that demonstrate their efficiency and functionality within complex neural networks. The following outlines the main indicators used to evaluate the performance of AOC synaptic devices.

*Excitatory Postsynaptic Current *(*EPSC*)* and Inhibitory Postsynaptic Current* (*IPSC*): EPSC indicates a positive current similar to biological excitatory responses, while IPSC represents a negative current, mimicking inhibitory synaptic behavior. The decay of the final EPSC / IPSC induced by successive stimuli can be fitted by the following Kohlrausch stretched exponential function as in Eq. (1) [46]:

1 $$I\left(t\right)={I}_{0}\exp\left[-{\left(\frac{t}{\tau }\right)}^{\beta }\right]+{I}_{\infty }$$

$I\left(t\right)$ represents the decay function, where τ is the relaxation time constant, t is the time during the decay process, ${I}_{0}$ is the preexponential factor, ${I}_{\infty }$ is the final value of the current representing the decay, and β is the stretch index ranging from 0 to 1. In certain cases, the decay of EPSC/IPSC follows a bi-exponential behavior, which can be described by Eq. (2):

2 $$I\left( t \right) = A \times {\mathrm{exp}}\left( { - \frac{t}{{\tau _{1} }}} \right) + B \times {\mathrm{exp}}\left( { - \frac{t}{{\tau _{2} }}} \right) + I_{\infty }$$

Here A and B are constants representing the initial amplitudes of the rapid and slow decay components, respectively, while ${\tau }_{1}$ and ${\tau }_{2}$ denote the relaxation time constants. The biexponential model provides a more explicit representation of two distinct and separable relaxation processes within the system. Both models are instrumental in deciphering the decay dynamics of EPSC/IPSC.

*Paired-Pulse Facilitation *(*PPF*)* Index*: PPF is an important feature of STP in AOC synaptic devices, which refers to the phenomenon that when a device receives two consecutive optical stimuli, the response triggered by the second stimulus is enhanced compared to the first. The PPF index is calculated by comparing the magnitude of the response triggered by the two pulses (Eq. (3)) [47]:

3 $$\mathrm{PPF}/\text{PPD Index}= \frac{{A}_{2}}{{A}_{1}}\times 100\%$$

where ${A}_{1}$ and ${A}_{2}$ stand for the amplitude of the postsynaptic currents after the first and second stimuli, respectively. Moreover, the PPF index (A_2_/A_1_), a parameter that measures the gain in the postsynaptic current (PSC), follows a double-exponential function for its dependence on the time interval as in Eq. (4) [48]:

4 $${\mathrm{PPF}}/{\text{PPD Index}} = C_{1} {\mathrm{exp}}\left( { - \frac{{\Delta t}}{{\tau _{1} }}} \right) + C_{2} {\mathrm{exp}}\left( { - \frac{{\Delta t}}{{\tau _{2} }}} \right) + 1$$

where ${C}_{1}$ (${C}_{2}$) and ${\tau }_{1}$ (${\tau }_{2}$) denote the degree of initial ease and the characteristic relaxation time of the rapid (slow) phase, respectively. This metric helps in assessing the capability of AOC devices to emulate the complex temporal dynamics of biological synaptic activity.

*Conductance Modulation*: Conductance modulation is a crucial metric for evaluating the dynamic behavior of AOC synaptic devices. It involves the reversible adjustment of conductivity through external optical signals, enabling both excitatory and inhibitory behaviors. In neuromorphic computing, conductance modulation is vital for accurately and efficiently recognizing patterns. The LTP/LTD modulation curves serve as the cornerstone of conductance weight modulation, and nonlinearity and symmetry are key parameters affecting device performance.

Nonlinearity is a critical parameter used to describe the deviation of actual conductance changes from an ideal linear relationship during conductance modulation. The nonlinearity of LTP/LTD curves is typically derived by fitting the weight update process using Eqs. (5 and 6):

5 $$G_{{n + 1}} = G_{n} + \Delta G_{{\mathrm{P}}} = G_{n} + \alpha _{{\mathrm{P}}} {\mathrm{exp}}\left( { - \beta _{{\mathrm{P}}} \frac{{G_{n} - G_{{{\mathrm{min}}}} }}{{G_{{{\mathrm{max}}}} - G_{{{\mathrm{min}}}} }}} \right)({\mathrm{LTP}})$$

6 $$G_{{n + 1}} = G_{n} + \Delta G_{{\mathrm{D}}} = G_{n} + \alpha _{{\mathrm{D}}} {\mathrm{exp}}\left( { - \beta _{{\mathrm{D}}} \frac{{G_{{{\mathrm{max}}}} - G_{n} }}{{G_{{{\mathrm{max}}}} - G_{{{\mathrm{min}}}} }}} \right)({\mathrm{LTD}})$$

In this Eq., ${G}_{n+1}$ and ${G}_{n}$ denote the synaptic conductance after the n+1-th and n-th pulse, respectively, while α and β represent the conductance step size and nonlinearity degree.

Additionally, the symmetry of LTP/LTD curves reflects the balance of conductance modulation. Symmetry (${S}_{\mathrm{sym}}$) is defined as the reciprocal of the symmetry error to quantify the goodness of symmetry, and the expression is given in Eq. (7). The symmetry error (${\varepsilon }_{\mathrm{sym}}$) is then obtained by calculating the difference between the LTP and LTD modulation curves, as derived from Eq. (8):

7 $${S}_{\mathrm{sym}}=\frac{1}{{\varepsilon }_{\mathrm{sym}}}$$

8 $${\varepsilon }_{\mathrm{sym}}={\sum }_{k=1}^{k=n}\frac{{({G}_{N}(k)-{G}_{N}(2n-k))}^{2}}{n}={\sum }_{k=1}^{k=n}\frac{{(G(k)-G(2n-k))}^{2}}{{n({G}_{\mathrm{max}}-{G}_{\mathrm{min}})}^{2}}$$

where ${G}_{N}(k)=\frac{G(k)-{G}_{\mathrm{min}}}{{G}_{\mathrm{max}}-{G}_{\mathrm{min}}}$ and ${G}_{N}$ denotes the standard conductance. A near-zero symmetry error indicates highly balanced LTP and LTD behaviors, signifying equivalent capabilities for synaptic enhancement and suppression. Conversely, significant asymmetry may introduce imbalances in information processing.

For conductance modulation curves, reducing nonlinearity and enhancing symmetry significantly improve pattern recognition accuracy and the stability of weight updates in neuromorphic computing.

*Reproducibility*: Reproducibility refers to the consistency of LTP/LTD modulation curves across multiple cycles and devices under identical experimental conditions. It is assessed through cycle-to-cycle variation (CCV) and device-to-device variation (DDV).

CCV describes the writing noise of LTP and LTD cycles, defined as the standard deviation of channel conductance measured over multiple cycles. DDV assesses the uniformity across different devices, reflecting the consistency of manufacturing processes and scalability for production. Lower CCV and DDV values signify higher reproducibility. In neural networks, this reduces cumulative errors in information storage and weight updates, enhancing accuracy in pattern recognition and learning tasks.

*Energy Consumption*: Energy consumption is a critical metric for artificial synapses. Low power consumption is the goal pursued by AOC synaptic devices, considering the computational cost of developing more energy-efficient ones. Compared to conventional complementary metal oxide semiconductor (CMOS) circuits, the energy consumption for triggering a biological synaptic event is only 1–100 fJ [49]. Currently in AOC synaptic devices, two main methods are commonly used to calculate energy consumption [50, 51]. The first is the energy of the electrical response, which only considers the electrical response energy consumption of the device under light stimulation and is used to evaluate the direct electrical energy consumption generated by the operation of the device (Eq. (9)). The second is the energy of the optical spike, which calculates the energy consumed according to the energy of the light spike itself (Eq. (10)), quantifying the energy consumed by the light source to trigger the synaptic response.

9 $$dE=V\cdot I\cdot dt$$

10 $$dE=P\cdot S\cdot dt$$

where V is the read voltage applied to the device, I is the device current during a spike with the duration of t, dt is the event duration (spike duration), P is the power density of the optical stimulus, and S is the effective area of the device. In most of the literature, scholars usually regard the energy of the electrical response as energy consumption and use the energy of the optical spike to evaluate the sensitivity of the photoelectric synaptic device. It has been shown that reducing the size of the device and the duration of the optical spikes can significantly reduce energy consumption [52–54].

*Photocurrent* (${I}_{\mathrm{ph}}$): In AOC synaptic devices, photocurrent is defined as ${I}_{\mathrm{ph}}= {I}_{\mathrm{illumination}}-{I}_{\mathrm{dark}}$, where ${I}_{\mathrm{illumination}}$ represents the current under light exposure, and ${I}_{\mathrm{dark}}$ is the baseline current when no light is present. This parameter quantifies the device's sensitivity to optical stimuli, reflecting the change in current due to light-induced processes.

*Light-to-Dark Ratio* (*LDR*): Another important characteristic of AOC synaptic devices is the light-to-dark ratio, which is typically expressed as the ratio of the maximum to minimum conductance (${G}_{\mathrm{max}}/{G}_{\mathrm{min}}$) in cyclic modulation curves. This ratio represents the dynamic range of the device's response under different illumination conditions, where a higher LDR indicates stronger optical modulation capability and enhanced synaptic behavior.

*Responsivity* (R): Responsivity measures the efficiency of converting optical signals into electrical signals, defined as the ratio of photocurrent (${I}_{\mathrm{ph}}$) to the incident optical power (P) per unit area (A) as in Eq. (11):

11 $$R=\frac{{I}_{\mathrm{ph}}}{P\times A}$$

This metric reflects the device's sensitivity to light, with higher values indicating greater capability to detect and respond to minimal light intensities.

## Mechanism

The distinction between AOC synaptic devices and optoelectronic synaptic devices lies in the bidirectional modulation of device conductance, which is exclusively influenced by light. These devices utilize the unique properties of light to regulate synaptic behaviors. The conductivity of the device is seen as the weight of the synapse, and the plasticity of the synapse, including enhancement and inhibition, is embodied as positive photoconductivity (PPC) and negative photoconductivity (NPC), respectively. PPC reflects the increase in device conductance under illumination, primarily determined by the material’s ability to sustain a high density of free carriers under light exposure. The PPC mechanism is relatively straightforward and widely observed in semiconductors or heterostructures with appropriate defect states or trap levels. Specifically, PPC is driven by the persistent photoconductivity effect, wherein the material absorbs photon energy to produce electron–hole pairs. These carriers are often trapped in shallow or localized states, delaying recombination and maintaining an elevated free carrier density in the conduction band. This leads to a prolonged increase in conductivity, which persists even after removing optical stimulation. NPC, on the other hand, represents a decrease in conductance under illumination and is a more complex phenomenon crucial for achieving bidirectional modulation. The realization of both PPC and NPC often involves mechanisms such as interfacial charge dynamics, trap-assisted processes, and photo-induced effects. In the case of PPC, photoexcitation promotes carrier generation, separation, or accumulation, which leads to an increase in free carrier density and enhanced conductivity. This process typically originates from the photoelectric effect, where the material absorbs photons to generate electron–hole pairs, thereby enhancing conductivity. In contrast, NPC arises when photo-induced processes predominantly facilitate carrier recombination, extraction, localization, or interfacial barrier modulation, resulting in a net reduction of effective conducting carriers and decreased conductivity.

**Fig. 3:**
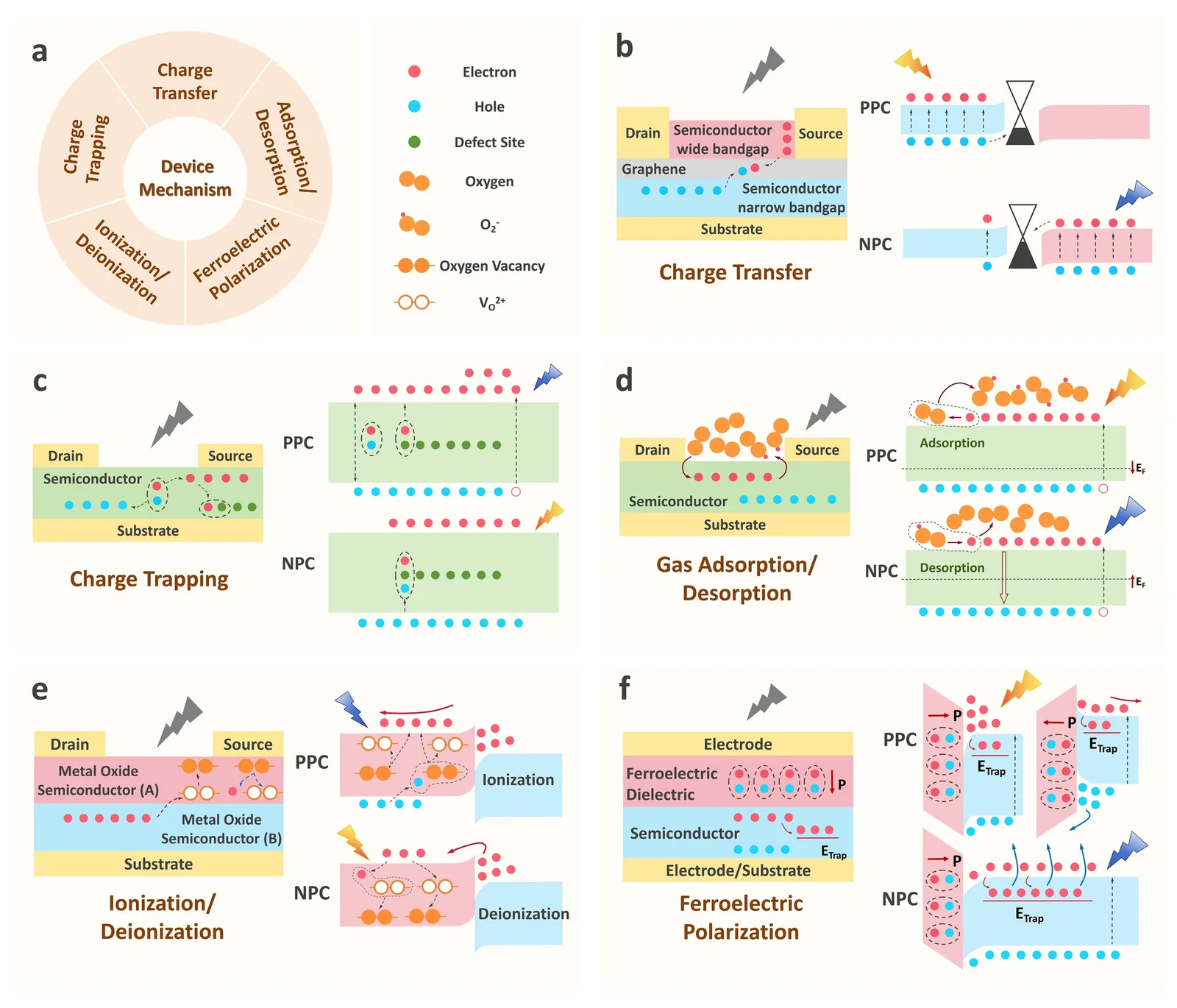
Schemes of structures of mechanism based on AOC synaptic device. **a** Mechanism classification. **b** Charge transfer mechanism. **c** Defect trapping mechanism. **d** Oxygen adsorption/desorption mechanism. **e** Oxygen vacancies ionization/deionization mechanism. **f** Ferroelectric polarization mechanism

The synergistic interplay of PPC and NPC enables AOC synaptic devices to emulate the bidirectional plasticity of biological synapses under all-optical control. Thus, AOC synapses enable both optical writing and optical erasing of information within a single device. In this section, we categorize the bidirectional photoresponse physical mechanisms based on AOC synaptic devices into five types: charge transfer, charge trapping, gas adsorption/desorption, oxygen vacancies ionization/deionization, and ferroelectric polarization (Fig. 3a). We will delve into these mechanisms that support the bidirectional optical response of AOC synaptic devices, providing a comprehensive analysis of how light modulation excitation/ inhibition synaptic behavior.

### Charge Transfer

In heterostructures, photo-induced charge transfer between layers can alter the interfacial charge density and electric field distribution [55, 56]. Therefore, band structure engineering and adjustment are key techniques for controlling the transfer of photogenerated carriers [57]. A heterojunction is formed by the interface region between two different semiconductor materials, and it has been widely demonstrated to enhance charge separation and promote charge transfer effectively [58–60]. The relative positions of the conduction band and valence band between the two semiconductors create potential barriers at the interface, which significantly impact the transfer and distribution of charges. Currently, the common structure of AOC synaptic devices primarily consists of p-n heterojunctions. Type-II heterojunctions, especially type Z heterojunctions, are particularly well suited for precisely simulating synaptic behavior due to their effective control over the capture and recombination of photogenerated carriers.

The characteristic of a Type-II heterojunction is the staggered alignment where the minimum of the conduction band (CBM) and the maximum of the valence band (VBM) of two materials overlap each other. This band structure configuration optimizes the effective separation of electrons and holes upon photoexcitation. Moreover, the distinct bandgaps of the two semiconductors give rise to wavelength-dependent differences in optical absorption, enabling bidirectional photoresponse at specific wavelengths. As illustrated in Fig. 3b, in a planar-stacked heterojunction, wavelength-selective PPC and NPC can be achieved by exploiting the characteristic absorption spectra of the constituent semiconductors. Specifically, one semiconductor with a larger bandgap predominantly absorbs photons in the visible range, whereas the other, possessing a smaller bandgap, primarily absorbs infrared radiation. In such architectures, graphene is typically employed as the channel layer. Owing to the work function mismatch between the constituent materials, band bending is induced at the interfaces, resulting in a built-in electric field. Under long-wavelength illumination, the narrow bandgap semiconductor dominates the absorption process, generating electron–hole pairs. The built-in electric field drives the photogenerated holes toward the graphene layer, enhancing hole transport in graphene and leading to an increase in channel conductance, which corresponds to PPC. In contrast, under short-wavelength illumination, the wide bandgap semiconductor governs the optical absorption. Excess electrons are transferred into graphene under the influence of the built-in electric field, suppressing hole transport and thereby reducing the channel conductance, giving rise to NPC. Therefore, by rationally engineering heterojunctions with tailored bandgaps and optical absorption characteristics, particularly by leveraging carrier migration driven by band alignment, the bidirectional photoresponse of AOC devices can be precisely programmed, enabling wavelength-selective optical sensing.

Recent research has demonstrated that these heterojunction mechanisms are extensively integrated into large-scale and flexible neuromorphic devices. Hou et al*.* [24] designed a synaptic device based on a heterostructure consisting of pyrenyl graphdiyne (Pyr-GDY), graphene, and PbS quantum dots. This arrangement exhibits an ultra-responsive bidirectional photoresponse under optically controlled effects, enabling the optical synapses to simulate both excitatory and inhibitory synaptic behaviors within optical pathways. Additionally, combining graphene with organic semiconductors, such as C_60_/pentacene [26], significantly enhances photoresponsivity across a broad spectral range from visible to near-infrared. The unique arrangement of energy bands within these heterojunctions facilitates a bidirectional photoresponse, thus enabling advanced functionalities such as hyperspectral imaging and image sensing.

### Charge Trapping

The charge capture mechanism is integral to the operation of optoelectronic synaptic devices, particularly in regulating the conductivity of materials. Charge trapping is generally categorized into barrier accumulation and defect trapping, with this discussion focusing primarily on the defect trapping mechanism. Semiconductor defects can be classified into surface defects, interface defects, and bulk defects [61–64]. These defect traps are often associated with impurities or lattice imperfections, and their presence can markedly influence the electronic characteristics of the material. Defects are typically regarded as localized energy states located within the bandgap or in proximity to the conduction or valence bands [65]. These defect states serve as trap centers for charge carriers, such as electrons and holes, facilitating the capture and subsequent release of these carriers [66, 67].

When defect states are present, a fraction of the photogenerated carriers can be trapped by these states, as schematically shown in Fig. 3c. Under short-wavelength illumination or high optical power density, a large number of electron–hole pairs are generated. High-energy photons facilitate the rapid release of carriers previously trapped in defect states, while newly generated carriers are less likely to be captured. As a result, the concentration of free carriers in the channel increases, giving rise to a positive photocurrent and thus PPC. In this regime, the PPC behavior is dominated by carrier release from trap states. By contrast, under long-wavelength illumination or low optical power density, the photon energy is relatively low, and the excited carriers possess insufficient energy. These photogenerated carriers are more readily captured by defect states, leading to a reduction in the free carrier concentration within the channel and resulting in NPC. In this case, the NPC behavior is governed primarily by carrier trapping. Therefore, the interplay between carrier trapping and release processes, modulated by the nature of defect states and illumination conditions, enables the material to exhibit complex bidirectional photoconductive behavior. This mechanism highlights the feasibility of employing defect engineering as an effective strategy for realizing ambipolar optoelectronic synaptic devices based on AOC architectures.

In defect-dominant devices, the defect capture mechanism can trigger novel functionalities. For instance, black phosphorus (BP) spontaneously generates a significant number of defect states, and different ultraviolet wavelengths can stimulate the generation of both positive and negative photoconductivity, simulating excitatory and inhibitory action potentials in synapses [27, 30, 68]. Multilayer BP readily forms a 3–4 nm-thick P_x_O_y_ oxide layer on its surface when exposed to ambient conditions. This oxidation layer introduces a high density of localized charge trap states within the bandgap of BP. These trap states, energetically distributed between the conduction band and the valence band, provide physical sites for the capture and storage of photogenerated carriers. Under high-energy ultraviolet illumination at 280 nm, BP is excited to generate a large population of hot carriers, while the absorbed photon energy simultaneously promotes the rapid release of carriers previously trapped in the defect states. This process manifests as PPC, corresponding to the data writing operation. In contrast, under lower-energy ultraviolet illumination at 365 nm, the photogenerated carriers are more susceptible to capture by the oxidation-induced trap states, resulting in NPC and enabling data erasure. Geng et al*.* [69] identified sulfur vacancies (${V}_{S}$) and zinc interstitials (${Zn}_{i}$) in the defect structure of ZnS films as key capture sites for the trapping and releasing of carriers, achieving STP and STD. These findings demonstrate that the defect capture mechanism provides unique regulatory capabilities for optoelectronic synaptic devices, enabling dynamic responses to light stimuli and facilitating the modulation of conductivity and synaptic behavior.

### Gas Adsorption/Desorption

Similar to the changes in electron capture and re-capture centers induced by light stimulation, gas molecules can also act as electron capture and re-capture centers in AOC synaptic devices [70, 71]. Oxygen, as the most prevalent environmental gas, interacts with semiconductor surfaces via physisorption or chemisorption, with its adsorption and desorption processes significantly influencing the bidirectional photoconductivity behavior. Figure 3d shows the working mechanism of oxygen adsorption and desorption processes. Liang et al*.* [72] reported a device based on graphene/TiO_2_ quantum dots that achieves all-optical control of artificial synapses through light-induced gas adsorption and desorption effects. In this device, the conductivity of the graphene layer is modulated by the quantity of oxygen molecules adsorbed on the TiO_2_ quantum dots, which can be reversibly controlled via different wavelengths of light stimulation.

Oxygen adsorption typically occurs in the absence of light or under specific wavelength illumination. In dark conditions, oxygen molecules are stabilized by interacting with surface defects, such as oxygen vacancies, within the semiconductor [73]. Specifically, oxygen molecules seek defect sites for non-dissociative adsorption, forming superoxide ions (${O}_{2}^{-}$) and enhancing the conductivity of the semiconductor through electron transfer processes. For long-wavelength light pulses, the photon energy is insufficient to induce desorption, allowing oxygen molecules to remain adsorbed on the surface. In this context, oxygen acts as an electron acceptor, facilitating the transfer of conduction band electrons to the adsorbed oxygen molecules to form ${O}_{2}^{-}$ (Eq. (12)):

12 $${O}_{2}+{e}^{-}\to {O}_{2}^{-}$$

This process can be viewed as the transition of oxygen molecules from physisorbed to chemisorbed states, resulting in p-type doping of the active material, increasing hole concentration at the surface, and leading to a downward shift in the Fermi level, thereby enhancing conductivity and generating the PPC phenomenon.

Under short-wavelength laser irradiation, the photon energy disrupts the chemical bonds between ${O}_{2}$ and the material surface, causing the adsorbed ${O}_{2}$ to desorb and release back into the gas phase. This process is accompanied by the migration of photogenerated electrons, which increases the electron concentration, further enhancing the n-type doping effect and raising the Fermi level. The recombination of photogenerated electrons with holes subsequently leads to the NPC phenomenon. The light-induced adsorption and desorption mechanisms of oxygen molecules often interplay with the intrinsic defects present in the material [74]. Dissociation of oxygen molecules near the selenium vacancy in PdSe_2_ has been shown to introduce localized states near the conduction band, resulting in NPC behavior of PdSe_2_ [75]. In addition, Liu et al*.* [39] found that the surface oxygen of SnSe materials is jointly regulated by physical adsorption and chemical adsorption. The dominant behavior of chemical adsorption brings high stability, while physical adsorption provides the possibility for the realization of NPC through light control.

The gas molecule adsorption and desorption processes induced by light stimulation present a promising avenue for achieving all-optical control of artificial synapses. By adjusting the wavelength, intensity, and duration of light exposure, the adsorption and desorption of oxygen molecules on the material surface can be precisely controlled, thereby regulating the conductivity of the device.

### Oxygen Vacancies Ionization/Deionization

As a common type of defect, oxygen vacancies (${V}_{o}$) can absorb and release photon energy, generating free electrons or holes within semiconductor materials. This process plays a critical role in the storage and dynamic modulation of neuromorphic computing. The state transitions during the ionization and deionization of oxygen vacancies significantly impact the electrical conductivity and optoelectronic properties of the material. In recent years, extensive research has been conducted on the ionization and deionization phenomena of oxygen vacancies in various materials, such as oxide semiconductors and perovskites [22, 76–78]. These charge modulation mechanisms provide a crucial foundation for the multifunctionality of AOC synaptic devices.

Oxygen vacancies in oxide semiconductors typically exist in either a neutral ground state (${V}_{o}$) or a metastable ionized state (${V}_{O}^{2+}$ or ${V}_{O}^{+}$). Transitions between these states are key to the photonic response of optoelectronic synaptic devices. The charge state of oxygen vacancies is influenced not only by photon energy but also by the local environment and the energy levels of neighboring atoms [79]. External stimuli, such as light, can induce the transition between charged and neutral states of ${V}_{o}$, modulating the material's conductivity and electronic behavior [80]. Additionally, the concentration of charged ${V}_{o}$ at the interface determines the width of the interfacial barrier, which directly affects carrier transport [81]. Figure 3e illustrates the mechanism of oxygen vacancy ionization and deionization.

Under light illumination, oxygen vacancies absorb photons of specific wavelengths, leading to their ionization. As Yu et al*.* [22] have demonstrated that when the incident photon energy exceeds the bandgap of the semiconductor, oxygen vacancies transition from the neutral ground state to the metastable ionized state. In PPC, oxygen vacancy ionization dominates, generating free electrons (Eq. (13)), increasing the material’s conductivity, and triggering the PPC effect. Simultaneously, photogenerated holes may recombine with weakly bound oxygen (${O}_{\mathrm{wb}}^{2-}$), forming ${V}_{O}^{2+}$ and electrons, facilitating the generation of EPSC (Eq. (14)):

13 $${V}_{O}\to {V}_{O}^{2+}+2{e}^{-}$$

14 $${V}_{O}+{O}_{\mathrm{wb}}^{2-}+2{h}^{+}\to {{V}_{O}^{2+}}\,+{{O}_{i}}+2{e}^{-}$$

After the light stimulus is removed, oxygen vacancies gradually return to the ground state via a slow relaxation process, constrained by the energy barrier (${E}_{a}$) [7]. This delayed deionization leads to persistent photoconductivity in oxide semiconductors. Li et al*.* [28] demonstrated that long-wavelength light can stimulate the ZnO/PbS heterostructure, causing photon-induced excitation of oxygen vacancies, releasing free electrons, and significantly enhancing the material’s conductivity and synaptic excitatory behavior. This tunability makes it a crucial mechanism for simulating the transition from STM to LTM in biological synapses.

Conversely, deionization occurs when oxygen vacancies re-capture free electrons, reducing the material’s conductivity and generating an IPSC, as shown in Eq. (15):

15 $${V}_{O}^{2+}+2{e}^{-}\to {V}_{O}$$

In this process, photoconductivity gradually decreases due to the deionization of oxygen vacancies, manifesting as NPC under light pulses. Shrivastava et al*.* [81] demonstrated that the ZnO/Zn_2_SnO_4_-based synaptic memristor can realize all-optical controlled synaptic plasticity. Irradiation with 405 nm violet light induces the photo-ionization of interfacial oxygen vacancies, which narrows the potential barrier width, increases the PSC and thus achieves PPC. Subsequent irradiation with 633 nm red light dominates the deionization process of oxygen vacancies, broadens the potential barrier, reduces the PSC and realizes NPC.

The bidirectional photoresponse mechanism proposed by Han et al*.* [82] in ZnO/CsPbBr_3_ heterojunctions is primarily governed by the ionization and deionization processes of oxygen vacancies in the ZnO channel, which enable the switching between PPC and NPC. This process is jointly regulated by the illumination intensity and the applied bias voltage. At a bias voltage of 2 V, the device exhibits reversible transitions between PPC and NPC through modulation of the light intensity under ultraviolet and green light illumination. Under weak illumination, the number of photogenerated carriers is limited, and the deionization of oxygen vacancies dominates, leading to negative photoconductivity. In contrast, under strong illumination, a substantial increase in photogenerated carriers promotes the ionization of oxygen vacancies, resulting in a transition to positive photoconductivity. Notably, ultraviolet illumination can simultaneously excite both ZnO and CsPbBr_3_, thereby facilitating the PPC process. The threshold light intensity required to trigger PPC is > 0.386 mW cm^−2^ for ultraviolet light and > 8 mW cm^−2^ for green light illumination.

The ionization and deionization mechanisms of oxygen vacancies offer unique tunability for AOC synaptic devices, enabling the simulation of excitatory and inhibitory synaptic behaviors through light pulses of varying wavelengths. In these processes, oxygen vacancies play a central role in carrier generation and modulation, driving the “excitation–inhibition” functionality found in biological neural networks.

### Ferroelectric Polarization

Ferroelectric materials, owing to their inherent polarization properties, have become increasingly crucial in modulating photogenerated carriers in AOC synaptic devices. These materials, such as two-dimensional ferroelectric semiconductors and ferroelectric dielectrics, offer non-volatile storage with strong resistance to external electromagnetic interference, ensuring high-speed information processing and long-term stability [83]. This makes ferroelectrics particularly advantageous in neuromorphic computing applications. The mechanisms through which ferroelectric materials facilitate bidirectional synaptic functionality are multifaceted, with the polarization state of the ferroelectric layer being adjustable by external electric fields. This bidirectional control over the polarization state allows synaptic devices to dynamically switch between PPC and NPC, making ferroelectric polarization a key component for dynamic modulation of synaptic weights under optical stimuli.

For two-dimensional ferroelectric semiconductor materials, their intrinsic ferroelectric properties provide a pathway for optically stimulated modulation of both potentiation and depression under the same input wavelength [84]. The polarization state of ferroelectric materials is used to control the barrier height at semiconductor junctions. Chen et al*.* [33] explored this mechanism further in α-In_2_Se_3_, demonstrating that the gate-tunable barrier heights, induced by polarization switching, significantly affect the injection of carriers. At the *P*_up_ state, electron accumulation at the semiconductor–dielectric interface increases the tunneling width at the semiconductor-source junction, thereby reducing carrier injection and leading to NPC. Conversely, in the *P*_down_ state, the reduction in tunneling width enhances carrier injection, resulting in PPC. These effects are essential for simulating excitatory and inhibitory responses, and critical for replicating biological synaptic behavior.

In addition to being used as active layers, ferroelectric materials can also function as dielectric layers (Fig. 3f). When employed as a dielectric layer, the polarization effect becomes critical in controlling the decay behavior of photocurrent triggered by light pulses, as demonstrated by Ji et al*.* [85]. Using a CuPc/P(VDF-TrFE) structure, they fabricated a two-terminal organic synaptic device with fully light-modulated synaptic weights. P(VDF-TrFE), acting as the ferroelectric dielectric layer, induces an energy barrier at the interface due to the strong binding energy of valence electrons, which inhibits the splitting of nonequilibrium carriers toward the electrodes, leading to a pronounced photogate effect. The polarization state significantly influences the EPSC under 660 nm light pulses: at the *P*_down_ state, the increased interfacial barrier hinders carrier separation, resulting in stronger EPSC, whereas at the *P*_up_ state, negative polarization charges at the interface accelerate carrier separation, leading to a weaker photoconductive effect and shorter EPSC. Under 445 nm light pulses, the weak absorption of energy from the light excites electrons from trap states to higher energy bands, which reasonably explains the observed NPC. Whether as active layers or dielectric layers, ferroelectric polarization provides a versatile means of modulating both potentiation and depression under fully optical stimuli, playing a pivotal role in the realization of all-optical synaptic devices.

## Materials

The key problem in developing AOC artificial synapses is to construct functional materials that can generate excitatory and inhibitory synapses under the action of light. Consequently, achieving wavelength-dependent bidirectional photonic responses and synaptic plasticity within a single AOC device presents substantial challenges in material engineering. The materials employed in AOC synapses are typically complex and multifunctional. Significant advancements in the application of metal oxides, low-dimensional materials, perovskites, and organic materials have been made within the AOC synaptic devices. These materials typically serve as functional layers of AOC synapses, including dielectric and semiconductor layers. The function of two-terminal AOC synapses is always affected by the dielectric layer, and both the dielectric layer and the semiconductor layer always affect the three-terminal AOC synaptic devices. The subsequent section will briefly explore the diverse materials and strategic approaches crucial for facilitating the desired AOC synaptic functionalities.

### Metal Oxides

Metal oxide semiconductors have long been recognized as one of the most compelling materials in the development of artificial synapses, attracting extensive research efforts [86, 87]. The synaptic properties of these devices predominantly arise from the persistent photoconductivity commonly observed in oxide semiconductors, along with associated relaxation processes. The high carrier mobility, exceptional transparency, and photo-ionization of oxygen vacancies underscore the feasibility of metal oxide materials as functional layers in synaptic applications. This combination of properties suggests their substantial potential in advancing the field of neuromorphic engineering.

#### Binary Oxides

Binary oxides are increasingly explored for their stability and ease of fabrication, rendering them suitable for the scalable production of synaptic devices [88–91]. Among these, binary metal oxides with wide band gaps exhibit a confluence of advantageous properties, including high conductivity, ease of n-type doping, unique electronic structures, and visible light transparency, all of which are critical for high-performance AOC synaptic devices [90]. ZnO exemplifies these binary oxides and is notably distinguished by its capabilities to mimic synaptic functions [88, 89]. The unique attributes of ZnO, particularly in simulating synaptic functionalities, underscore its potential in advancing neuromorphic technology applications.

**Fig. 4:**
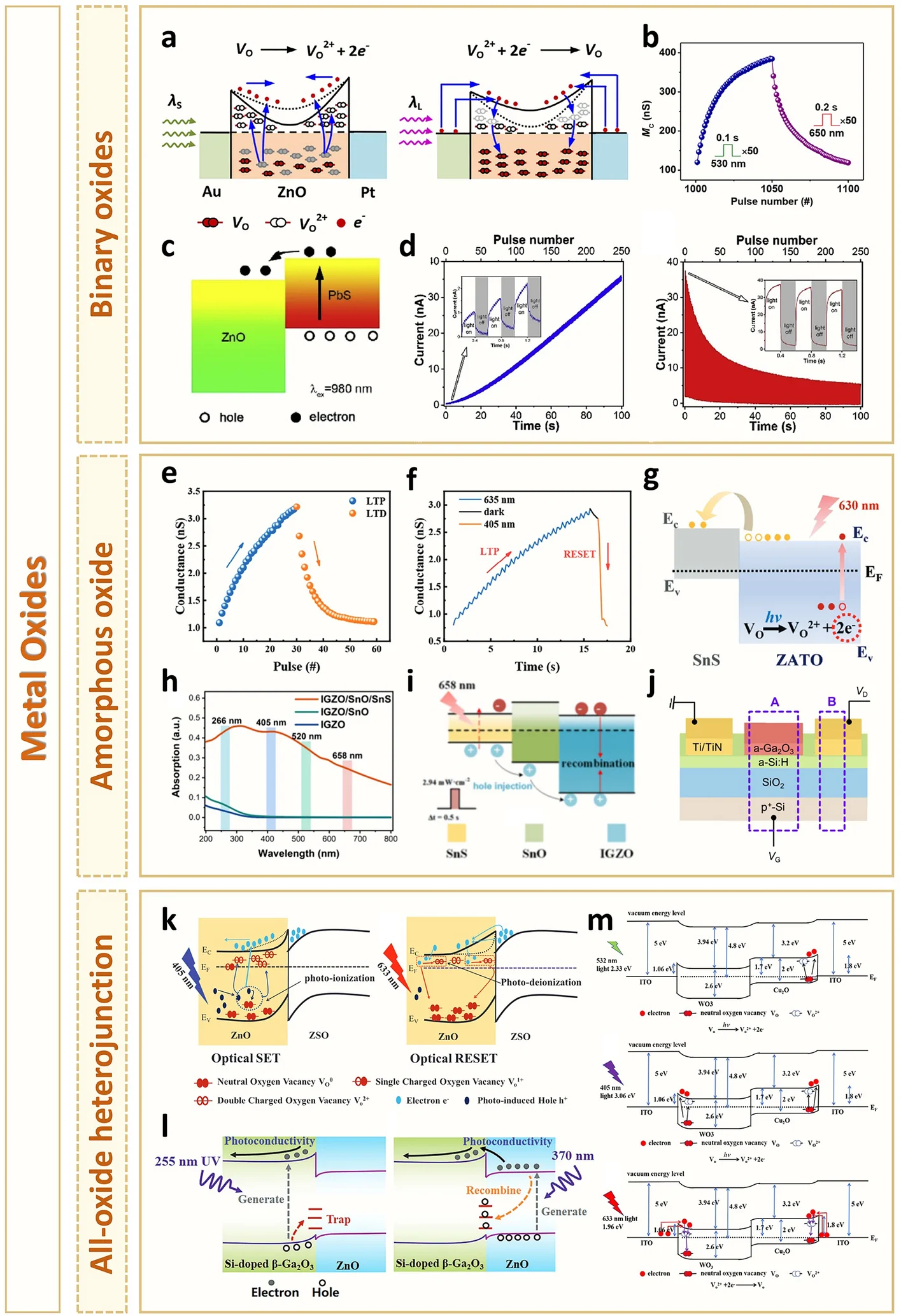
AOC synaptic device based on metal oxides. **a** Optical SET and RESET behaviors of AOC memristor based on Au/ZnO/Pt. **b** Reversible modulation of memconductance by using 50 green light pulses and 50 red light pulses Copyright 2022, The Authors. Publishing Services by Elsevier B.V. on behalf of KeAi Communications Co. Ltd [92]. **c** Schematic illustration of electron excitation and transfer processes of ZnO/PbS heterostructure at 980 nm IR light. **d** Emulated EPSC and IPSC plasticity using UV and IR light pulses. Copyright 2019, Elsevier Ltd [28]. **e** LTP/LTD process of amorphous ZnAlSnO/SnO heterojunction. **f** LTP/RESET process of amorphous ZnAlSnO/SnO heterojunction. Copyright 2023, Wiley–VCH GmbH [37]. **g** Mechanism of IPSC phenomenon in amorphous ZnAlSnO/SnS heterojunction. Copyright 2024 Wiley‐VCH GmbH [105]. **h** UV–vis absorption spectra of IGZO, IGZO/SnO and IGZO/SnO/SnS films. **i** IPSC phenomenon mechanism of IGZO/SnO/SnS synaptic device under 658 nm illumination. Copyright 2024, American Chemical Society [108]. **j** Device structure of a-Si:H/a-Ga_2_O_3_ synaptic phototransistor. Copyright 2024, Tsinghua University Press [66]. **k** Photo-ionization and deionization of oxygen vacancies in ZnO/Zn_2_SnO_4_ heterojunctions. Copyright 2023, The Authors. Advanced Electronic Materials published by Wiley–VCH GmbH [81]. **l** Schematic diagram of an energy band in the Si-doped β-Ga_2_O_3_/ZnO device under 255 and 370 nm UV illumination. Copyright 2024, Wiley–VCH GmbH [110]. **m** Working mechanism of ITO/Cu_2_O/WO_3_/ITO synaptic devices under 532, 405, and 633 nm light. Copyright 2024, The Authors. Published by American Chemical Society [109]

Yang et al*.* [92] have demonstrated AOC memristive properties using a monolayer of ZnO thin film structured in an Au/ZnO/Pt sandwich configuration, which constitutes a metal/oxide/metal junction. This structure facilitates the injection of electrons from the metal into the oxide under illumination through internal photoelectric effects or photon-assisted tunneling. By altering only the wavelength of the controlling light, the device conductance can be reversibly adjusted across a continuous range, exhibiting a persistent photoconductivity. The dominant mechanism behind the memristive effect in ZnO-based AOC synaptic devices is the reversible curvature change of the ZnO conduction band, caused by the capture and release of electrons at oxygen vacancies, as illustrated in Fig. 4a. Under ultraviolet light, oxygen vacancies transition from a neutral to a charged state, increasing the band curvature and thereby enhancing the film’s conductivity for the SET operation. Conversely, under longer wavelength light, electrons from the metal electrode enter the ZnO conduction band and partially neutralize ${V}_{O}^{2+}$. As the ${V}_{O}^{2+}$ concentration decreases, the curvature of the ZnO conduction band diminishes, facilitating an optical RESET. Figure 4b demonstrates the reversible modulation of memristive conductance under green and red light. This capability paves the way for non-volatile neuromorphic computing and the execution of complete Boolean logic functions using this AOC memristor.

The strong response of ZnO to short-wavelength light can be effectively complemented by materials that absorb at longer wavelengths. The disparity in absorption spectra minimizes optical interference, which is crucial for the full modulation of photonic synapses. PbS quantum dots, known for their excellent long-wavelength light absorption and capability for infrared encoding, represent an innovative addition to memory device applications [93–96]. Leveraging the wide band gap characteristics of ZnO and the light absorption properties of PbS quantum dots, Li et al*.* [28] have developed an AOC device based on this combination. Under photonic stimulation, ZnO provides ionization of oxygen vacancies under ultraviolet light to enhance excitatory synaptic behavior, while infrared light induces charge transfer at the hybrid heterojunction interface with PbS, modulating the state of the oxygen vacancies and thus facilitating inhibitory synapses, as shown in Fig. 4c, d. This optical scheme effectively simulates synaptic plasticity, heralding a new era in neuromorphic device applications.

#### Amorphous Oxide Semiconductors

Amorphous oxide semiconductors (AOS) such as InGaZnO (IGZO) and ZnAlSnO (ZATO) have garnered considerable interest in the research of AOC memristive and synaptic devices due to their unique properties [97–102]. Unlike their crystalline counterparts, the structural disorder inherent to amorphous materials imparts several advantages. These materials exhibit high electron mobility, superior photoelectric performance, and an extensive spectral response range, making them particularly effective for neuromorphic applications [101]. Notably, according to recent literature, the mechanism of light-induced oxygen vacancies in AOS materials plays a crucial role in achieving all-optical modulation [103]. This highlights the significant potential of AOS materials to revolutionize optical computing and synaptic device technology.

In 2017, Lee et al*.* [7] developed the *a*-IGZO UV photonic synaptic devices, proposing a working mechanism based on the ionization and deionization of oxygen vacancies. This mechanism involves a light-induced regulation of oxygen vacancies, which leads to an increase in device conductivity under light exposure and a gradual decrease when the light is removed, a phenomenon known as persistent photoconductivity. This effect renders *a*-IGZO an ideal candidate for AOC synaptic devices due to its ability to maintain conductivity changes over time. The operational mechanism of AOS materials based on the ionization and deionization of oxygen vacancies provides a novel perspective for material modification in the pursuit of advanced AOC synaptic functionalities. To amplify the role of oxygen vacancies, Hu et al*.* [34] have pioneered the development of an AOC memristor utilizing oxygen-deficient IGZO (O_D_-IGZO)/oxygen-rich IGZO (O_R_-IGZO) heterojunction. This approach involves carefully controlling the atmospheric conditions during the magnetron sputtering process to regulate the oxygen vacancy concentration in IGZO. The density of ionized oxygen vacancies (${V}_{O}^{2+}$) critically determines the width of the interface barrier, which plays a vital role in the switching behavior of the AOC memristor [76]. In this heterostructure, electrons in O_D_-IGZO tend to diffuse into O_R_-IGZO, leading to the formation of a barrier on the O_D_-IGZO side and a potential well on the O_R_-IGZO side. The SET operation is attributed to the increased concentration of ${V}_{O}^{2+}$ resulting from their ionization, which reduces the barrier width, allowing electrons to tunnel through the junction. Conversely, the neutralization of ${V}_{O}^{2+}$ leads to an increase in barrier width, reducing the tunneling current, thereby effecting the RESET process. This innovative strategy not only enhances the understanding of oxygen vacancies in IGZO-based materials but also highlights the potential of tailoring such mechanisms to optimize synaptic performance in neuromorphic devices.

More than that, to achieve bidirectional photonic synaptic behavior, AOS materials are often integrated with other materials to construct heterostructures, thereby facilitating the simulation of neural plasticity [104]. This approach has significantly advanced the field of neuromorphic computing, highlighting the potential of AOS in complex synaptic architectures. Amorphous ZATO, a representative of AOS, has demonstrated its capability to mimic complex synaptic behaviors. As a ZnO-based AOS, ZATO is an n-type wide bandgap semiconductor, where the strong bond between Al and O acts as a carrier suppressant. The inherent defects in ZATO, such as oxygen vacancies, allow for flexible modulation of its conductivity, displaying typical synaptic functions including STP, LTP, and bidirectional conductance modulation. To achieve full photonic modulation of conductance, it is essential to integrate n-type ZATO with p-type semiconductors. Common p-type materials include SnO, SnS, CuO, and Cu_2_O. Yang et al*.* have demonstrated the feasibility of ZATO/SnO [37] and ZATO/SnS [105] heterojunctions in AOC synaptic devices, with varying synaptic performances influenced by the p-type layer. In the ZATO/SnO heterojunction, the SnO layer facilitates light-induced IPSC. Under green light exposure, electron transfer from ZATO to SnO establishes a persistent low-current state, indicative of LTD, as illustrated in Fig. 4e**.** Conversely, under blue light, the faster electron mobility quickly resets the current state to baseline, demonstrating rapid RESET behavior (Fig. 4f). Differently, SnS, with a narrower bandgap of approximately 1.97 eV, responds to a broader spectrum and is more sensitive to red light (630 nm). The narrow bandgap properties of SnS under 630 nm irradiation facilitate longer wavelength light absorption, promoting rapid recombination of holes and electrons in SnS, which is illustrated in Fig. 4g, showing an IPSC response.

Amorphous IGZO is well recognized for its excellent performance in synaptic applications, commonly utilized in developing UV-responsive phototransistors [106, 107]. To extend absorption of IGZO into the visible spectrum, Zhang et al*.* [108] built upon previous works, employing p-type SnS with a high absorption coefficient as the light-absorbing layer and p-type SnO as a barrier layer within a heterostructure. They fabricated an IGZO/SnO/SnS synaptic device and investigated the specific role of SnO in bandgap engineering. Figure 4h illustrates the absorption spectra of IGZO, IGZO/SnO, and IGZO/SnO/SnS films, showing significant absorption enhancement in both the UV and visible light spectra, with SnS serving as the primary light-absorbing layer. Importantly, SnO plays a crucial role in facilitating AOC synaptic behavior. The band diagram in Fig. 4i reveals that SnO effectively reduces the valence band barrier between IGZO and SnS, thereby enhancing the efficiency of photogenerated hole injection into SnS, promoting recombination with electrons in IGZO, and achieving NPC.

In addition to the AOS materials mentioned above, Yoon et al*.* [66] have pioneered the development of a novel amorphous photonic synaptic transistor using a-Si/a-Ga_2_O_3_, as demonstrated in Fig. 4j. This device uniquely leverages the interface between a-Si/a-Ga_2_O_3_, along with a light-gated effect, to modulate synaptic behavior in response to the wavelength of incident light. The research highlights that the dominant mechanism of AOC synaptic function in amorphous systems is governed by the carrier type with higher concentration, higher capture rates, and longer trap lifetimes. Additionally, as an AOS material, this device benefits from large-area, low-temperature deposition techniques, suggesting its potential for integration with commercial CMOS processes. This advancement not only enhances our understanding of amorphous semiconductor systems but also opens up new avenues for the practical application of photonic synapses in neuromorphic computing.

#### All-Oxide Heterojunction

Multilayer structures represent a versatile strategy for integrating multiple oxide materials, combining their unique properties to enable functionalities unattainable with single-layer structures. Therefore, stacked oxide layers utilize wavelength selectivity or Schottky barrier interface effects in different layers [109] to achieve broader spectral response and tunable band alignment for bidirectional optical modulation [81, 108–110]. These designs allow precise control of conductivity and optical response, making metal oxide semiconductors and their heterostructures a diverse design platform for AOC synaptic device material selection.

In 2023, Shrivastava et al*.* [81] innovatively utilized two wide bandgap oxides, ZnO and Zn_2_SnO_4_ (ZSO), to engineer a dual-layer n-ZnO/n-ZSO structure for visible light-stimulated memristive synaptic devices. The switching mechanism in these devices is predicated on the photo-ionization and recombination of oxygen vacancies within the ZnO/ZSO heterostructure. Contact between ZnO and ZSO forms a Type II heterojunction, generating an intrinsic electric field at the interface, which creates a potential well on the ZSO side and a potential barrier on the ZnO side, as depicted in Fig. 4k. This device exploits the ${V}_{O}^{2+}$ concentration at the interface barrier to exhibit memristive switching behavior. Under illumination with violet light (405 nm) and red light (633 nm), the device facilitates LTP and LTD operations. These features have successfully simulated the classical Pavlovian conditioning experiment, demonstrating promising applications in optical neuromorphic computing and human–machine interfaces.

Moreover, Sun et al*.* [110] have explored the capabilities of a Si-doped β-Ga_2_O_3_/ZnO heterostructure, demonstrating advanced UV photonic synaptic functionalities modulated across two distinct UV wavelengths (255 and 370 nm). The incorporation of silicon into β-Ga_2_O_3_ significantly enhances carrier concentrations and exhibits a persistent photoconductivity effect. As illustrated in Fig. 4l, the integration of Si-doped β-Ga_2_O_3_ with ZnO not only increases the efficiency of photogenerated carrier production but also optimizes their recombination dynamics. This synergy enables the reversible modulation of electrical conductivity, showcasing a sophisticated approach to controlling synaptic behaviors in neuromorphic devices using ultraviolet light.

Further advances in all-oxide heterostructures for synaptic devices are evident in the work by Wu and Tseng, where a complex ITO/Cu_2_O/WO_3_/ITO stack was developed [109]. Illustrated in Fig. 4m, the operational mechanisms of the ITO/Cu_2_O/WO_3_/ITO synaptic device are distinct across the light spectrum. Green light (532 nm) predominantly enhances the conductivity of Cu_2_O, while violet light (405 nm) boosts conductivity in both Cu_2_O and WO_3_. However, under red light (633 nm), the photon energy is insufficient to excite electrons in the valence bands of Cu_2_O and WO_3_. Instead, it enables electrons in ITO to overcome the Schottky barrier, reaching the conduction band where they are captured by ${V}_{O}^{2+}$, resulting in reduced optical conductivity. This layered heterostructure increases the flexibility of optical modulation, enabling the device to operate across a broader spectral range while maintaining high transparency and effective photoresponse characteristics.

In general, oxide semiconductors are pivotal materials in fabricating AOC synapses. However, their inherent wide bandgaps typically limit responsiveness to visible and near-infrared light. Integrating oxide semiconductors with narrow bandgap materials emerges as an effective strategy to broaden the spectral response range of devices. Additionally, defect engineering, elemental doping, and other methods can enhance the material’s light responsiveness. Rational device structure design, such as constructing type-II heterojunctions, further facilitates the realization of AOC synaptic functionalities.

### Low-Dimensional Materials

Low-dimensional materials, characterized by their strong light–matter interactions, tunable bandgaps, and rich defect states, have become pivotal in advancing AOC synaptic devices [111, 112]. These include zero-dimensional (0D), one-dimensional (1D), two-dimensional (2D) structures, and their heterostructures, each contributing unique properties to device functionality.

#### Zero-Dimensional (0D) Materials

Among various material classes, 0D materials exhibit distinct optical and electrical properties, making them highly suitable for AOC synaptic research. Quantum dots (QDs), representing 0D materials, are particularly valued for their exceptional quantum confinement effects and high photoluminescence efficiency [113, 114]. Through precise control over the size, shape, and surface chemistry of quantum dots, their electrical and optical characteristics can be tailored to meet specific application requirements. A notable example is the hybrid heterostructure of ZnO/PbS, which was reported by Li et al*.* [28] in 2019. This device leverages PdS QDs for a unique charge transfer mechanism, successfully simulating synaptic activities under a full photonic modulation regime.

**Fig. 5:**
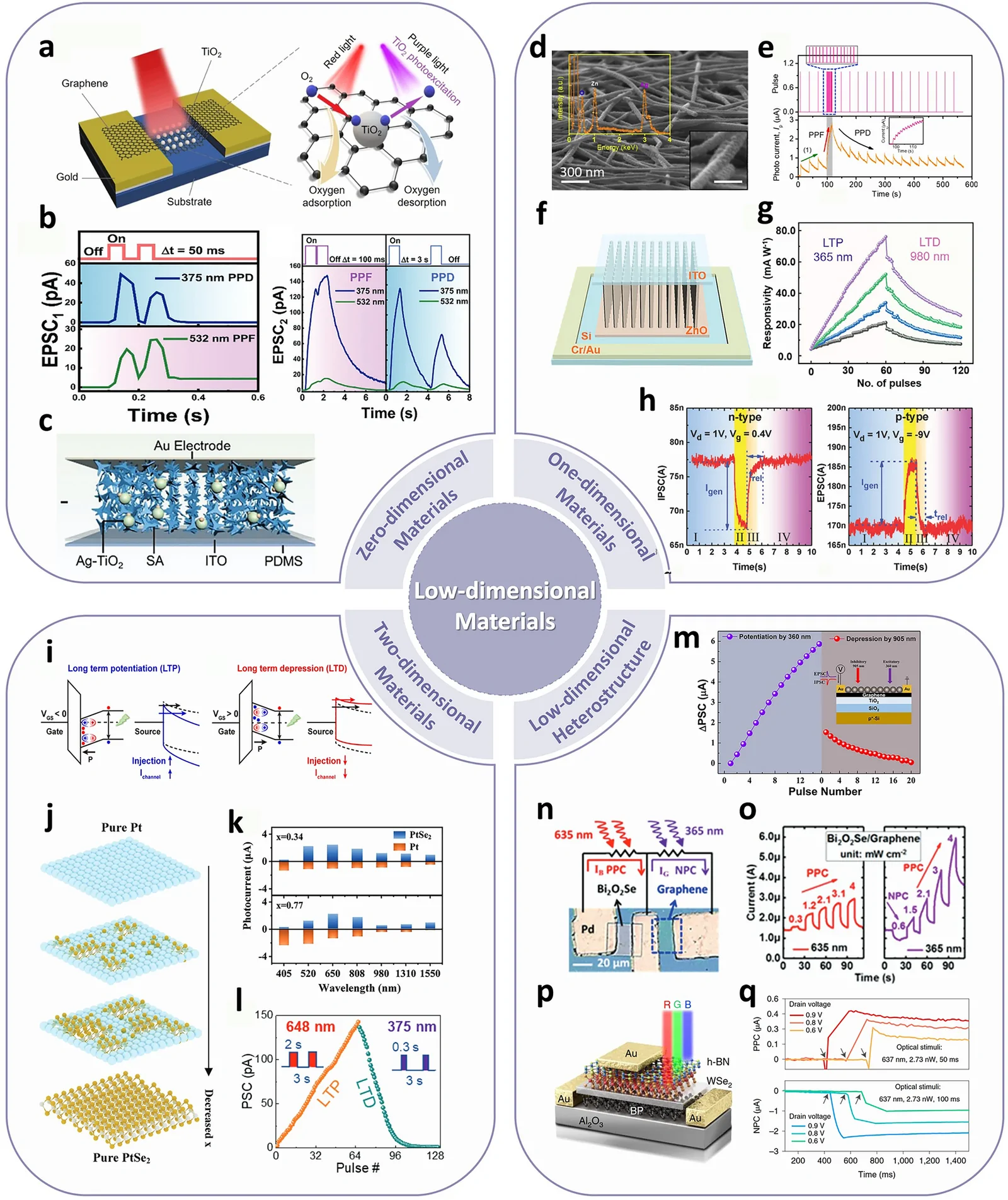
AOC synaptic device based on low-dimensional materials. **a** Schematic diagram of the Au/TiO_2_ QDs-graphene/Au artificial optical synapse Copyright 2023, American Chemical Society [72]. **b** PPF/PPD behavior of Si NCs/P3HT hybrid synaptic devices in two-terminal and three-terminal modes, respectively. Copyright 2021, Wiley–VCH GmbH [115]. **c** Structure diagram of Ag-TiO_2_ nanocluster/sodium alginate-based memristor. Copyright 2024, The Author(s). Advanced Science published by Wiley–VCH GmbH [67]. **d** SEM image tilt view of ZnO/Ag NWs/PET device. **e** UV pulse duration dependent PPF and PPD induction of ZnO/Ag NWs/PET device. Copyright 2019, Elsevier Ltd [118]. **f** Schematic diagram of p-Si/n-ZnO NWs heterojunction synaptic device. **g** LTP/LTD behavior of p-Si/n-ZnO NWs synaptic devices. Copyright 2023, Wiley‐VCH GmbH [119]. **h** Transient characteristics of n-type and p-type LPSNFETs after TPPS modification with 1 s-wide light pulse. Copyright 2021, Wiley–VCH GmbH [120]. **i** Energy band diagrams of the synaptic weight update process of LTP/D for a-In_2_Se_3_ FET. Copyright 2023, American Chemical Society [33]. **j** Schematic diagram of the transition from Pt to PtSe_2−x_ and PtSe_2_. **k** Relationship between the photocurrents of Pt and PtSe_2_ under different illuminate wavelengths with different x (x = 0.77,0.23). Copyright 2022, Wiley–VCH GmbH [32]. **l** LTP/LTD characteristics of WSe_2_/WO_x_ transistor. Copyright 2023, Wiley–VCH GmbH [133]. **m** Synaptic potentiation-depression cycle achieved by the 360 and 905 nm light pulses in PbS-Gr-TiO_2_ device. Copyright 2023, American Chemical Society [137]. **n** Structure diagram of Bi_2_O_2_Se/Gr hybrid photodetector. **o** Time-dependent current curves for the Bi_2_O_2_Se/Gr hybrid structure illuminated at 635 and 365 nm. Copyright 2020, WILEY–VCH Verlag GmbH and Co. KGaA, Weinheim [29]. **p** Structure diagram of BP/Al_2_O_3_/WSe_2_/h-BN device. **q** Essential PPC and NPC curves under drain voltage modulation. Copyright 2021, The Author(s), under exclusive licence to Springer Nature Limited [145]

QDs contribute to synaptic device functionalities beyond mere charge transfer mechanisms. Liang et al*.* [72] have ingeniously employed TiO_2_ quantum dots to modulate the conductivity of graphene layers, crafting a dual-terminal graphene/TiO_2_ QDs device that exploits photo-initiated gas adsorption and desorption effects. As depicted in Fig. 5a, TiO_2_ QDs serve as centers for oxygen adsorption and desorption. These processes are modulated by varying the wavelength of light exposure, which adjusts the quantity of oxygen molecules adsorbed on the TiO_2_ QDs. This modulation results in bidirectional conductivity changes in the graphene layer, showcasing the versatility of QDs in AOC synaptic devices. This innovative approach highlights the potential of quantum dots to enable complex, multifunctional synaptic responses through novel photochemical mechanisms.

Unlike the QDs semiconductors previously mentioned, silicon nanocrystals (Si NCs) are a common 0D material known for their broad absorption efficiency spanning from ultraviolet to near-infrared wavelengths, particularly under heavy doping. Capitalizing on this, Wang et al*.* [115] have developed a dual-working-mode synaptic device utilizing a hybrid structure of Si NCs and poly(3-hexylthiophene) (P3HT). The Si NCs predominantly absorb UV light, while P3HT primarily captures visible light, enabling broad spectral photonic absorption in the Si NCs/P3HT hybrid device. The principal mechanism of operation in this device lies in the induced potential wells at the Si NCs/P3HT heterojunction, facilitating unique performances across varying wavelengths and operational modes. In a tri-terminal working mode, this device exhibits LTD and LTP under 375 and 532 nm light stimulation, respectively, demonstrating wavelength-selective synaptic plasticity as depicted in Fig. 5b. In a dual-terminal mode, the device’s PPF and PPD behaviors are dependent on the timing interval between consecutive spike potentials, indicating frequency-dependent synaptic plasticity. The synergistic effect of these dual working modes endows the synaptic plasticity with multifunctionality.

Nanoclusters, as a subset of 0D materials, have recently been leveraged by Shan et al*.* [67] to simulate human retinal functions. They embedded Ag-TiO_2_ nanoclusters within a sodium alginate film to develop a hemispherical photonic memristor array capable of facilitating neuromorphic stereoscopic vision. Figure 5c illustrates the device structure of the Ag-TiO_2_ nanocluster/sodium alginate film. This array can reversibly achieve EPSC and IPSC when subjected to 350 nm UV and 570 nm visible light pulses, respectively. The full photonic modulation of synaptic plasticity in this device is attributed to the defect states in TiO_2_ nanoparticles and the localized surface Plasmon resonance effects of Ag nanoparticles.

Despite the broadband responsiveness of AOC synapses fabricated from 0D materials, there remains room for improvement in these devices. From a materials advancement perspective, the development of more advanced and straightforward material synthesis methods, surface modifications, material doping, and structural design are eagerly anticipated to enhance the rapid response capabilities of AOC synaptic devices.

#### One-Dimensional (1D) Materials

1D materials have nanoscale in dimension direction and macroscopic scale in length. Electrons can only move freely in straight lines in nanoscale directions. 1D materials, such as nanowires, nanotubes, and nanoribbons, are highly promising for use in artificial synaptic devices due to their unique physical properties and high surface-to-volume ratios [116, 117]. Firstly, 1D materials exhibit strong light–matter interactions due to their constrained geometry and quantum confinement effects, enhancing their optical absorption across a broad spectrum. This makes them ideal candidates for AOC devices requiring responsive behavior to various wavelengths of light. Secondly, the inherent anisotropy of 1D materials allows for directional charge transport, which is beneficial for reducing crosstalk between adjacent synaptic devices in densely packed arrays. This is more conducive to simulating precise and efficient synaptic activity. Additionally, the large surface area of 1D materials enhances their reactivity and interaction with external stimuli such as light and gases. This feature can be exploited to dynamically modulate the electronic properties of synaptic devices through surface chemistry modifications, such as doping and functionalization.

Notably, nanowires (NWs) are particularly attractive for fabricating flexible synaptic devices. Kumar et al*.* [118] utilized the piezoelectric effects and unique properties of ZnO and Ag NWs to provide a new approach for designing wearable and efficient synaptic devices. Ag NWs were spin-coated on a PET substrate, followed by the growth of a ZnO film via atomic layer deposition on Ag NWs/PET, forming a two-terminal ZnO/Ag NWs/PET AOC synapse. Figure 5d shows a tilted-view SEM image of the ZnO/Ag NWs/PET device. In this work, the generation and capture of photo-induced electron–hole pairs are central to synaptic functionality. Ag NWs provide a pathway for effective charge collection. When UV light is applied, the electrodes generated in the ZnO film are rapidly transferred to the electrodes by Ag NWs. More importantly, the strain-induced piezoelectric effect within ZnO provides an additional degree of modulation. Applying strain to ZnO not only induces polarized charges but also modulates the adsorption/desorption process of oxygen. For example, depending on the duration of the photon pulse sequence, the device can mimic the transition from PPF to PPD. As shown in Fig. 5e, under short pulse intervals, the generation of photocarriers dominates, leading to PPF, whereas, for longer pulse intervals, the rapid recombination of captured electrons results in PPD.

Zhang et al*.* [119] utilized p-Si and n-type ZnO NWs to construct a p-Si/n-ZnO NWs heterojunction synaptic device (Fig. 5f). The bandgap characteristics and electron affinity differences between the two materials create an effective heterojunction structure, which aids in the efficient separation and transport of carriers under optical excitation. Under UV and UIR light pulses, the reversible conversion between neutral and charged oxygen vacancies in ZnO NWs and the adsorption/desorption of oxygen molecules at the surface/interface effectively modulate the resistance of ZnO NWs and the size of the built-in electric field within the device, thereby achieving multi-level and bidirectional control of the photocurrent response. As shown in Fig. 5g, the p-Si/n-ZnO NWs synaptic device exhibits LTP/LTD behavior under 365 nm UV and 980 nm UIR. The use of NWs enables complex sensing and data processing tasks with reduced energy consumption and latency.

Expanding upon using 1D materials, Li et al*.* [120] introduced a CMOS-compatible light-stimulated porphyrin-coated silicon nanowire field-effect transistor (LPSNFET) technology. This innovative approach employs just two CMOS-like transistors to form complementary photo-synapses, effectively integrating the high photosensitivity of porphyrin with the ideal low-dimensional transport characteristics of silicon nanowires. By simply changing the MOS type of the LPSNFET or modulating the Si NWs/porphyrin interface, complementary inhibitory and excitatory synapses can be achieved. As demonstrated in Fig. 5h, under 435 nm blue light illumination, the drain-source current of an n-type LPSNFET rapidly decreases upon light stimulation, resembling the IPSC of biological inhibitory synapses. Conversely, the p-type LPSNFET displays EPSC behavior. Thus, the complementary enhancement and inhibition functionalities are effectively implemented in p-type and n-type devices, respectively. Moreover, compared to bare silicon NWs devices, the photosensitivity of the LPSNFET is enhanced fivefold, ensuring effective response even under low incident illumination power. This marked improvement underscores the significant advantages of integrating 1D silicon nanowires into neuromorphic devices, leveraging their enhanced light absorption and effective charge carrier mobility.

#### Two-Dimensional (2D) Materials

In contrast to 0D materials such as QDs and 1D materials like nanowires, 2D materials have garnered significant attention in recent years for their use in AOC synaptic devices. Characterized by atomic-level thickness and superior surface attributes, 2D materials are predominantly utilized in forms such as nanosheets, facilitating nanoscale precision in measuring film thickness and interface depth [121]. In two-terminal synaptic devices, the high carrier mobility and exceptional conductivity of 2D materials enable faster operational speeds, which is crucial for efficient synaptic emulation [122, 123]. For three-terminal devices, the attributes of 2D materials, including ease of solution processing and minimal thickness, substantially reduce manufacturing costs [124]. This economic advantage, combined with their functional properties, makes 2D materials ideal for scaling up neuromorphic computing technologies. In the realm of AOC synaptic devices, 2D materials such as graphene (Gr), black phosphorus (BP), hexagonal boron nitride (h-BN), molybdenum disulfide (MoS_2_), and transition metal dichalcogenides (TMDs) have made significant breakthroughs.

Graphene, one of the most celebrated two-dimensional materials, is renowned for its exceptional electronic mobility and optical transparency [125–127]. It serves as an efficient channel for rapid electron transport, facilitating swift photoelectric responses essential for high-performance synaptic emulation [128]. Moreover, graphene's remarkable mechanical strength and flexibility make it an ideal material for wearable technologies, where durability and adaptability are paramount. Consequently, graphene is frequently employed as a conductive channel material within van der Waals heterostructures, enhancing device integration and performance.

Similarly, BP, a standout two-dimensional material, is increasingly recognized for its unique advantages in photonics, notably its tunable bandgap which facilitates essential light–matter interactions. However, BP's propensity to oxidize spontaneously in ambient conditions, generating abundant defect states, alters its intrinsic photoelectric properties [129, 130]. By strategically utilizing these natural defects, researchers have created unique photoelectric functionalities. Since 2019, Ahmed et al*.* [27, 30, 68] have pioneered the study of BP-based AOC synaptic devices through defect engineering. Utilizing mechanically exfoliated BP layers, they successfully developed an artificial synaptic device capable of wavelength-selective modulation of both positive and negative photoconductance. Intriguingly, both EPSC and IPSC are induced using different wavelengths within the ultraviolet spectrum instead of electrical signals. This is facilitated by charge trap states at the interface between the naturally oxidized phosphorus (PO_x_) and BP under ambient conditions, which provide a unique photoresponse in BP-based synaptic devices across varying UV wavelengths. This work paves the way for simulating complex neural functions purely through photonic modulation, offering a promising avenue for advancements in neuromorphic computing.

Beyond graphene and BP, a new class of 2D materials based on TMDs has recently garnered significant attention, particularly due to their inherently narrow bandgaps. To date, TMDs such as MoS_2_, In_2_Se_3_, PdSe_2_, PtSe_2_, and WSe_2_, have been actively explored for their potential in revolutionizing the fields of photodetection and optoelectronic synaptic devices. These materials offer unique optoelectronic properties that are ideal for the development of next-generation devices capable of enhanced light absorption and efficient charge transport. The optical applications of MoS_2_, a widely studied 2D material, have traditionally been confined within the visible spectrum due to its bandgap width of approximately 1.2 eV [131]. To overcome this limitation and extend the material’s photodetection capabilities into the near-infrared (NIR) region, Wu et al*.* [132] have innovatively designed a MoS_2_-based FET, tu incorporating an Au/Cr/Au structure. This configuration enables the MoS_2_ FET to exhibit ultra-sensitive photoresponse at visible wavelengths and, notably, pioneering negative photoconductivity at NIR wavelengths. At 454 nm light, the device exhibits PPC phenomena, while at 980 and 1550 nm light, it exhibits fast-responding NPC. Through temperature-dependent electrical measurements, they established that while the photovoltaic effect governs the visible light response, a thermal mechanism facilitates the negative infrared photocurrent. This breakthrough not only broadens the functional range of MoS_2_ photodetectors but also enhances the understanding of the complex interplay between electronic and thermal effects in two-dimensional materials.

The emerging 2D ferroelectric semiconductor a-In_2_Se_3_, with its coupled ferroelectric and semiconductor properties and suitable photosensitive bandgap, has shown potential for adaptive synaptic applications. Under polarization switching, a-In_2_Se_3_ enables bidirectional modulation of synaptic weights upon optical pulse stimulation. Chen et al*.* [33] reported a light-activated bidirectional synaptic phototransistor based on this ferroelectric semiconductor a-In_2_Se_3_. Unlike conventional bidirectional synaptic devices that rely on defects or charge trapping/de-trapping mechanisms, the operation of a-In_2_Se_3_ phototransistors hinges on polarization-induced gate barrier height modulation. As demonstrated in Fig. 5i, in the P_down state, the reduced tunneling width at the semiconductor–dielectric interface facilitates carrier injection, thereby increasing channel conductance. Conversely, in the P_up state, the interfacial barrier height increases, reducing conductance. By applying optical pulses under these distinct polarization-controlled states (P_down and P_up), the a-In_2_Se_3_ synaptic device achieves LTP and LTD characteristics.

Moreover, TMDs are renowned for their elemental tunability, offering a pathway for material modification and functionality enhancement. For example, Jiang et al*.* [75] constructed an AOC synaptic device based on the intrinsic defect sensitivity and superior photoresponsiveness of PdSe_2_, and verified with DFT calculations that its working mechanism stems from the wavelength-dependent desorption of oxygen clusters and intrinsic Se vacancy defects. Fascinatingly, adjusting the elemental ratios in TMDs can precipitate a transition from semiconducting to semimetallic properties. Lian et al*.* [32] have explored this potential through selenization engineering to manipulate the composition of PtSe_2-x_ films, achieving bidirectional photonic responses. During the selenization process, a pure Pt film undergoes a structural transformation to form PtSe_2-x_, as depicted in Fig. 5j. As the PtSe_2_ component increases, the direction of the photocurrent in the hybrid device shifts from NPC to PPC across a broad spectral range of 405–5000 nm. Illustrated in Fig. 5k, the photocurrent of PtSe_2-x_ is calculated as the difference in absolute photocurrents between pure Pt and PtSe_2_. The pure photocurrent of Pt decreases with increasing wavelength, while that of PtSe_2_ peaks at 520 nm. At a wavelength of 405 nm, the thermoelectric effect of metallic Pt dominates, leading to NPC behavior; however, as the wavelength extends to 520 nm, the positive photoconductivity effect of PtSe_2_ surpasses the thermal effects, causing a shift to PPC.

In addition to controlling the proportion of elements, the surface of the material can also be treated. Wu et al*.* [133] developed a new strategy based on partial oxidation of WSe_2_ surfaces. After oxidation, Se^+^ and O^2−^ defective states are formed close to the conduction and valence band edges, acting as electron and hole traps, respectively. The device exhibits bidirectional synaptic plasticity of excitation and inhibition, which are fully controlled by visible and UV light stimulation, respectively. As depicted in Fig. 5l, the WSe_2_/WO_x_ transistor exhibits LTP and LTD characteristics, mimicking the functionality of a tetrachromatic vision system. Advances in surface treatment technology have enhanced the photon response and synaptic behavior of TMDs-based devices.

#### Low-Dimensional Heterostructure

To achieve superior artificial synaptic characteristics, researchers are increasingly exploring the use of various low-dimensional materials in heterostructure configurations within single AOC devices. These devices, crafted from mixed low-dimensional materials, exhibit outstanding or enhanced synaptic functionalities [134], making low-dimensional heterostructures an attractive option in the field of artificial synapses and neuromorphic vision. Currently, the most widely implemented low-dimensional heterostructure AOC synaptic devices fall into two primary categories: 0D/2D heterostructures and 2D/2D heterostructures. These devices predominantly operate based on the principles of charge capture and release at the interface between two-dimensional materials and their substrates or adsorbed surfaces. This architectural strategy not only utilizes the intrinsic properties of each material but also leverages the synergistic effects at their interfaces to significantly boost the performance of synaptic devices.

In the 0D/2D system, 0D materials, characterized by their quantum confinement effects, act as efficient photosensitizers, exhibiting exceptional optical properties. Meanwhile, 2D materials, noted for their lack of dangling bonds and weak interlayer interactions, serve as effective charge transfer layers. Under external light stimuli, the synergy between the quantum-confined 0D materials and the charge transport capabilities of the 2D layers leads to enhanced performance in photonic synaptic devices [135, 136]. Therefore, 2D/QDs heterojunction has significantly advanced the development of three-terminal AOC synaptic devices, addressing a crucial gap in this research field. Wen et al*.* [137] introduced an innovative PbS-Gr-TiO_2_ device that integrates wide bandgap polycrystalline TiO_2_ and narrow bandgap PbS QDs into a graphene transistor, enabling light gate effects with near-UV and near-IR light responsiveness. Comparative analyses with pure graphene transistors, Gr-TiO_2_, and PbS-Gr devices demonstrate that the photonic responses of the PbS-Gr-TiO_2_ device predominantly originate from TiO_2_ and PbS QDs. Specifically, PbS QDs, serving as an electron capture matrix, predominantly respond to near-IR, while TiO_2_, acting as a hole capture matrix, predominantly responds to UV light. The differing charge capture polarities of these absorbers confer the PbS-Gr-TiO_2_ device with bidirectional photonic responses dependent on the incident light wavelength. As shown in Fig. 5m, the device exhibits PPC under 360 nm and NPC under 905 nm, effectively modulating synaptic enhancement and suppression behaviors.

In particular, the integration of 2D materials and QDs within simple, low-dimensional heterostructures significantly enhances the flexibility of optical synapses, making them well suited for wearable electronics. Graphene, known for its outstanding electrical conductivity and flexibility, has been pivotal in developing responsive synaptic architectures. In a previous report, a bipartite optical synapse based on a vertical heterostructure of wafer-scale pyrenyl graphdiyne (Pyr-GDY)/graphene/PbS QDs was used for large-scale flexible devices [24]. Within this device, Pyr-GDY and PbS QDs function as photonic charge capture layers that modulate inhibitory and excitatory synaptic behaviors, respectively. Graphene serves as a conduction channel, where its conductivity is linearly modulated by the charge carriers, holes in Pyr-GDY and electrons in PbS quantum dots. The Pyr-GDY/Graphene/PbS-QDs synaptic device offers a method for fully photonic modulation of synaptic weights, simplifying the structure and circuitry of synaptic transistors. This innovation demonstrates the potential of low-dimensional materials for achieving flexible synapses in wearable applications, thereby opening new avenues for the integration of advanced materials in neuromorphic computing. The strategic placement of graphene allows it to harness the unique electronic properties of adjacent materials, thereby enhancing the device's overall responsiveness and stability. This synergy not only exemplifies the potential of low-dimensional materials in complex synaptic systems but also sets a new benchmark for the design of next-generation photonic synaptic devices.

In the domain of 2D material systems, the van der Waals effect mitigates the constraints of lattice matching, permitting the stacking of disparate materials [138]. This flexibility enriches the operational and physical properties of devices. Particularly, 2D/2D heterostructures utilize strategic band alignment to facilitate photostimulated synaptic plasticity across a broad spectral range. The emerging layered material, bismuth oxychalcogenide (Bi_2_O_2_Se), notable for its high electron mobility and broadband optical response [139, 140], has been identified as a promising candidate for AOC applications. Yang et al*.* [29] have pioneered the integration of Bi_2_O_2_Se with graphene in a hybrid structure, capitalizing on the unique photoelectronic properties of the 2D/2D system. This hybrid device exhibits controlled positive or negative photoresponse depending on the stimulated wavelength, successfully implementing synaptic functionalities such as LTP and LTD. The structure of the Bi_2_O_2_Se/Gr hybrid photodetector is illustrated in Fig. 5n. Under illumination at wavelengths of 635 and 365 nm, the effects of light on the Bi_2_O_2_Se and graphene channels were studied to differentiate between NPC and PPC in the hybrid device. As shown in Fig. 5o, at 635 nm, the Bi_2_O_2_Se/Gr device exhibits an increase in current within the intensity range of 0.3–4 mW cm^−2^, displaying PPC behavior. Conversely, a transition from NPC to PPC is observed when the light source wavelength shifts from 635 to 365 nm. Experiments demonstrate that at 0.6 mW cm^−2^, the occurrence of NPC is predominantly governed by the negative photoresponse of graphene. However, as the illumination intensity increases, the thermal effects of Bi_2_O_2_Se intensify, overcoming graphene’s NPC and reestablishing PPC behavior. This behavior highlights the mechanism of this work, namely that the formation of NPC or PPC is controlled by the intensity of UV exposure.

2D materials are exceptionally responsive to external light stimuli, closely mirroring the functional capabilities of the human retina by perceiving light information and converting it into electrical signals for transmission and processing. This sensitivity positions 2D materials as prime candidates for the next-generation of visual sensors, expanding beyond traditional memory oxides to include 2D materials and their heterostructures [141–144]. A notable advancement in this domain is the development of a 2D retinal device composed of a BP/Al_2_O_3_/WSe_2_/h-BN heterostructure, which exhibits non-volatile bidirectional photoconductivity characteristics (Fig. 5p) [145]. Figure 5q illustrates the device’s PPC and NPC curves under two adjustable working states, corresponding to the functionalities of ON/OFF bipolar cells and retinal neurons, respectively. Under drain voltage modulation, a laser at 637 nm with an intensity of 2.73 nW generates PPC and NPC at durations of 50 and 100 ms, respectively. This showcases the robust integration potential of 2D materials, allowing for reconfigurable switching states that enable applications such as motion detection and edge detection. Such capabilities significantly advance the bionic design applications of human eye mimetics, offering a multitude of possibilities for enhancing sensory experiences and computational efficiency in artificial vision systems.

In general, the fusion of various low-dimensional materials to create hybrid nanostructures presents a promising strategy to simplify and efficiently detect information while precisely emulating the neural characteristics of photonic synapses at the nanoscale. These structures harness the unique properties of each material to provide enhanced sensitivity and fidelity in simulating neural processes, potentially revolutionizing neuromorphic computing. However, realizing their practical application demands substantial improvements in device performance. To transition these technologies from the laboratory to real-world applications, the development of innovative methodologies and designs is imperative. Advancing beyond current capabilities will require not only novel engineering approaches but also a deeper understanding of material interactions within these complex systems.

### Perovskite

Perovskite materials are increasingly recognized in the field of photonic synaptic devices due to their high carrier mobility, tunable bandgap, broad absorption spectrum, and intrinsic defects [146–148]. These properties make perovskites highly suitable for dual roles in AOC devices, serving both as floating gate layers and photosensitive layers. The multifunctional nature of perovskites supports complex photonic operations, which are essential for advanced neuromorphic computing systems. Key mechanisms driving perovskite functionality include carrier migration at heterojunction interfaces, depletion layer formation, ionization and recombination of defect states, and photo-induced ion migration between perovskites and electrodes [149–155]. Each mechanism contributes to the dynamic modulation of conductivity and synaptic behavior, enabling real-time processing of optical signals. These insights open the door to optimizing perovskite compositions and device architectures to harness these mechanisms for improved performance.

**Fig. 6:**
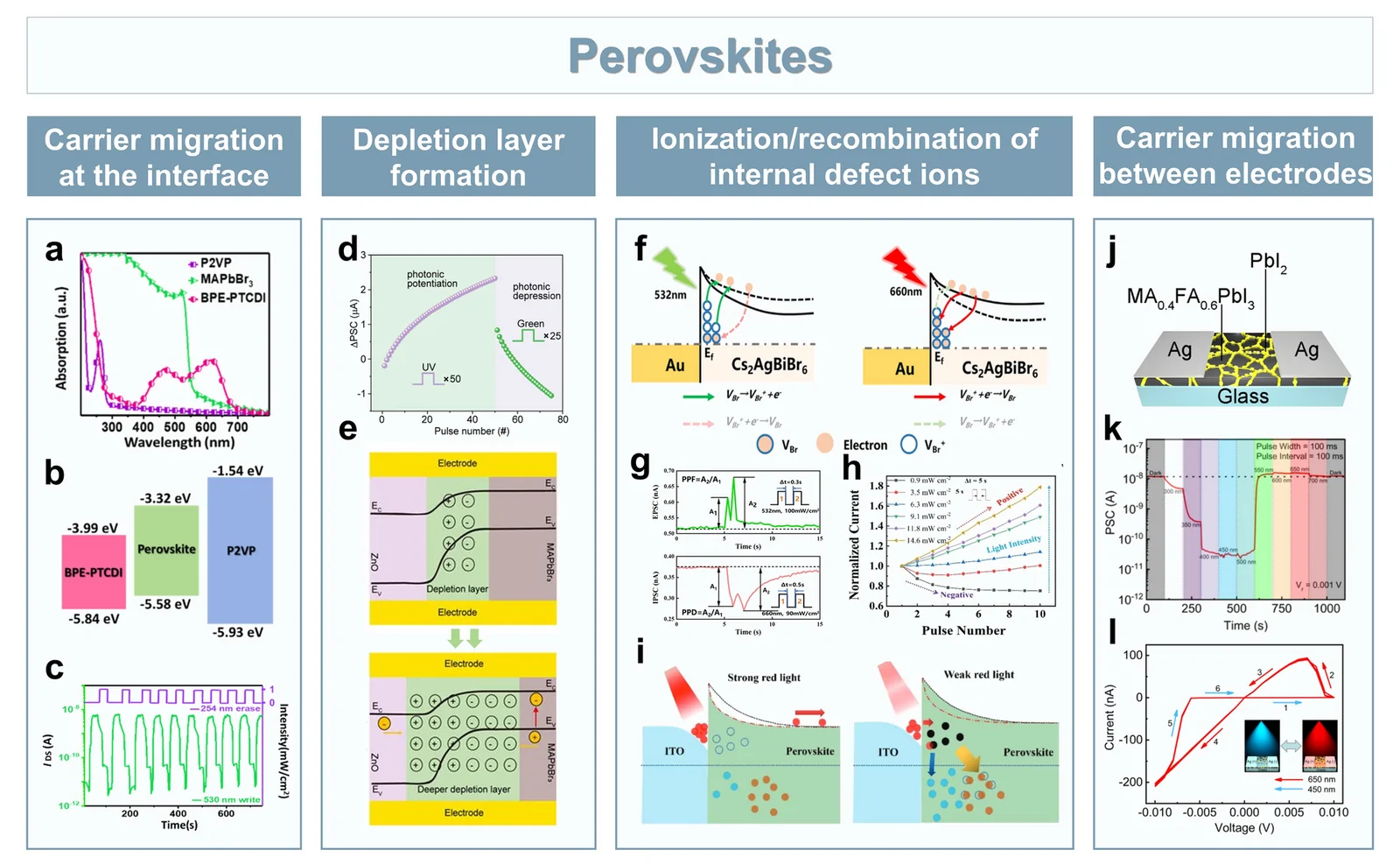
AOC synaptic device based on perovskites. **a** UV − vis absorption spectra of P2VP, MAPbBr_3_, and BPE-PTCDI films. **b** Energy level diagrams of P2VP, MAPbBr_3_, and BPE-PTCDI films. **c** Photo-writing at 530 nm and photo-erasing at 254 nm Copyright 2021, American Chemical Society [149]. **d** Potentiation under UV irradiation and depression under green irradiation. **e** Schematic illustration of perovskite-ZnO heterojunction before and after green light illumination. Copyright 2022, Wiley‐VCH GmbH [150]. **f** Working mechanism of the Au/Cs_2_AgBiBr_6_/Au memristor. **g** PPF and PPD effects induced by green and red light pulses. Copyright 2023, The Royal Society of Chemistry [151]. **h** Change of positive and negative photocurrent with the intensity of light pulse. **i** Switching mechanism of the Cs_x_FA_y_MA_1-x-y_Pb(I_z_Br_1-z_)_3_-based memristor. Copyright 2023, Wiley–VCH GmbH [153]. **j** Structure of Ag:AgI/MA_0.4_FA_0.6_PbI_3_/Ag:AgI optically responsive memristor. **k** PSC at different wavelengths. **l** I-V characteristics under 450 and 650 nm light illumination. Copyright 2024, American Chemical Society [155]

In 2021, a novel dual-functional phototransistor memory based on perovskite materials was developed, capitalizing on the high photo responsiveness and carrier dynamics of perovskites to enable light-programmable and light-erasable functionalities [149]. This innovative memory device incorporates a bifunctional photoactive floating gate composed of poly(2-vinylpyridine) (P2VP) and perovskite, facilitating comprehensive light-programming capabilities. Distinct from earlier implementations [156], this device uniquely exploits the complementary light absorption between the photoactive n-type material BPE-PTCDI in the active channel and the hybrid floating gate CH_3_NH_3_PbBr_3_ (MAPbBr_3_)/P2VP, covering a wide spectral range from ultraviolet to near-infrared. As shown in Fig. 6a, MAPbBr_3_ exhibits an absorption band below 550 nm, effectively extending the material's absorption range to achieve full-spectrum coverage. Intriguingly, MAPbBr_3_ provides a suitable band alignment within the device, ensuring efficient charge transfer pathways as depicted in Fig. 6b. The advantageous energy level alignment between the floating gate and the channel layer prevents electron trapping while stabilizing captured holes. Under the synergistic effects of BPE-PTCDI and the hybrid floating gate MAPbBr_3_/P2VP, the device achieves light-writing under visible light and UV-induced light-erasing. Figure 6c demonstrates the write/erase cycles under illumination at 530 and 254 nm, respectively. The broad absorption spectrum of MAPbBr_3_ allows for light-writing at illumination levels of 650, 530, and 405 nm, while light-erasing is facilitated at 254 nm due to carriers generated by P2VP being neutralized within BPE-PTCDI and MAPbBr_3_. The extensive light absorption capabilities and matched energy levels of perovskite materials exhibit vast potential within AOC synaptic devices, pointing toward a promising future in neuromorphic applications where dynamic and reversible light interactions are essential.

The integration of perovskites with other materials into multilayer structures further expands their functionality. In general, MAPbBr_3_, as a P-type conductive material, can be assembled with n-type materials to form a typical p-n heterojunction. Among these, ZnO stands out as an exceptional electron transport layer within perovskite heterostructures, having demonstrated substantial efficacy in photonic applications [157, 158]. Ge et al*.*[150] have synthesized high-crystalline quality MAPbBr_3_ single crystals, further integrating them with ZnO to construct a perovskite-ZnO heterostructure. Single-crystal perovskite offers several advantages, including lower trap density, higher absorption coefficients, and faster carrier mobility. As shown in Fig. 6d, this heterostructure exhibits potentiation under UV light and depression under green light. This bidirectional light response mechanism stems from the desorption of oxygen ions from the ZnO surface and the formation of a deeper depletion layer. Under UV illumination, photogenerated holes in ZnO facilitate the release of chemically adsorbed oxygen ions, leaving behind photogenerated electrons and inducing PPC. Conversely, under green light, photogenerated holes from the perovskite and electrons from ZnO diffuse further, forming a deeper depletion layer, which leads to a reduction in effective carrier transport and manifests as NPC (Fig. 6e). This heterostructure not only elucidates the synergistic interactions between the perovskite and ZnO but also highlights the potential of such configurations in developing advanced neuromorphic devices that mimic complex synaptic behaviors through light stimuli.

The modulation of conductivity in monolayer perovskite-based AOC synaptic devices is often attributed to the trapping and release of carriers at internal defects under illumination, which affects the width of the Schottky barrier at the interface. Utilizing the lead-free inorganic halide chalcogenide Cs_2_AgBiBr_6_, Sun et al*.* [151] developed an AOC memristor with an Au/Cs_2_AgBiBr_6_/Au structure. This device exhibits bidirectional persistent photoconductivity characteristics under green and red light exposure, facilitating the simulation of various light-controlled excitatory and inhibitory synaptic plasticities. Cs_2_AgBiBr_6_ is noted for its high defect tolerance, particularly with a significant presence of bromine vacancies (${V}_{Br}$) that have relatively low formation energy. Additionally, the interface between the Au electrodes, with a work function of 5.2 eV, and Cs_2_AgBiBr_6_, with a bandgap of 2.4 eV, forms a Schottky barrier [159, 160]. As depicted in Fig. 6f, under 532 nm green light, electrons captured by ${V}_{\mathrm{Br}}$ are excited, leading to the formation of positively charged vacancies ${V}_{\mathrm{Br}}^{+}$ at the interface. This process raises the Fermi level near the conduction band, thus reducing the width of the Schottky barrier at the interface and enhancing device conductivity. Conversely, under 660 nm red light, which is relatively low in energy and insufficient to ionize ${V}_{\mathrm{Br}}$, electrons can be released from shallow defect levels and subsequently recaptured by ${V}_{\mathrm{Br}}^{+}$, decreasing the density of ${V}_{\mathrm{Br}}^{+}$. The resultant broadening of the Schottky barrier leads to persistent NPC. Figure 6g shows the PPF and PPD effects induced by green and red pulses. Working similarly, Cai et al*.* [153] proposed iodine vacancy (${V}_{I}^{o}$) ionization and annihilation modulation mechanisms to explain the bidirectional conductance variations in the Cs_x_FA_y_MA_1-x-y_Pb(I_z_Br_1-z_)_3_-based visual AOC memristor. Unlike typical devices that vary photoconductivity through changes in light wavelength, this device achieves reversible conductivity control using different intensities of red light (630 nm). Specifically, the device conductivity increases under stronger continuous light exposure (11.8 mW cm^−2^) and decreases under weaker illumination (0.9 mW cm^−2^), as depicted in Fig. 6h. The underlying principle relies on the dynamic behavior of iodine vacancies (Fig. 6i). Under strong light conditions, the abundance of photons accelerates the ionization rate of ${V}_{I}^{o}$, outpacing the recombination rate of the positively charged ${V}_{I}^{o+}$. This leads to an accumulation of ${V}_{I}^{o+}$ at the interface, enhancing the device’s conductivity due to increased charge carrier mobility. Conversely, under weaker light conditions, the recombination rate of ${V}_{I}^{o+}$ predominates as the ionization process slows, resulting in a decrease in the concentration of ${V}_{I}^{o+}$ and subsequently, reduced conductivity. Controlling device conductance by modulating defect states provides a subtle way to realize AOC synaptic function.

In particular, photo-induced defective state ionic migration between the perovskite material and the electrode provides an additional mechanism for conductivity modulation. In devices incorporating Ag electrodes, ionic reactions between the surface-treated perovskite materials and metal layers facilitate bidirectional resistive switching under illumination with different light wavelengths. Fu et al*.* [155] have reported an innovative development in AOC devices with their Ag/MA_0.4_FA_0.6_PbI_3_/Ag memristor structure, illustrated in Fig. 6j. Notably, using Pb(SCN)_2_ as an additive during the fabrication process resulted in a significant presence of residual PbI_2_ on the surface of the perovskite film. Intriguingly, devices equipped with Ag electrodes exhibited superior conductivity than Au electrodes. This enhanced performance is attributed to the reaction between PbI_2_ and the deposited Ag electrode, forming highly conductive ionic AgI [161], thereby enriching the device's functionality. As shown in Fig. 6k, this device displays EPSC under warm light stimulation (550–700 nm) and IPSC under near-ultraviolet and cool light (300–500 nm) exposure. Leveraging these optical properties, the device facilitates bidirectional switching of resistance states using 450 and 650 nm light, as depicted in Fig. 6l. This mechanism underscores a significant advancement in harnessing photonic-induced ionic migration between perovskite materials and electrodes, providing an interesting idea for developing versatile and efficient neuromorphic devices.

In the previously mentioned perovskite materials, these materials not only serve as floating gate layers for data storage but also play a critical role as photosensitive layers in the direct processing of optical signals. Their functionality within devices encompasses a variety of distinct mechanisms, which collectively contribute to the dynamic modulation of conductivity and synaptic behavior, enabling real-time processing of light signals. However, despite the significant potential of perovskite materials in AOC synaptic devices, challenges related to their stability and environmental durability remain prominent. The commercialization and practical application of these materials necessitate addressing their stability issues under prolonged light exposure and varying environmental conditions. In addition, the potential environmental and health hazards of perovskite lead content have prompted researchers to explore the possibility of lead-free perovskite materials.

### Organic Materials

Within certain application areas, organic materials have garnered significant interest over inorganic materials due to their low cost, lightweight, flexibility, and ease of processing [162, 163]. Organic materials can be classified based on their physical and chemical properties into several categories, including ferroelectric organic materials, bio-based materials, organic–inorganic heterostructures and all-organic heterojunctions. Each category plays a distinct role in achieving functionalities within AOC devices. For example, ferroelectric organic materials help enhance the polarization effect and promote better charge storage and migration, while bio-based materials offer sustainable alternatives. Organic–inorganic heterostructures combine the beneficial properties of organic and inorganic components, thereby improving the performance of photonic applications. All-organic heterojunctions take advantage of the inherent properties of organic materials to achieve efficient light absorption and charge transport. Synapse devices based on organic materials can be manufactured through a simple solution process, including printing and rotating coatings, making them ideal for mass production. The different functions provided by these different types of organic materials help to improve the performance and applicability of AOC synaptic devices, providing a wider selection of materials for neuromorphic computing. Furthermore, synaptic devices based on organic materials can be manufactured using straightforward solution processes, such as printing and spin-coating, which are conducive to mass production [164, 165]. The diverse functionalities provided by these various types of organic materials enhance the performance and applicability of AOC synaptic devices, offering a broader selection of materials for advancing neuromorphic computing.

#### Ferroelectric Organic Materials

**Fig. 7:**
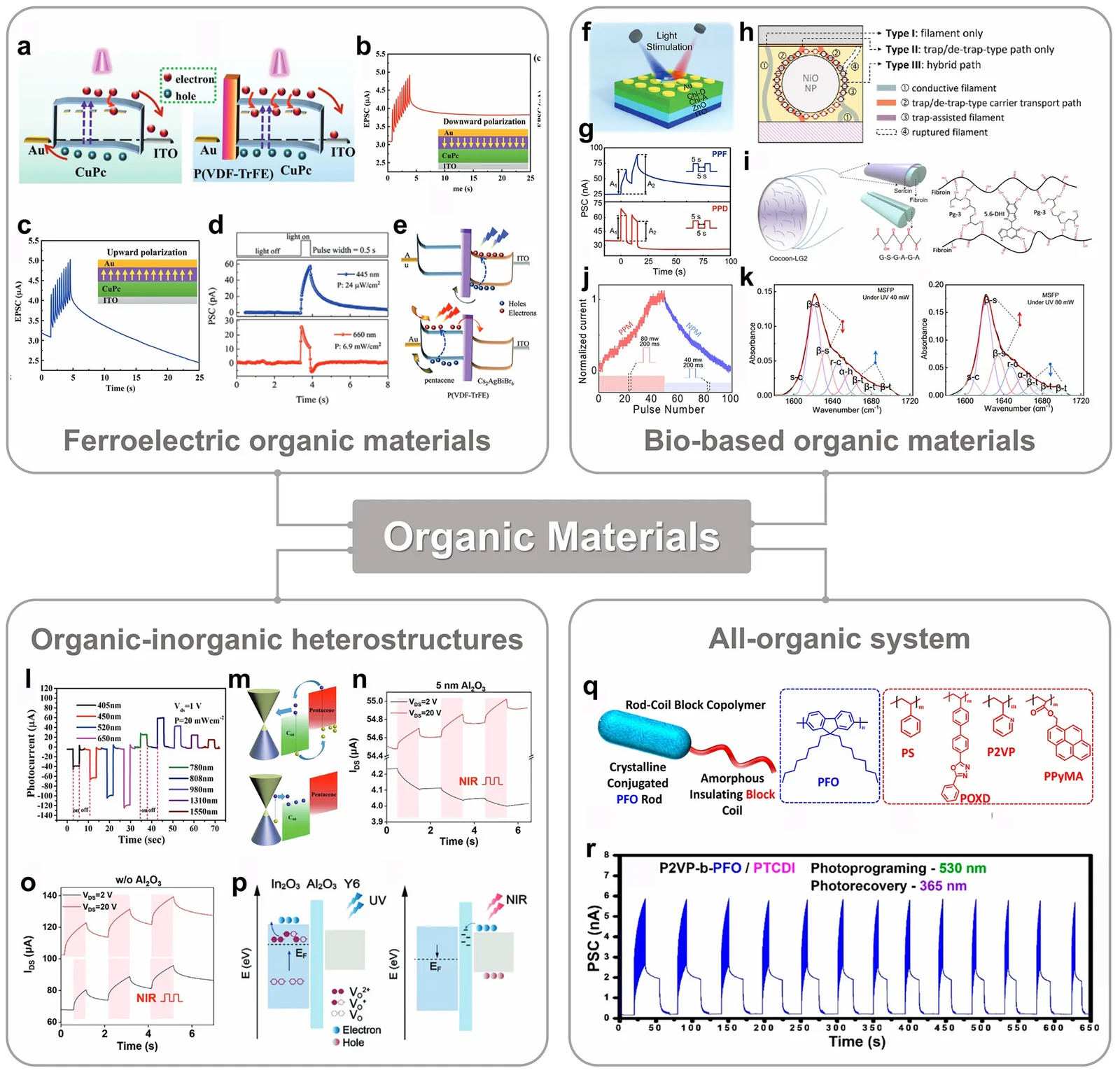
AOC synaptic device based on organic materials. **a** Band diagram of the Au/CuPc/ITO and Au/P(VDF-TrFE)/CuPc/ITO device under light irradiation. The relaxation behaviors of the Au/P(VDF-TrFE)/CuPc/ITO device under **b** downward polarization state and **c** upward polarization state Copyright 2022, Wiley–VCH GmbH [85]. **d** EPSC under 445 nm irradiation and IPSC under 660 nm irradiation. **e** Schematic energy band diagram of the device under the 445 or 660 nm optical stimulation. Copyright 2023, Wiley–VCH GmbH [152]. **f** Device structure of ITO/ZnO/Chl-A/C hl-D/Au. **g** PPF and PPD behaviors of the synapse induced under the light of 430 and 730 nm. Copyright 2024, Wiley–VCH GmbH [35]. **h** Schematic of current transport paths in the AP-NiO hybrid film. Copyright 2022, Elsevier Ltd [176]. **i** Chemical structure of modified silk fibroin protein (MSFP) formed by hydrogen-bond interactions. **j** Optically induced potentiation and depression processes under the continuous 80 mW and 40 mW light pulse. **k** Secondary structure composition diagram of MSFP under 40 mW and 80 mW UV irradiation. Copyright 2023, The Author(s) [177]. **l** PSC at different wavelengths in the graphene/C_60_/pentacene heterojunction. **m** Working mechanism of graphene/C_60_/pentacene heterojunction under visible light and near-infrared illumination. Copyright 2018, WILEY–VCH Verlag GmbH & Co. KGaA, Weinheim [26]. PSC curves **n** without Al_2_O_3_ and **o** with Al_2_O_3_. **p** Working mechanism of In_2_O_3_/Al_2_O_3_/Y6 phototransistor. Copyright 2023, Wiley–VCH GmbH [180]. **q** Chemical structures of the designed PFO BCPs. **r** All-optical potentiation-habituation cycles for the synaptic transistor under sequential 530 and 365 nm light illuminations. Copyright 2023, American Chemical Society [183]

Poly(vinylidene fluoride-trifluoroethylene) (P(VDF-TrFE)) stands as a representative organic ferroelectric material, cherished for its low-cost solution processing capability, remarkable chemical stability, excellent flexibility, and superior biocompatibility[166]. The ferroelectric polarization of P(VDF-TrFE) effectively modulates the decay behavior of photocurrent triggered by light pulses, thereby significantly extending the lifetime of photocurrents. Ji et al*.* [85] integrated copper phthalocyanine (CuPc) with P(VDF-TrFE), demonstrating enhanced device performance attributable to the high binding energy of valence electronic states in P(VDF-TrFE), which establishes an interfacial barrier. In the absence of a P(VDF-TrFE) layer, as illustrated in Fig. 7a, Au forms an ohmic contact with p-type CuPc, while a Schottky contact forms at the p-type CuPc/ITO interface. Contrastingly, in the configuration comprising Au/P(VDF-TrFE)/CuPc/ITO, the interfacial barrier at P(VDF-TrFE)/CuPc impedes the dissociation of nonequilibrium holes toward the Au electrode, thereby significantly extending the lifetime of the EPCS [167, 168]. To elucidate the polarization effects, further investigations delved into the relaxation dynamics of the device under varying polarized states of the P(VDF-TrFE) layer. As depicted in Fig. 7b, c, with downward polarization, an increased barrier induced by positive polarization at the interface prevents the dissociation of nonequilibrium carriers, thereby achieving an extended EPCS lifespan. Conversely, upward polarization diminishes this effect. A similar approach was demonstrated by Lao et al*.* [152] who successfully extended the lifetime of photon-generated carriers within the space charge region by introducing a P(VDF-TrFE) potential well between Schottky energy barriers. They fabricated a self-powered artificial optoelectronic synapse device based on a pentacene/P(VDF-TrFE)/Cs_2_AgBiBr_6_ heterostructure. Due to the distinct absorption characteristics of pentacene and Cs_2_AgBiBr_6_, this device exhibited bidirectional responses to light pulses at 445 and 660 nm (Fig. 7d), successfully simulating synaptic functions such as LTP/LTD and PPF/PPD. The energy potential well formed by the P(VDF-TrFE) layer enhanced the confinement of hole carriers at the P(VDF-TrFE)/Cs_2_AgBiBr_6_ interface. Figure 7e illustrates the energy band alignment of the Au/P(VDF-TrFE)/Cs_2_AgBiBr_6_ device under illumination at 445 and 660 nm. At 445 nm, the Cs_2_AgBiBr_6_ layer generates a substantial number of carriers that accumulate due to the barrier created by the P(VDF-TrFE) layer. The abundance of photogenerated carriers exceeds the depleting carriers, resulting in a continuous increase in the PSC. In contrast, under 660 nm illumination, carriers are primarily generated in the p-type pentacene layer. Due to the barrier, holes quickly migrate to the Au electrode, leading to an increased recombination rate of photogenerated holes and electrons. Consequently, after reaching a peak, the PSC decreases under 660 nm light exposure.

#### Bio-Based Organic Materials

Bio-organic materials, derived from natural polymers, are gaining attention for their sustainability and biocompatibility. Researchers have explored various bio-organic materials to construct AOC devices. Chlorophyll (Chl), a naturally occurring organic semiconductor, has been widely studied for applications in solar cells, photodetectors, and photocatalysis due to its photosensitivity [169–171]. Furthermore, Chl and its derivatives have been extensively used in optoelectronic memristors for photo/electric hybrid operations [172–174]. Chl’s broad light response range and strong absorption capabilities make it an excellent candidate for designing heterostructures in AOC synaptic devices. Recently, Jiang et al*.* [35] proposed a Chl-based heterojunction optoelectronic memristor. The heterojunction, formed from two types of Chl derivatives combined with a ZnO layer (Fig. 7f), exploits the potential barrier at the heterojunction interface and the oxygen vacancy ionization/deionization mechanism in ZnO. This device demonstrated PPF and PPD under light stimulation at 430 nm and 730 nm, respectively (Fig. 7g). In the same domain of plant-based organic materials, apple pectin, a natural polymer, has been applied to non-volatile resistive random-access memory (ReRAM) devices [175]. By integrating light-responsive materials with apple pectin films, the hybrid material benefits from the combined properties. Chang et al*.* [176] embedded NiO nanoparticles (NPs) into apple pectin to develop a non-volatile photonic memory. The main current conduction mechanism in the AP film follows the filament theory, where surface defects in NiO NPs provide trap-assisted tunneling pathways. As illustrated in Fig. 7h, the AP-NiO hybrid film utilizes a type III mixed conduction pathway, enabling writing and erasing behaviors under illumination at different wavelengths. A similar example of bio-based organic materials is the multi-modal-modified silk fibroin (MSFP)-based AOC memristor [177]. Figure 7i illustrates the chemical structure of MSFP. Pure silk fibroin (SFP) does not exhibit photoconductive resistance-switching properties [178, 179]. To address this, researchers incorporated poly(glycerol)-3 (Pg-3) and 5,6-dihydroxyindole (5,6-DHI) into the SFP matrix, both rich in hydroxyl and carbonyl groups. This modification allowed the development of MSFP films with tunable trap states and photoresponsive secondary structures. By applying continuous 405 nm light pulses at intensities of 80 and 40 mW (Fig. 7j), both positive photoconductive memory (PPM) and negative photoconductive memory (NPM) effects were observed. These effects are attributed to the transitions in the secondary structures of the MSFP film under varying light intensities (Fig. 7k), demonstrating the potential of bio-derived materials for tunable AOC synaptic functions in neuromorphic devices.

#### ***Organic***–***Inorganic Heterostructures***

Organic–inorganic heterostructures epitomize the quintessential materials for crafting high-sensitivity AOC devices, thanks to the broad absorption spectrum of organic components and the rapid charge transport capabilities of inorganic materials. Han et al*.* [26] demonstrated bidirectional optical response at different wavelengths by incorporating an organic heterojunction (C_60_/pentacene) onto a graphene layer, marking a pioneering foray into exploiting both materials' synergistic properties. This innovation capitalizes on the hypersensitive photoresponse of the C_60_/pentacene organic small molecule heterojunction in the visible spectrum, paired with the inverse photoresponse of the graphene layer in the near-infrared spectrum. As illustrated in Fig. 7l, the device exhibits a distinct bidirectional photocurrent across different wavelengths. This phenomenon arises from C_60_/pentacene acting as an efficient light-absorbing and photo-carrier separation layer, while graphene serves as an electron conduction pathway. In visible light, the C_60_/pentacene layer effectively absorbs photons and separates photogenerated charge carriers, and electrons are injected into graphene to induce n-type doping (Fig. 7m). In contrast, under near-infrared illumination, charge carriers are mainly generated within the graphene, photogenic holes are trapped in the graphene, and electrons are transferred from the graphene to C_60_, resulting in a positive photocurrent response. Thus, this organic–inorganic heterojunction leverages the complementary properties of its constituents to achieve versatile optoelectronic functionalities, crucial for advancing neuromorphic devices. In subsequent studies on organic–inorganic heterojunctions, Li et al*.* [180] introduced an AOC transistor based on an In_2_O_3_/Al_2_O_3_/Y6 heterostructure. As a comparison, they first evaluated the characteristics of In_2_O_3_/Y6 and In_2_O_3_/Al_2_O_3_/Y6 configurations. Figure 7n–o reveal that by inserting a thin Al_2_O_3_ layer, the device achieved stable bidirectional conductivity modulation and long-term plasticity. This improvement is attributed to the incomplete oxidation of Al_2_O_3_, which transitions the carrier trapping mechanism from hole trapping to electron trapping [181], leading to a negative photoresponse under near-infrared illumination (Fig. 7p). Significantly, this study also validated the feasibility of designing AOC devices using organic–inorganic heterojunctions. The researchers further explored the versatility of this design by replacing Y6 with the organic small molecule semiconductor BTPV-4F, which exhibits a peak absorption at 900 nm [182]. The resulting device demonstrated similar bidirectional photoresponse behavior, reinforcing the potential for tailoring such heterostructures for various optoelectronic applications.

#### All-organic System

Inorganic systems have already made significant strides in the development of AOC devices. While some of the early work involved organic materials, these efforts largely relied on the synergistic interaction between organic and inorganic components. To explore the potential of all-organic AOC devices, Ercan et al*.* [183] designed a series of block copolymers (BCPs) based on polyfluorene (PFO), showcasing the feasibility of AOC transistors built entirely from organic systems. PFO BCPs offer several advantages over inorganic systems, including ideal energy levels, photo-induced charge trapping, and the potential for low-temperature, solution-based fabrication—benefits that favor structural design flexibility and large-scale production [184–186]. As illustrated in Fig. 7q, a range of PFO-based BCPs containing different coil segments, such as polystyrene (PS), poly(2-vinylpyridine) (P2VP), poly(vinylphenyl oxadiazole) (POXD), and poly(1-pyrenylmethyl methacrylate) (PPyMA), were employed to construct phototransistors. The study revealed that the AOC synaptic function of these transistors is significantly influenced by factors such as semiconductor/electret interface morphology and the crystallinity/electron affinity of the BCPs. As a result, PFO-b-P2VP electrets demonstrated enhanced charge trapping and exciton dissociation, enabling wavelength-dependent plasticity transitions. As shown in Fig. 7r, under continuous illumination at 530 and 254 nm, the PFO-b-P2VP electret achieved light-induced programming and recovery operations without the need for gate voltage changes or polarity shifts in the operating voltage. This all-organic system strategy, based on conjugated BCPs, presents an effective device architecture for designing high-performance AOC synaptic devices, offering a promising avenue for future advancements in the field.

## Summary

Each material system possesses distinct functionalities that are essential for realizing the desired synaptic behaviors, underscoring the importance of integrating diverse and multifunctional materials in AOC synaptic devices. This section focuses on four representative material platforms in AOC synapses, including metal oxides, low-dimensional materials, perovskites, and organic materials. A comprehensive understanding of material properties constitutes the most critical step in the fabrication of AOC synaptic devices. The key characteristics of these four material systems are systematically compared in Table 3.

**Table 3:** Comparison of key material systems for AOC synaptic devices

Material system	Common mechanisms	Spectral range	Pros	Cons	Stability	Cost	Scalability
Metal oxides (IGZO, ZnO)	charge transfer, oxygen vacancies ionization/deionization, charge trapping	UV–Visible	High stability, CMOS compatible, mature tech	Slow response (PPC), limited spectral range	High	Low	High scalability
Low-dimensional materials (MoS_2_, Graphene)	charge trapping, gas adsorption/desorption, ferroelectric polarization	UV-NIR	High mobility, atomic thinness, tunable bandgap	Complex transfer process, uniformity issues	Moderate	High	Moderate scalability
Perovskites (CsPbBr_3_)	charge trapping, charge transfer	UV–Visible	High quantum yield, defect tolerance, solution processable	Poor stability (moisture/heat), toxicity (Pb)	Low	Low	High scalability
Organic materials (Polymers)	charge trapping, charge transfer	Visible-NIR	Flexible, biocompatible, molecular tunability	Low mobility, oxidative degradation	Low	Low	High scalability

Metal oxides, owing to their rich oxygen vacancy dynamics, typically exhibit intrinsic persistent photoconductivity, making them ideal candidates for defect- and band-engineering-based AOC synapses. In addition, their excellent environmental stability and compatibility with CMOS processes render them well suited for reliable large-scale integration. By contrast, low-dimensional materials and perovskites exhibit outstanding optoelectronic properties, including broad spectral tunability and high carrier mobility, enabling high-speed and low-energy operation. However, challenges related to long-term environmental stability and large-area uniformity remain to be addressed. Organic materials offer unique advantages in terms of mechanical flexibility, biocompatibility, and molecular design versatility, making them particularly attractive for wearable bioelectronic applications, although their carrier mobility is generally lower than that of inorganic counterparts. From a structural perspective, single-layer device architectures leverage the intrinsic properties of individual materials to establish the foundation for AOC synaptic behavior. In contrast, multilayer heterostructures provide a transformative approach by integrating different materials to exploit complementary functionalities, thereby enhancing design flexibility and device performance.

Therefore, rational device design requires careful matching of intrinsic material properties with the targeted synaptic mechanisms to enable the realization of AOC synaptic devices. By summarizing the characteristics and operating mechanisms of various material systems employed in AOC synapses, this section establishes a solid foundation for developing a reproducible framework for the design of next-generation AOC synaptic devices.

## Material Structure Design Strategy

**Fig. 8:**
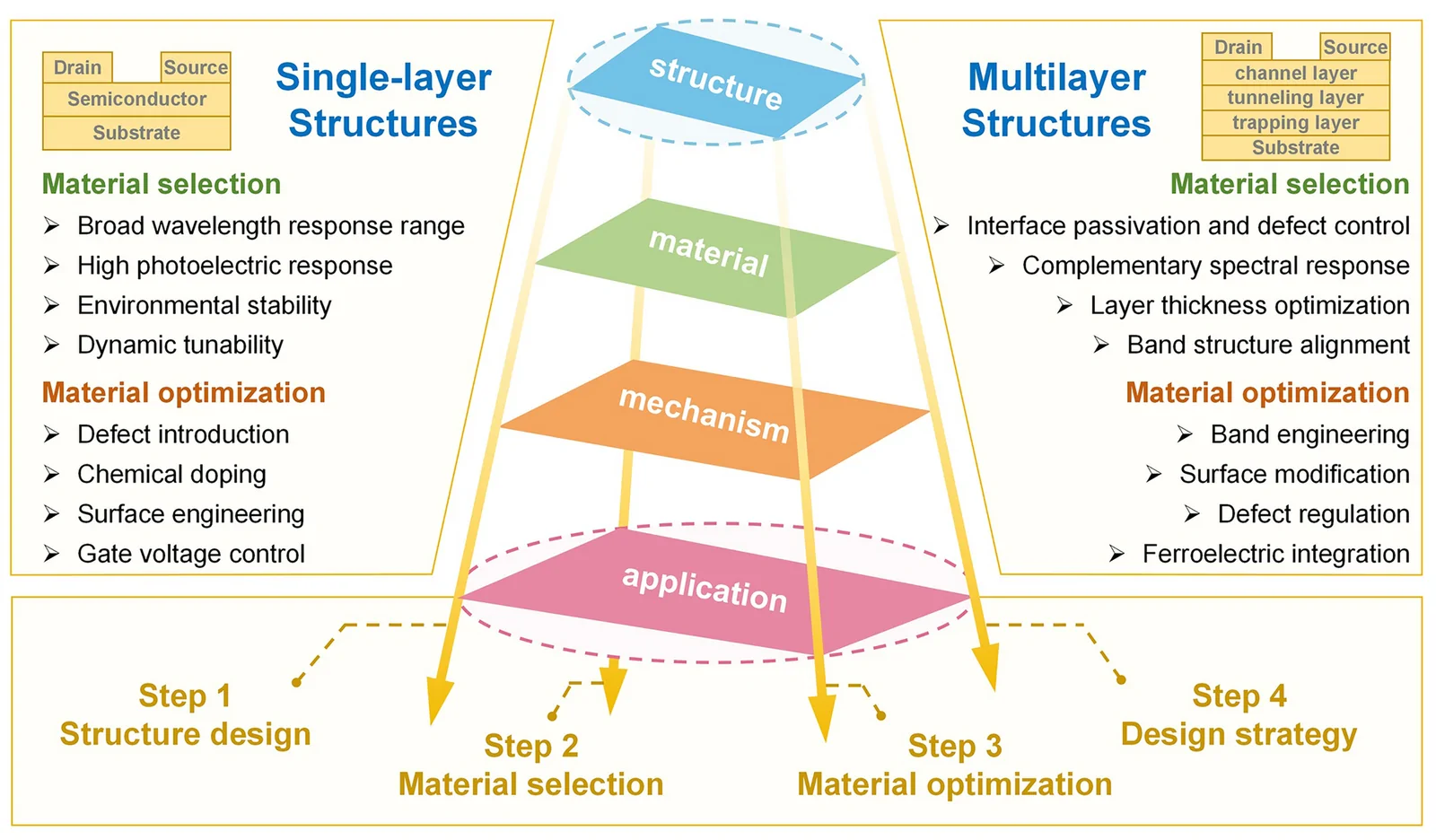
Design strategy diagram of AOC synaptic device from the perspectives of materials and structure

Based on discussions above, we now know that all-optical controlled synapses are very unique and important for neuromorphic computing. This is a novel and creative issue, which is now at the early stage of its development. The sate-of-the-art strategy to design AOC synaptic devices is still the key scientific challenge for neuromorphic computing. Extensive prior research has established that unidirectional optical response is prevalent in photoelectronic synaptic devices [187–191]. However, the unique bidirectional optical response of AOC synaptic devices provides the ability of both optical writing and optical erasing of information, which distinguishes them in the field of neuromorphic simulation, signifying a critical paradigm shift [32, 82, 176]. Table 4 summarizes the AOC synaptic devices in terms of material, structure, performance, mechanism and application, ensuring the universality of the design strategy. The realization of AOC devices relies heavily on the precise engineering of materials and interfaces. The device mechanisms and performance are fundamentally determined by the intrinsic properties of the active/functional materials employed [192]. The optical, electrical, and dynamic properties of these devices are basically derived from the characteristics of their constituent materials [193]. To advance the development of AOC synaptic devices, a comprehensive design strategy that integrates device design, material selection, and material optimization is necessary. This review categorizes device design into single-layer and multilayer structures, refining core principles of material selection and material optimization based on the characteristics of each structure (Fig. 8). Each structural type demands unique strategies and methods. These design strategies provide a foundation for the development of efficient and stable AOC synaptic devices while paving the way for a universal and scalable design framework.

**Table 4:** Summary of synaptic devices

Number	Materials	Device type	Device structure	Operating wavelength (nm)		Repeatability	Mechanism	Application	Year	References
				Potentiation	Depression					
1	Pentacene/C_60_/graphene	Two-terminal	Plane	708–1550	405–650	–	Charge transfer	–	2018	[26]
2	MoS_2_	Three-terminal	–	454	980, 1550	–	Charge transfer	–	2018	[132]
3	P_x_O_y_/BP	Two-terminal	Plane	280	365	–	Gas adsorption/desorption	Logical operation	2019	[68]
4	P_x_O_y_/BP	Two-terminal	Plane	660,280	365	–	Gas adsorption/desorption	Scene simulation	2019	[27]
5	ZnO/PbS QDs/ ZnO	Two-terminal	Vertical	365	980	–	Charge transfer	Image recognition	2019	[28]
6	P_x_O_y_/BP	Two-terminal	Plane	280	365	2000	Gas adsorption/desorption	Image recognition	2020	[30]
7	Bi_2_O_2_Se/graphene	Two-terminal	Plane	635	365	3	Gas adsorption/desorption	Logical operation	2020	[29]
8	Pyr-GDY/graphene/PbS-QDs	Two-terminal	Plane	980	450	10	Charge transfer	Image recognition logical operation scene simulation	2021	[24]
9	O_D_-IGZO/O_R_-IGZO	Two-terminal	Vertical	420	800	10	Oxygen vacancies ionization/deionization	–	2021	[34]
10	CdS/a-IGZO	Three-terminal	–	525	620	–	Charge transfer	Image recognition	2021	[31]
11	BPE-PTCDI/MAPbBr_3_/P2VP	Three-terminal	–	530, 650, 405	254	10	Charge transfer	–	2021	[149]
12	h-BN/WSe_2_/ Al_2_O_3_/BP	Three-terminal	–	637 (50 ms)	637 (100 ms)	50	Charge transfer	Motion detection	2021	[145]
13	ZnO/MAPbBr_3_	Two-terminal	Plane	365	520	10	Oxygen vacancies ionization/deionization	Logical operation	2022	[150]
14	ZnS	Two-terminal	Vertical	White light (0.22 mW cm^−2^ under a prestressed voltage)	White light (0.22 mW cm^−2^)	–	Charge trapping	Visual perception	2022	[69]
15	P(VDF-TrFE)/CuPc	Two-terminal	Vertical	660	445	3	Charge trapping	Scene simulation	2022	[85]
16	PtSe_2−x_	Two-terminal	Plane	980	405	–	Photoconductive and bolometric effect	Logical operation scene simulation	2022	[32]
17	Si NCs/P3HT	Three-terminal	–	532	375	–	Charge transfer	Image recognition	2022	[115]
18	Cs_x_FA_y_MA_1-x-y_Pb(IzBr_1-z_)_3_	Two-terminal	Vertical	630 (11.8 mW cm^−2^)	630 (0.9 mW cm^−2^)	7	Charge trapping	Image recognition	2023	[153]
19	NiO nanoparticles-apple pectin	Two-terminal	Vertical	350	550	–	Charge trapping	–	2023	[176]
20	α-In_2_Se_3_	Three-terminal	–	675, 520, 450 (V_GS_ = − 10 V)	675, 520, 450 (V_GS_ = 10 V)	18	Ferroelectric polarization	Image recognition	2023	[33]
21	CsPbBr_3_-QD/PVAn	Three-terminal	–	450	365	5	Charge transfer	–	2023	[58]
22	PTCDI/P2VP-b-PFO	Two-terminal	Plane	530	365	13	Charge trapping	Image recognition	2023	[183]
23	pentacene/P(VDF-TrFE)/ Cs_2_AgBiBr_6_	Two-terminal	Vertical	445	660	–	Charge transfer	Scene simulation	2023	[152]
24	Y6/Al_2_O_3_/In_2_O_3_	Three-terminal	–	365	808	12	Charge trapping	Image recognition	2023	[180]
25	TiO_2_ QDs/graphene	Two-terminal	Plane	635	365	9	Gas adsorption/desorption	Image recognition	2023	[72]
26	IGZO/ZrO_x_	Three-terminal	–	405	890	–	Oxygen vacancies ionization/deionization	–	2023	[200]
27	ZnO/Zn_2_SnO_4_	Two-terminal	Plane	405	633	15	Oxygen vacancies ionization/deionization	Image recognition	2023	[81]
28	Cs_2_AgBiBr_6_	Two-terminal	Plane	532	660	–	Charge trapping	Image recognition	2023	[151]
29	PbS/graphene/TiO_2_	Three-terminal	–	360	905	100	Charge trapping	–	2023	[137]
30	WO_x_/WSe_2_	Three-terminal	–	648	375	–	Charge transfer	Scene simulation	2023	[133]
31	MoS_2_/Ge	Three-terminal	–	532	1550	4	Charge transfer	Image recognition	2023	[25]
32	Modified silk fibroin protein	Two-terminal	Vertical	405 nm, 80 mW	405 nm, 40 mW	–	Charge trapping	Image recognition	2023	[177]
33	ZnAlSnO/SnO	Two-terminal	Plane	635	532	–	Charge transfer	Image recognition	2024	[37]
34	Ag:AgI/MA_0.4_FA_0.6_PbI_3_/Ag:AgI	Two-terminal	Plane	650	450	–	Ion migration	Visual perception	2024	[155]
35	O_D_-IGZO/O_R_-IGZO	Two-terminal	Vertical	350	650	–	Oxygen vacancies ionization/deionization	Image recognition	2024	[36]

Type	Materials	Device type	Device structure	Operating wavelength (nm)		Repeatability	Mechanism	Application	Year	References
				Potentiation	Depression					
36	Chl-D/Chl-A/ZnO	Two-terminal	Vertical	430	730	6	Charge transfer	Visual perception	2024	[35]
37	PdSe_2_ doped by AuCl_3_ solution	Three-terminal	–	1064	473	–	Gas adsorption/desorption	Logical operation	2024	[75]
38	NiO/TiO_2_	Two-terminal	Vertical	480	320	10	Charge transfer	Image recognition	2024	[56]
39	Ag-TiO_2_ nanocomposite	Two-terminal	Vertical	350	570	5	Charge trapping	Motion detection visual perception	2024	[67]
40	Si-doped β-Ga_2_O_3_/ZnO	Two-terminal	Plane	255	370	50	Charge trapping	Logical operation image recognition	2024	[110]
41	Cu_2_O/WO_3_	Two-terminal	Vertical	405, 532	633	10	Charge transfer	Image recognition	2024	[109]
42	ZnO	Two-terminal	Vertical	350, 420, 530	650, 720, 800	20	Oxygen vacancies ionization/deionization	Logical operation image recognition	2024	[92]
43	*a*-Ga_2_O_3_/*a*-Si:H	Three-terminal	–	455	245	–	Charge trapping	Image recognition scene simulation	2024	[66]
44	IGZO/SnO/SnS	Two-terminal	Plane	266	658	8	Charge transfer	Logical operation image recognition	2024	[108]
45	n-ZnO/p-Si	Two-terminal	Vertical	365	980	–	Charge transfer	Image recognition	2024	[119]
46	MoO_x_/ZnO	Two-terminal	Vertical	350	570	–	Oxygen vacancies ionization/deionization	Logical operation image recognition	2024	[201]
47	ZnO/CdSe ZnS/ IDTBT/PEDOT:PSS	Three-terminal	–	635	365	–	Charge transfer	Image recognition	2024	[55]
48	ZnO/CsPbBr_3_	Two-terminal	Plane	365	525	10	Oxygen vacancies ionization/deionization	Image recognition motion detection visual perception	2024	[82]
49	ZnAlSnO/SnS	Two-terminal	Plane	360	635	18	Charge transfer	Image recognition	2025	[105]
50	SnSe	Two-terminal	Plane	365, 430, 525, 630	255, 295	46	Gas adsorption/desorption	Image recognition scene simulation	2025	[39]
51	In_2_O_3_/BHJ	Three-terminal	–	365	808	–	Charge trapping	Visual perception	2025	[225]
52	(PMA)_2_PbCl_4_/PDVT-10	Three-terminal	–	310	808	–	Ferroelectric polarization	Visual perception	2025	[226]
53	MoS_2_	Three-terminal	–	520, 1310	980	–	Charge transfer and bolometric effect	Image recognition	2025	[224]
54	ZnSiSnO/SnO	Two-terminal	Plane	635	400	70	Charge transfer	Image recognition logical operation	2025	[104]
55	Ga_2_O_3_/WSe_2_	Three-terminal	–	254–275	465–625	–	Charge trapping	Image recognition	2025	[38]
56	Be_2_Se_3_	Two-terminal	Plane	355–405	532–1064	–	Gas adsorption/desorption	Logical operation	2025	[230]
57	In_2_O_3_/perovskite	Two-terminal	Vertical	685	390	–	Gas adsorption/desorption	Image recognition	2025	[231]
58	ZnO-UCNPs	Two-terminal	Vertical	350	980	100	Gas adsorption/desorption	Logical operation motion detection	2025	[218]
59	ZnO/Nb_2_O_5_	Two-terminal	Plane	620	460	200	Charge trapping	Image recognition	2026	[232]

### Single-Layer Structures

Single-layer AOC synaptic devices capitalize on the unique optoelectronic properties of materials to achieve diverse photoresponsive behaviors through mechanisms such as defect trapping, dynamic ionization, and interfacial dynamics. For instance, the surface or defect states of two-dimensional materials provide enhanced charge trapping capabilities [69, 132]. Oxide semiconductors utilize oxygen vacancy ionization and deionization to support both PPC and NPC behaviors [92]. Defect ions in perovskite materials effectively regulate charge trapping and release under illumination [151]. Ferroelectric materials can achieve opposite photocurrent directions under the same optical signal by modulating the applied electric field [33]. To achieve single-layer AOC synaptic devices, the selection of materials with the following characteristics should be considered:

1. *Dynamic tunability:* Materials with dynamic regulation capabilities are essential for achieving reversible conductance modulation under optical stimulation. By controlling oxygen vacancies [36], defect states [69], or applying external electric fields (e.g., ferroelectric polarization [33]), these materials enable dynamic and reversible tuning of conductance. This tunability forms the foundation for emulating synaptic behaviors.
2. *High photoelectric response*: High-efficiency photoelectric conversion is crucial for single-layer materials, allowing them to achieve effective light absorption and carrier generation even at minimal thickness. These properties satisfy the low power, high-speed response requirements of AOC synaptic devices.
3. *Broad wavelength response range*: Better spectral response behavior for specific bands, covering as wide a range of spectral responses as possible [132]. Materials with a broad wavelength response range can efficiently convert a wide spectrum of light, making them promising candidates for AOC synaptic devices.
4. *Environmental stability*: High durability is essential to withstand external environmental factors such as illumination, humidity, and temperature. Robust environmental stability ensures the long-term reliability and performance of these devices in practical applications.

By choosing materials that satisfy the above characteristics, AOC synaptic devices based on single-layer structures can be effectively realized to a certain extent, while simplifying the device design and reducing the preparation cost. Moreover, implementing specific material optimizations can significantly improve these properties. For example, defect sites can be created or reconfigured through techniques such as tuning the growth atmosphere or thermal annealing to optimize their charge trapping and release properties, which further facilitates dynamic and reversible reactions of defect states in monolayer materials, including gas adsorption/desorption and oxygen vacancy ionization/deionization mechanisms, thereby enhancing device performance [39, 92]. The Chemical doping such as tuning chemical composition and arrangement of atoms within materials, as well as modifying electronic properties through additives or doping substitutions, can enhance carrier mobility and stability [32, 75]. The surface engineering such as surface passivation and surface plasma treatment has the ability to improve the interface properties without adding additional layers [68, 133]. Also, employing a three-terminal device and utilizing the gate voltage control technique can dynamically regulate the material's conductivity and carrier density, achieving AOC synaptic device responses under gate-controlled states [33].

Despite their potential, single-layer structures are not yet widely utilized in AOC synaptic devices [37, 105]. First, single-layer structures capable of achieving bidirectional photoresponse are extremely rare and often require intricate material engineering approaches, such as doping or surface modification, to realize the desired functionality. Second, the spectral response range of single-layer materials is inherently constrained. With fixed bandgaps and optical absorption properties, their spectral response is typically limited to specific wavelength regions, reducing their suitability for broad-spectrum applications. Finally, performance optimization in single-layer structures is inherently challenging. Their functionality is heavily dependent on intrinsic properties, leaving limited flexibility to balance competing factors such as carrier separation efficiency, photoelectric conversion rates, and response speeds. These limitations often prevent single-layer structures from meeting the diverse demands of AOC synaptic devices. Consequently, multilayer structures offering greater flexibility in design, broader spectral response, and enhanced performance tuning, have become the more common choice in this field.

### Multilayer Structures

An increasing number of studies have focused on designing multilayer structures. Multilayer material structures encompass heterojunctions of different materials, composite structures, or multifunctional layers. By leveraging functional division, spectral extension, and interface optimization, multilayer materials facilitate more complex photoresponsive behaviors and wider spectral coverage compared to single-layer structures.

Multilayer structures typically consist of semiconductor channel layers, charge trapping layers, and tunneling layers, each playing a distinct role in light capture, carrier transport, and dynamic regulation [84]. The semiconductor channel layer serves as the core component, responsible for carrier transport. These materials require high mobility and stable photoelectric responses to ensure rapid and efficient carrier generation and transport under optical stimulation. The charge trapping layer captures and releases carriers to enable synaptic behaviors such as LTP and LTD. The trapping properties are governed by defect states or floating gate structures, with trapping depths typically ranging from 0.2 to 0.5 eV [194]. These depths can be tuned to adjust storage times and release rates. The tunneling layer acts as a critical module for charge injection and transport in AOC devices. Materials used for this layer typically exhibit wide band gaps and low conductivity, positioning them as essential components in band alignment and barrier formation [195]. The presence of a tunnel layer not only facilitates effective charge injection but also forms a well-matched interface with the semiconductor layer. Through advanced interface engineering and interlayer coupling, multilayer structures enable the realization of complex synaptic behaviors, such as spectral selective responses and bidirectional optical modulation. To meet the design requirements for realizing the synaptic behavior of the AOC, the selected multilayer materials must exhibit the following characteristics:

1. *Band structure alignment*: Proper heterostructure design is critical for effective light-to-carrier conversion and transport. Type-II band alignment, especially Z-type heterojunction, is most commonly employed in heterojunction-based AOC devices. Characterized by staggered bandgaps, Type-II heterojunctions enable spatial separation of electrons and holes [196, 197], minimizing recombination losses and supporting persistent photocurrents essential for synaptic behavior.
2. *Complementary spectral response*: An efficient AOC device requires high absorption coefficients, minimal reflectance losses, and optimized thickness to balance light penetration and carrier transport. Multilayer material systems leverage complementary absorption properties to achieve broadband spectral response from ultraviolet (UV) to near-infrared (NIR). For instance, UV-responsive materials (~ 3.0–4.0 eV) [198] can be paired with infrared-sensitive materials (~ 1.2–1.5 eV) [199] to cover a broader spectrum, ensuring maximal light harvesting and responsiveness across multiple wavelengths.
3. *Layer thickness optimization*: Thickness design plays a pivotal role in ensuring efficient light absorption and carrier transport. The top layer, typically serving as the light absorption layer, should be 10–50 nm-thick to optimize light penetration and carrier generation [26, 81, 82, 150, 200]. The bottom layer, functioning as the charge transport substrate, is usually 50–150 nm-thick to provide structural support and effective carrier collection while minimizing recombination losses [37, 110, 115, 152]. If band misalignment between layers hampers carrier transport efficiency, an interfacial tunneling layer with a thickness of 1–10 nm can be introduced [24, 28, 137, 145, 180]. This layer enhances charge separation and directional transport, facilitating bidirectional optical control critical for synaptic behavior.
4. *Interface passivation and defect control*: Smooth interlayer transitions are essential to minimize recombination losses and maximize responsiveness [108]. Incorporating passivation layers or interfacial modifications reduces defect states at the interface, enhancing carrier transport efficiency and improving the long-term stability of the device.

### Optimization Approaches

In general, multilayer structures are commonly used to realize all-optically controlled synapses. To further enhance the performance of AOC synaptic devices, strategic material selection and optimization are crucial. Here are several targeted approaches that can be implemented:

1. *Band engineering*: Optimizing the band structure of materials through doping or the stacking of heterostructures allows for precise control over the positions of conduction and valence bands, ensuring an accurate photonic response [193]. Incorporating 2D materials known for their high carrier mobility can significantly enhance the speed of photogenerated charge transfer, crucial for fast and efficient device performance.
2. *Surface modification*: Applying surface modification techniques, such as the introduction of functional modifiers like noble metal nanoparticles, can significantly influence the adsorption and desorption behaviors of gases, particularly oxygen.
3. *Defect regulation*: The use of nanomaterials such as nanodots or quantum dots can greatly enhance defect trapping capabilities due to quantum confinement effects [115, 137]. This allows for controlled defect capture and release. Modulating the concentration of oxygen vacancies by adjusting atmospheric conditions during material synthesis or through doping is another effective strategy [201]. Designing multilayer structures that incorporate heterojunctions with varied oxygen vacancy concentrations can further refine carrier movement across interfaces, enhancing the device’s electrical properties.
4. *Ferroelectric integration*: Integrating ferroelectric materials with high polarization strength into the device structure realizes its ability to switch polarization states dynamically [152]. By combining these materials with semiconductors and controlling interface barriers through ferroelectric polarization, precise regulation of charge injection and transport can be achieved, resulting in bidirectional optical responses.

Although the fabrication of multilayer materials is more complex and requires stringent control over interface quality and manufacturing processes, their superior dynamic tunability and high-performance characteristics make them particularly suitable for complex application scenarios. This design strategy offers critical guidance for realizing high-performance AOC synaptic devices while also paving the way for future innovations in material combinations and structural designs. In summary, the design of AOC synaptic devices revolves around device design, material selection, and material optimization. From single-layer to multilayer structures, this systematic design strategy provides a clear and feasible pathway for advancing AOC synaptic device development, enabling robust and scalable solutions for neuromorphic computing and other advanced applications.

## Neural Network Algorithms

**Fig. 9:**
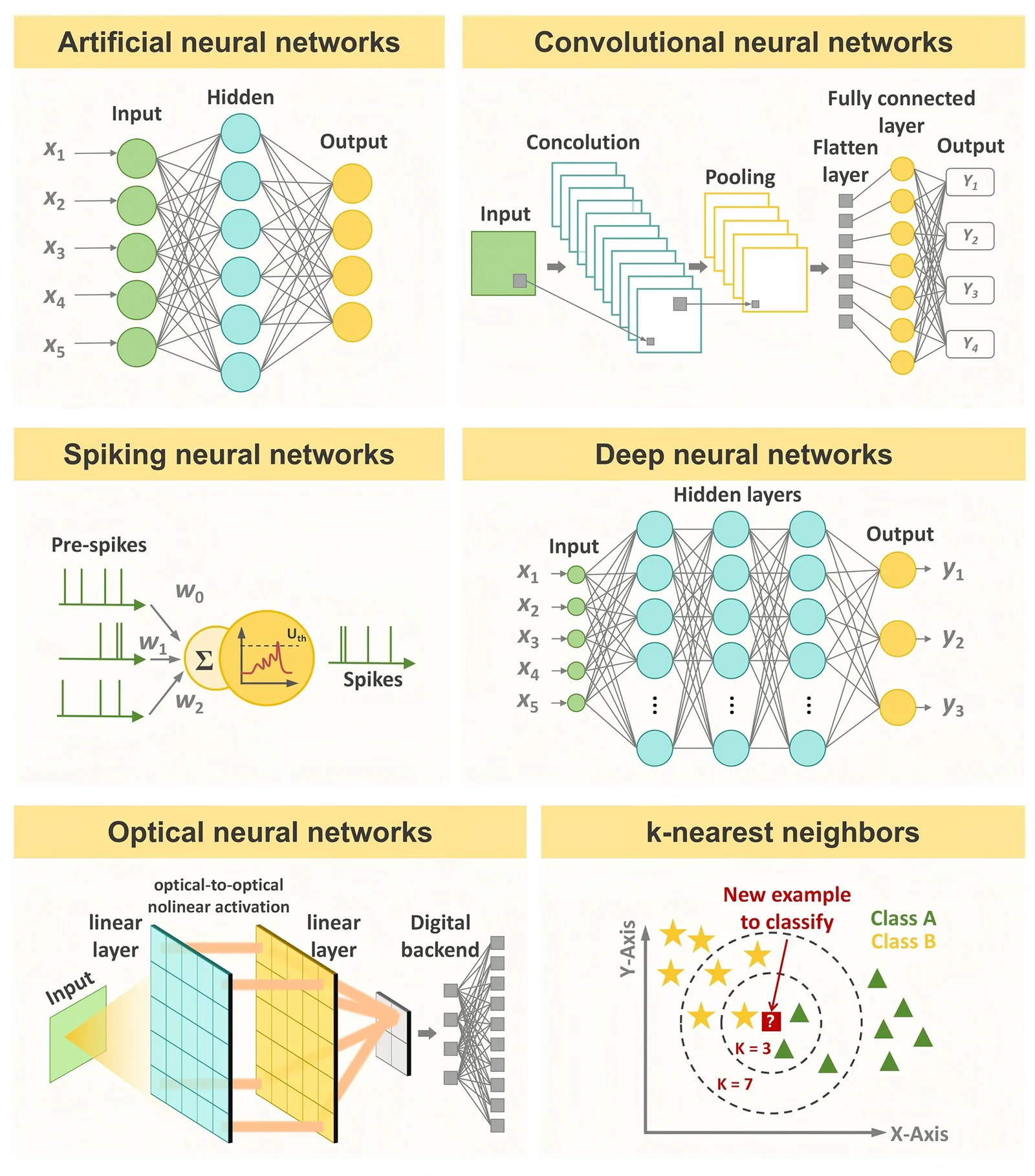
Common architecture diagrams of neural network algorithms

AOC synaptic devices, characterized by bidirectional conductance modulation, low energy consumption, and inherent parallel optical processing, offer a promising hardware foundation for neuromorphic neural network algorithms. The integration of AOC-based sensory synapses with neural network architectures is essential for enabling brain-inspired computation, supporting complex perceptual tasks such as high-precision image recognition, motion detection, and event-driven sensing. This section mainly focuses on the algorithm design and model construction in neural morphological computing tasks. It highlights the representative neural network structures, training methods, and performance evaluation of recognition. It emphasizes the principles and computational processes at the algorithm level, and summarizes the integration path between the algorithm and AOC synaptic devices. Through a comparative analysis, we highlight the major advantages and challenges associated with algorithm-device co-design strategies in neuromorphic computing. Figure 9 illustrates the architectural diagrams of six widely studied neural network models discussed in this section.

### Artificial Neural Networks

Artificial neural networks (ANNs) are computational models inspired by the structural and functional architecture of biological neural systems, in which neurons serve as the fundamental processing units [202]. These neurons are interconnected through weighted links that collectively form a network topology [203]. A typical ANN comprises an input layer, one or more hidden layers, and an output layer, among which the fully connected (FC) layer represents the core component that enables robust pattern recognition and classification capabilities. In an FC configuration, each neuron in the input layer connects to every neuron in the output layer, and synaptic weights are dynamically optimized to adjust connection strengths. This allows output neurons to integrate weighted input matrices efficiently, thereby extracting and identifying salient features from the incoming data [204]. Among neuromorphic algorithms implemented with AOC synaptic devices, ANNs currently exhibit the highest level of technological maturity and demonstrate stable performance in low-complexity classification tasks. Embedding ANN functionality directly within sensing modules to enable real-time, on-chip inference represents a highly integrated approach that can substantially reduce system-level energy consumption while enhancing computational throughput. As one of the earliest and most fundamental forms of ANN algorithms, the multilayer perceptron (MLP) is a basic feedforward neural network well suited for linearly separable datasets. In MLP, each synaptic connection can be directly implemented using an AOC device, whose conductance state encodes the synaptic weight. Optical writing and erasing pulses modulate conductance levels to emulate LTP and LTD, enabling in situ learning without conventional electronic weight updates. Such architectures have demonstrated stable performance in low-complexity tasks such as modified National Institute of Standards and Technology (MNIST) handwritten-digit classification and are well suited for highly integrated, low power edge-sensing systems.

### Convolutional Neural Networks

While FC layers offer essential computational capabilities, they become inefficient when handling high-dimensional visual inputs due to redundant connectivity and limited feature extraction efficiency. Convolutional neural networks (CNNs) overcome these limitations through spatially localized convolutional kernels and weight sharing, enabling efficient extraction of edges, textures, and hierarchical features [205]. A CNN typically comprises convolutional, pooling, and fully connected layers. Convolutional kernels extract local image features, such as edges and textures, in a manner analogous to early visual cortex processing. Subsequently, pooling layers downsample the extracted feature maps, reducing dimensionality, suppressing redundant parameters, and mitigating the risk of overfitting [206]. The processed feature representations are then fed into FC layers to generate the final output. Owing to weight sharing and spatial locality, CNNs exhibit superior performance in image-analysis tasks compared to traditional neural network architectures. Integrating CNNs directly with AOC synaptic devices offers further efficiency gains. Arrays of AOC devices can function as optical convolutional kernels, enabling feature extraction tasks such as edge detection and texture recognition through multi-wavelength optical modulation of device conductance. Meanwhile, the non-volatile conductance states of AOC devices can store intermediate feature maps, effectively serving the role of pooling layers and reducing the need for redundant data transfer. Leveraging the intrinsic parallelism of optical signals markedly enhances computational throughput, enabling sensor-edge implementations of CNN architectures that deliver low power consumption and high processing speed.

### Spiking Neural Networks

Spiking neural networks (SNNs) approximate the temporal coding mechanisms of biological neurons by encoding information in discrete spikes [207]. Learning follows STDP and SRDP, enabling ultralow energy, event-driven computation. These characteristics endow SNNs with exceptionally low energy consumption and the capacity to process temporal information, making them highly suitable for neuromorphic computing platforms and ideal for energy-efficient, event-driven, real-time sensory processing.

AOC synaptic devices enable hardware level implementation of STDP-like behavior by modulating conductance according to the temporal interval between pre- and postsynaptic optical pulses. Short intervals induce LTP-like increases in conductance, whereas longer intervals induce LTD-like reductions. SRDP can likewise be replicated by modulating device conductance through optical pulse frequency, with high-frequency stimulation enhancing conductance and low-frequency stimulation suppressing it. Moreover, the fully optical modulation of AOC devices eliminates Joule heating associated with electrical pulses, effectively overcoming the thermal bottlenecks that restrict the scalability of conventional SNN hardware.

### Deep Neural Networks

Deep neural networks (DNNs) extend ANNs through multilayer abstraction, enabling extraction of complex and high-level representations from high-dimensional sensory inputs. Emerging self-supervised and unsupervised learning techniques further enhance their applicability to perception tasks without the need for handcrafted feature engineering [208]. Integration of DNNs with AOC synaptic devices centers on in-sensor computing and precise weight control. AOC sensor arrays can directly feed optical sensory signals into the input layer by mapping incident optical patterns into device conductance states. This enables in situ preprocessing and drastically reduces data transfer overhead. The multi-level conductance precision of AOC devices supports the fine weight granularity required by deep architectures, mitigating quantization errors typical of electronic hardware. As a result, AOC-based DNNs show strong performance in complex scene recognition and multisensor fusion tasks, and further benefit from transfer learning to enhance generalization while markedly reducing the volume of labeled data needed for training.

### Optical Neural Networks

Optical neural networks (ONNs), which exclusively utilize optical signals, such as intensity, wavelength, and phase, for data transmission and computation, leverage the intrinsic parallelism, ultrahigh speed, and low energy consumption of light to achieve large-scale pattern recognition [209]. ONNs inherently benefit from diffraction-limited parallelism and near-speed-of-light signal propagation. Coupling ONNs with AOC synaptic devices unlocks a fully optical computing pathway where synaptic weights are modulated directly through optical signals. This eliminates energy-expensive electro-optic conversions and reduces latency bottlenecks. For optical pattern recognition tasks, AOC device arrays can serve as optical convolutional kernels, performing feature extraction via parallel optical propagation on nanosecond timescales. Although practical speed is influenced by device response times and optical losses, the architecture remains fundamentally advantageous for high-throughput optical sensing and recognition tasks.

### k-Nearest Neighbors

The k-nearest neighbors (KNN) algorithm provides a lightweight, nonparametric approach to classification by comparing new inputs with stored samples based on distance metrics [210]. Owing to its training-free nature, KNN is highly attractive for real-time, low-complexity edge applications [211]. AOC device arrays offer a natural means of implementing KNN through non-volatile storage of historical sensory patterns. Each sample can be encoded as a distinct conductance distribution within an AOC array. Upon receiving new optical inputs, induced conductance changes can be compared with stored patterns to determine similarity, enabling rapid classification. While AOC-based KNN implementations remain in the exploratory stage, their simplicity, low latency, and energy-efficient storage make them compelling for lightweight neuromorphic edge computing.

Across different levels of task complexity and efficiency requirements, each neural network algorithm exhibits unique advantages when integrated with AOC synaptic devices, forming a set of complementary computational strategies. Table 5 summarizes and discusses the defining features of representative neural network algorithms, their core advantages, and their relevance to AOC-based synaptic hardware. MLPs, as representative ANNs, are well suited for low-complexity classification tasks. CNNs demonstrate strong performance in image processing applications. SNNs excel in ultralow power temporal information processing. DNNs effectively handle complex, high-dimensional sensory data. ONNs enable ultra-fast, parallel optical signal computation, and KNN provides a lightweight, training-free solution for edge intelligent applications. These integration schemes not only leverage the intrinsic strengths of AOC synaptic devices, namely low power consumption and massive parallelism, but also extend the functional boundaries of artificial sensory systems by exploiting the algorithmic characteristics of each neural network class. Together, they offer high-accuracy and high-efficiency pathways for implementing advanced perceptual tasks such as image recognition and motion detection.

**Table 5:** Representative neural network algorithms and their integration with AOC synaptic devices

Neural network type	Algorithmic features	Architecture characteristics	Integration with AOC devices	AOC device characteristics
Artificial neural networks (ANNs)	Dense mapping	Fully connected layers	AOC array stores weight matrix	Multi-level conductance states
				Stable bidirectional weight tuning
	Weight-based learning	High parameter density	Optical pulses induce LTP/LTD-like updates	
				Low optical programming energy
Convolutional neural networks (CNNs)	Local feature extraction	Convolution-pooling blocks	AOC arrays encode convolution kernels	Wavelength-sensitive conductance tuning
			Optical-domain filtering	Fast optical convolution response
	Spatial hierarchy	Receptive fields	Non-volatile feature-map storage	Low crosstalk for parallel operations
Spiking neural networks (SNNs)	Spike-timing and spike-rate coding	LIF neurons	STDP-like tuning via pulse timing	Sub-nanosecond optical pulse response potential
				Time-sensitive conductance modulation
	Event-driven	Time-domain signal propagation	SRDP via frequency-dependent conductance	
				Low heat generation (no Joule heating)
Deep neural networks (DNNs)	Deep nonlinear feature extraction	Multilayer hierarchy	AOC sensors feed optical signals directly into DNN	High dynamic-range conductance levels
				Low drift and noise
	High-dimensional learning	Requires high weight precision	Multi-level conductance supports fine-tuning	
				Large-scale array compatibility
Optical neural networks (ONNs)	Optical-domain matrix multiplication	Diffractive layers or interferometer meshes	Tuning weights directly via optical programming	High-speed optical programming
				Wavelength-multiplex compatibility
	Ultra-fast propagation	Parallel photonic pathways	Eliminates E-O/O-E conversions	
				Low-loss optical signal handling
k-nearest neighbors (KNN)	Training-free	Instance-based architecture	Store exemplars as conductance fingerprints	Highly stable non-volatile states
				Distinct-state mapping for sample storage
				Low-energy readout
	Similarity-based classification	Memory-driven	Optical correlation for similarity evaluation	

Despite these advantages, current efforts to integrate neural networks with AOC devices face four key challenges. First, algorithm-hardware co-optimization remains insufficient. Most existing neural network algorithms were originally designed for electronic hardware, necessitating customized weight-update rules that align with the photoconductive modulation characteristics of AOC synapses. This requires coordinated optimization across algorithmic and device levels. Second, temporal synchronization poses a significant bottleneck. Algorithms such as SNNs and ONNs demand precise control over pulse timing and frequency, but the response speed of current AOC devices remains inadequate for real-time processing of high-speed temporal data. Third, large-scale integration introduces device-uniformity constraints. Many neural network models require millions of synaptic connections, and maintaining uniform performance across large AOC arrays is still a major technological hurdle. Fourth, system-level complexity must be controlled. Auxiliary components, such as optical correlation circuits and wavelength-tuning modules, may increase design overhead, highlighting the need for simplified and scalable integration schemes. Overall, deeper co-design of neural algorithms and AOC device will be essential. Interdisciplinary advances will be required to overcome existing bottlenecks and to enable the transition from basic pattern recognition to more sophisticated autonomous decision-making. Such progress will provide a solid theoretical and technological foundation for next-generation neuromorphic computing systems characterized by ultralow power consumption, high temporal resolution, and robust real-time intelligence.

## Applications

Neuromorphic computing is inspired by the neural structure and computational principles of the human brain, representing an interdisciplinary field that integrates neuroscience, computer science, engineering, and artificial intelligence [212, 213]. This paradigm shift seeks to overcome the limitations of the traditional von Neumann architecture, which separates memory and processing, leading to bottlenecks in speed and efficiency. In contrast, neuromorphic computing enables parallel processing through interconnected nodes, facilitating faster information transmission and computation. Common approaches to designing neuromorphic computing systems involve formalizing the brain’s plasticity and hierarchical learning mechanisms into computational models. Following this, the co-design of hardware and software is undertaken to efficiently realize these models.

With the rapid advancement of AI and the Internet of Things (IoT), the development and integration of AOC synaptic devices signal a pivotal shift toward more efficient, energy-saving computing architectures. AOC synaptic devices form the fundamental building blocks of neuromorphic chips. AOC synaptic devices, a way to integrate sensing and computing functions into a single physical component, can significantly reduce data transmission and simplify the system structure. In AOC synaptic devices, synaptic plasticity is the key to achieving neuromorphic computing. For instance, the reversible modulation of synaptic weights through LTP/LTD is vital for constructing artificial neural networks. Notably, in AOC synaptic devices, the modulation of conductivity is entirely controlled by optical signals, enabling both optical writing and optical erasing of information, which circumvents issues associated with electrical stimulation, such as changes in the device's microstructure and Joule heating. Moreover, within this computing mode of the AOC synaptic device, the AOC synaptic device not only converts external light stimuli into electrical signals for output, but also efficiently processes these stimuli, while also featuring the high computing efficiency of the sensor array structure. This section highlights applications of AOC synaptic devices in areas such as image recognition, logic operations, and optical encryption, emphasizing their potential to enhance processing speed, reduce power consumption, and support large-scale neuromorphic systems more efficiently than conventional methods.

### Image Recognition

The simulation of neural activities is important for the development of ANN. Image recognition is one of the most typical applications and has been widely studied since the advent of artificial synapses. The operating principles of biological vision systems support biomimetic image recognition/processing. In ANN, synaptic devices are commonly used to form cross arrays of synaptic weight layers. This section further explores the specific implementation methods and application verification of AOC synaptic devices in image recognition tasks. It focuses on analyzing the implementation process of mapping algorithms to the device layer, the influence of device parameters on recognition performance, and the performance of the constructed system in actual application scenarios.

**Fig. 10:**
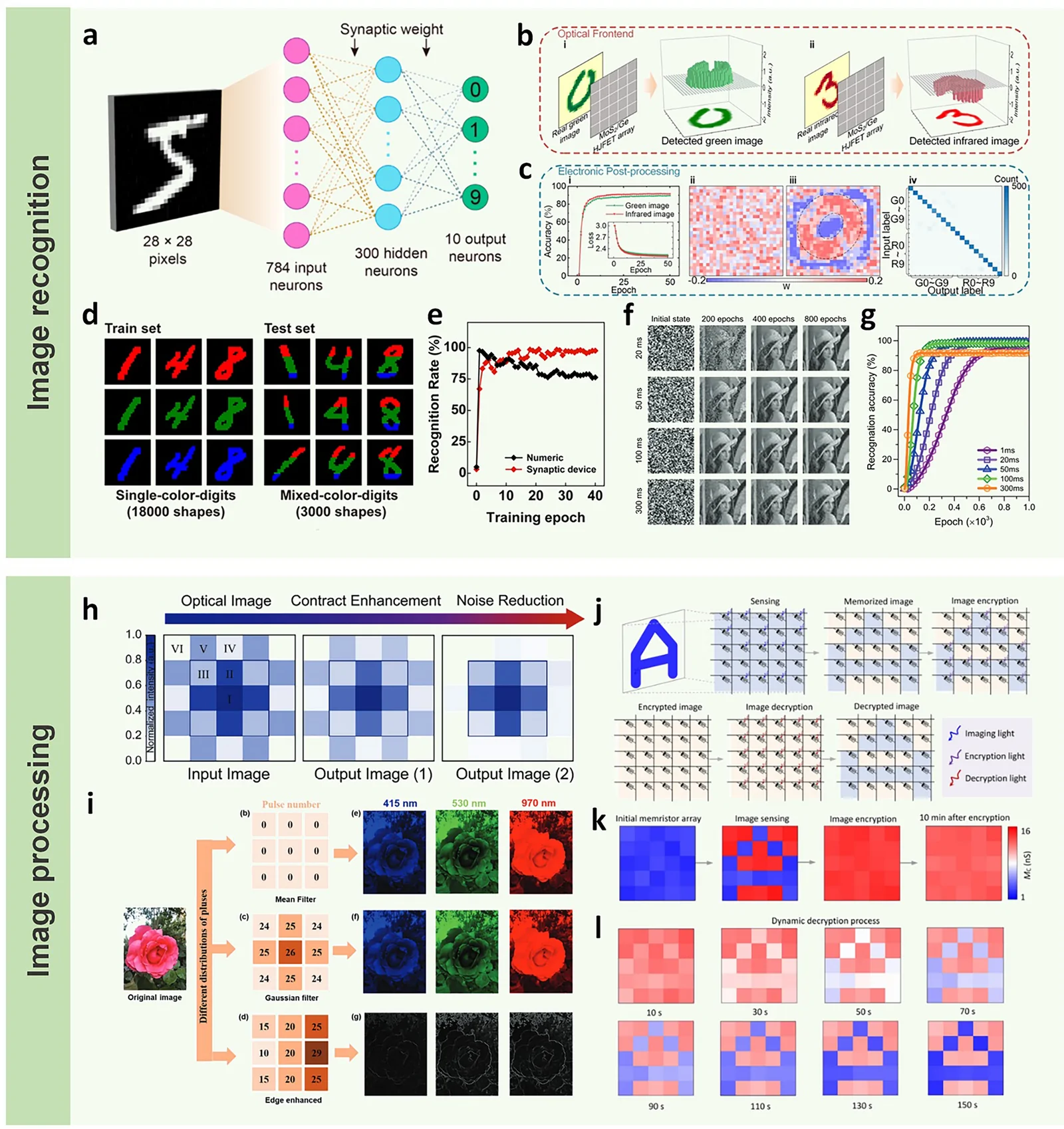
Application of AOC synaptic devices in image recognition/processing. **a** Schematic of a three-layer neural network for recognition of handwritten digits with 28 × 28 pixels Copyright 2021, American Chemical Society [24]. **b** Color-filtering function of the optical frontend. **c** Evaluation results of ANN architecture based on color digit recognition. Copyright 2023, American Chemical Society [25]. **d** Single-color training datasets and mixed-color test datasets are developed for numbers of different shapes. **e** Recognition accuracy as a function of training epochs on the developed test dataset. Copyright 2023, American Chemical Society [33]. **f** Mapping synaptic weights of the input image at initial, 200, 400, and 800 training cycles and for four different experimental datasets. **g** Comparison of image recognition accuracy rate with different LTP/LTD optical pulse width. Copyright 2020, Wiley–VCH GmbH [30]. **h** Image preprocessing of the optoelectronic memristor based on Chl heterojunction, including contrast enhancement (out image (1)) and noise reduction (out image (2)). Copyright 2024, Wiley–VCH GmbH [35]. **i** Image filtering processing, including mean filtering, Gaussian filtering and edge enhancement. Copyright 2023, Wiley‐VCH GmbH [119]. **j** Schematic illustration of the in-pixel sensing, storage, **k** encryption, and **l** decryption. Rights managed by AIP Publishing [36]

The feasibility of image recognition is often evaluated using MNIST. This dataset, with a resolution of 28 × 28 pixels per image, is widely utilized to train ANNs, and the achieved recognition accuracy serves as a benchmark for assessing the learning capability of ANNs. Hou et al*.* [24] implemented this recognition process using a multilayer perceptron (MLP) neural network, as shown in Fig. 10a. The MLP architecture included an input layer with 784 neurons, a hidden layer with 300 neurons, and an output layer comprising 10 neurons to categorize digits 0–9. During training, the model achieved high accuracy, demonstrating robust resilience against varying noise levels. Even under flexible conditions, such as bending or folding, the system maintained a recognition rate of approximately 90%, highlighting its adaptability and potential for real-world applications.

The human visual system discerns a spectrum of wavelengths to recognize colors, a capability rooted in the selective response of retinal photoreceptors to different wavelengths. Similarly, optoelectronic synaptic devices can achieve wavelength selectivity by leveraging materials with varied light absorption rates and external quantum efficiencies under multi-wavelength illumination. Zhang et al*.* [25] reported a wavelength-dependent bidirectional photoresponse using MoS_2_/Ge heterojunction field-effect transistors (HJFET) to recognize and distinguish MNIST digits modified with green and infrared color encoding. This device-based ANN architecture integrates an optical frontend and an electronic processing system to process color-coded digits by converting optical signals into electronic signals for classification. In this setup, colors are encoded as synaptic weights, and light pulses modulate these weights to accurately identify and classify patterns. The optical frontend initially detects the real image and converts it to electronic signals, which are then processed through a preprocessing layer to ensure compatibility with the neural network (Fig. 10b). The output layer, comprising 20 neurons, classifies digit colors based on wavelength filtering. After training over 50 epochs, recognition accuracies of 89.6% and 91.6% were achieved for green and infrared images, respectively, effectively identifying color-coded digits (Fig. 10c). Moreover, devices with broader responsive wavelength ranges can enable the recognition of mixed-color digits. Building on this capability, Chen et al*.* [33] successfully implemented both single- and mixed-color digit recognition based on the multiband response of a-In_2_Se_3_. As shown in Fig. 10d, the researchers generated a dataset of single and mixed-color handwritten digits “1”, “4”, and “8”. In the monochromatic mode, the device modulates its conductance by applying specific wavelength pulses, such as blue, green, or red light. Each wavelength-specific pulse induces distinct postsynaptic currents, thus enabling the device to achieve a corresponding synaptic state. For more complex, mixed-color pattern recognition, the device responds to combined wavelength pulses, generating composite conductance states that represent more intricate synaptic weight configurations. After 40 training cycles, the device achieved an accuracy of 97% for both single and mixed-color digits (Fig. 10e), demonstrating its high efficacy in color differentiation and pattern recognition tasks.

Image recognition can extend to advanced applications such as facial recognition. Ahmed et al*.*[30] demonstrated facial recognition by simulating an ANN to process input photographs. As illustrated in Fig. 10f, the researchers used a 60 × 60 pixel grayscale facial image dataset for their task, with each pixel’s grayscale intensity corresponding to specific light intensity and frequency values fed into the device. In the simulation process, light pulses of different wavelengths, 280 nm and 365 nm, were applied to induce LTP and LTD characteristics in BP photo memory, updating synaptic weights by adjusting device conductance. Through iterative modulation of pulse intensity and frequency, the model effectively differentiated facial features, achieving a high level of recognition accuracy. The results indicated that the final recognition accuracy of a minimally nonlinear system (837 pulses at 1 ms duration) reached 96%, outperforming a system with maximum nonlinearity (204 pulses at 300 ms duration), which achieved only 53.67% accuracy (Fig. 10g). This study highlights the potential of optoelectronic devices in high-accuracy facial recognition.

### Image Processing

By integrating image sensing and preprocessing capabilities, AOC devices use optical signals to modulate synaptic behavior, effectively replicating the retina's ability to perform initial visual computations before transmitting data to higher brain areas. This integration is vital for developing advanced image recognition systems that require efficient, real-time feature extraction, mirroring the parallel processing capabilities of biological vision.

Chlorophyll-based heterojunction memristors can extract essential image features by enhancing contrast and eliminating redundant information [35]. This device responds to different wavelengths of light, such as 430 and 730 nm, by inducing LTP and LTD, respectively. A 5 × 5 memory array was constructed to demonstrate its image preprocessing function, illustrating the full optical modulation for contrast enhancement and noise reduction, as depicted in Fig. 10h. An optical image containing six distinct regions was pixel-mapped into a corresponding set of 430 nm light signals with varying intensities. Comparing the input and output signals reveals that the LTP behavior induced by 430 nm light amplifies the differences between regions of varying brightness, thus enhancing image contrast. Regions IV, V, and VI within the image were defined as noise regions, simulating background noise. Following illumination of Region V with 730 nm light, an LTD effect was triggered, significantly reducing current responses in these noisy regions. This noise suppression highlights the image’s primary features with greater clarity, emphasizing the device’s capability to enhance key image information by reducing irrelevant noise signals.

In addition, synaptic devices with bidirectional photocurrent response and multi-level non-volatile modulation have shown promise for color image preprocessing. Zhang et al*.* [119] explored the image processing capabilities of a 3 × 3 convolutional kernel constructed from a p-Si/n-ZnO heterojunction synaptic device that utilizes tri-color light modulation and detection. By applying different light pulses and multi-level conductance states, the device was able to perform various image processing tasks, as shown in Fig. 10i. Mean filtering smooths the image with consistent light pulses and reduces noise. Gaussian filtering achieves smooth transition through different light intensity distributions in the center and edge. Edge enhancement accentuates the contour information through the conductivity values of the center and surrounding pixels with opposite polarity. These results highlight the considerable potential of AOC synaptic devices for color image processing, making them promising candidates for advanced, low-power visual preprocessing in intelligent vision systems.

Signal encryption is critical for secure signal transmission in the communication field. Traditional image encryption methods often rely on complex cryptographic algorithms and substantial computational resources. In contrast, AOC devices leverage light modulation of their bipolar conductivity characteristics to enable low-power encryption and decryption without the need for additional hardware, making them ideal components for optical encryption applications. Hu et al*.* [36] designed an AOC memristor based on IGZO that facilitates the storage, encryption, decryption, denoising, and destruction of visual images. As illustrated in Fig. 10j, the image encryption and decryption process is executed using a 5 × 5 AOC memristor array. Initially, blue light is used to selectively store an image (for example, the letter “A”) in the array. Subsequently, ultraviolet light is applied to the remaining pixels, altering their conductivity states to match those of the blue light-exposed areas, thereby achieving encryption (Fig. 10k). For decryption, red light is then directed at all pixels, capitalizing on the differences in recovery rates of conductivity under varying light conditions (Fig. 10l). Specifically, UV-irradiated pixels exhibit a larger RESET index under subsequent red light irradiation, resulting in lower amnesia, which in turn facilitates image recovery from the encrypted state. This approach demonstrates the substantial potential of AOC memristors for low-energy visual information security, providing a promising avenue for advancing secure image transmission technologies. In summary, AOC synaptic devices offer significant advantages in image processing, enabling enhanced feature extraction, edge detection, and secure communication, all while reducing computational demands and power consumption.

### Logical Operation

**Fig. 11:**
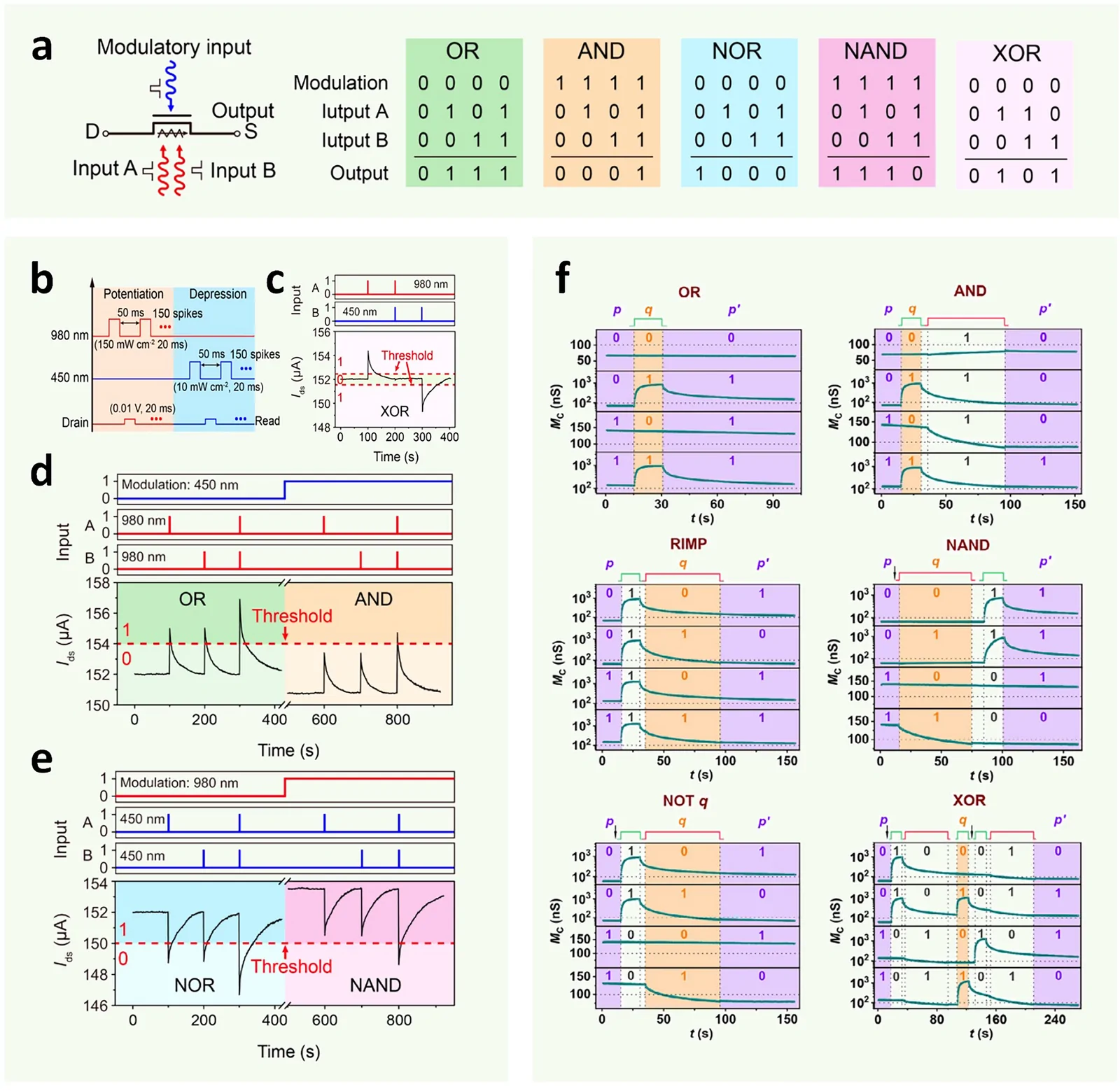
Application of AOC synaptic devices in logical operation. **a** Schematic operation diagram for the logicl functions in the optical synapse. **b** Schematic of the optical training for LTP/LTD curves. Simulation diagram of **c** “XOR”, **d** “OR” and “AND”, and **e** “NOR” and “NAND” logic functions Copyright 2021, American Chemical Society [24]. **f** Non-volatile logic computing in the ZnO-based AOC memristor. Copyright 2022, The Authors. Publishing Services by Elsevier B.V. on behalf of KeAi Communications Co. Ltd [92]

Integrating logic functions within synapses is essential for significantly enhancing the information processing capability of neuromorphic computing systems. In particular, AOC synaptic devices achieve functional switching through reconfigurable operations of PPC and NPC, laying a foundation for programmable optoelectronic logic circuits. Boolean logic, a system of algebraic operations where variables hold truth values (true or false, often represented as “1” and “0”), forms the basis of such computations [214]. In a two-input system, there are 16 Boolean functions, including “OR”, “AND”, “NOR”, “NAND”, and “XOR” (Fig. 11a). In previous work, Hou et al*.* [24] demonstrated simplified optical logic operations using dual optical inputs (A and B). This device achieves bidirectional optical response at 530 and 650 nm (Fig. 11b), allowing for flexible logic modulation via specific wavelengths to perform complex logic tasks collaboratively. For instance, the “XOR” function is implemented by applying different wavelengths (980 nm for input A and 450 nm for input B), yielding a high output only when one of the two inputs is active, rather than both (Fig. 11c). Additionally, “OR” and “AND” functions are achieved by applying 980 nm as both inputs A and B while using 450 nm as a modulation input (Fig. 11d). Conversely, when 980 nm serves as the modulation input with 450 nm as the primary input, the device switches to “NOR” and “NAND” operations (Fig. 11e). This wavelength-dependent control illustrates the versatility of AOC synaptic devices, making them suitable for dynamically reconfigurable logic circuits in future neuromorphic computing architectures.

Non-volatile Boolean logic operations have also been demonstrated in ZnO-based AOC memristors, where computational results are in situ stored as memconductance states [92]. In this device, the initial memconductance serves as one input (p), while a second input (q) is modulated by specific wavelengths of light: 530 nm for positive signals and 650 nm for negative signals. As shown in Fig. 11f the combination of input and control light determines the output logic state, enabling the emulation of various Boolean logic functions. Functions like “AND” and “OR” are achieved through specific sequences of light application, while more complex operations require measuring the initial memconductance or intermediate states. This memristor’s non-volatile nature ensures it retains a stable logic state even after light exposure ceases, making it well suited for logic applications where persistent memory is critical.

### Motion Detection

Detecting and recognizing moving objects has become increasingly vital, including real-time video analysis, military defense, and biological detection [215, 216]. Current motion detection and recognition technologies based on CMOS image sensors are constrained by complex designs that involve redundant modules for sensing, transmission, conversion, processing, and storage [217]. This multi-step architecture often results in bulky and inefficient systems. Additionally, conventional visual sensors lack memory capabilities and are primarily suited for detecting static objects, limiting their effectiveness in dynamic environments. In contrast, AOC synaptic devices present a more adaptive solution by adjusting their conductance in response to incoming light signals, allowing them to process and store information simultaneously. This unique ability to integrate both real-time processing and memory functions within a single device reduces the need for external processing and storage components, resulting in a streamlined, low-power system. Such an adaptive, energy-efficient structure markedly improves motion detection efficiency, making AOC synaptic devices well suited for handling complex visual tasks in dynamic environments.

**Fig. 12:**
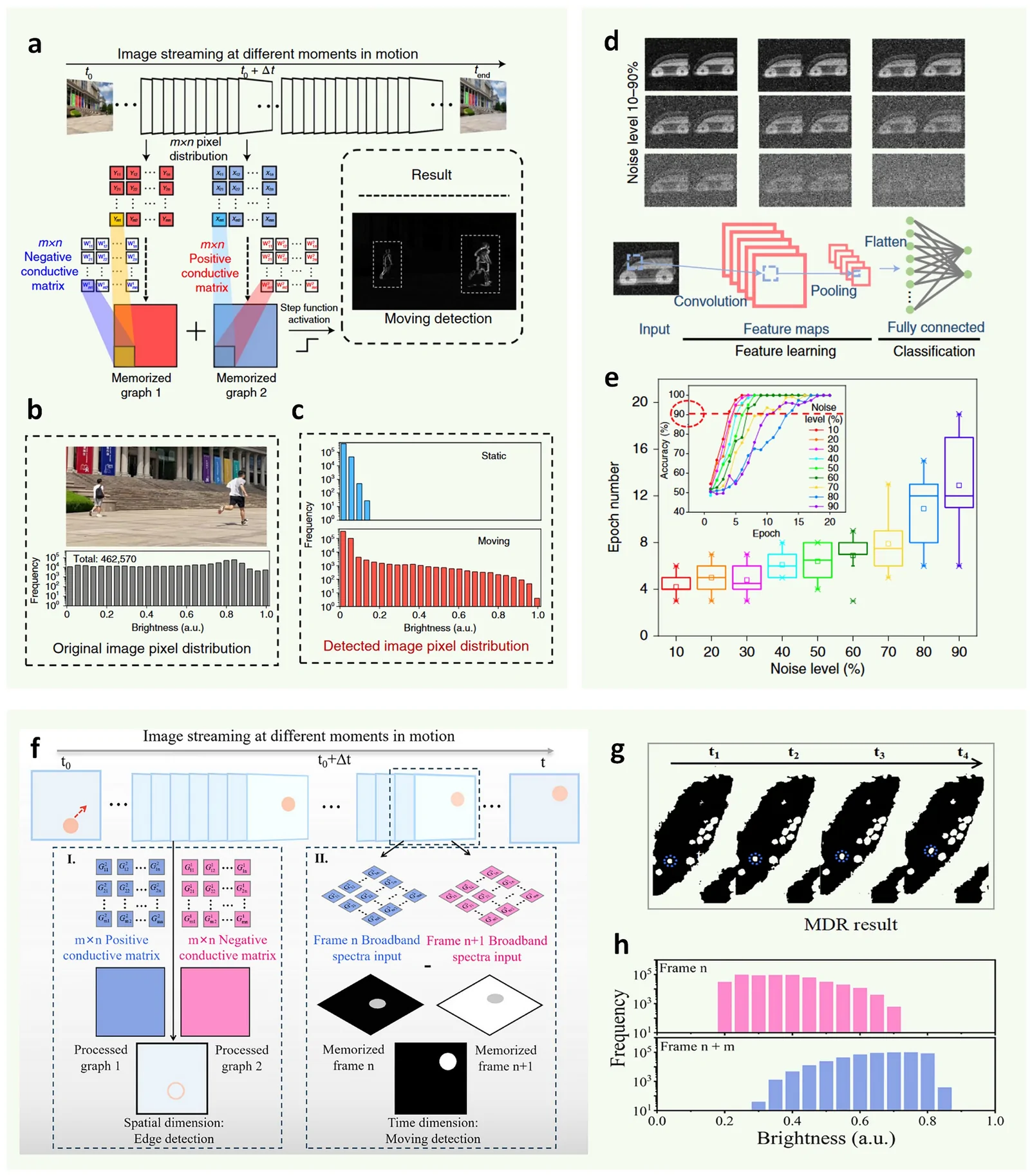
Application of AOC synaptic devices in motion detection. **a** Pixel processing diagram based on positive and negative conductance matrix. **b** Original image and normalized pixel brightness distribution. **c** Pixel brightness distribution after motion detection with and without moving objects. **d** A schematic of the trolley database and CNN recognition process. **e** Car identification accuracy statistics under different noise levels Copyright 2021, The Author(s), under exclusive licence to Springer Nature Limited [145]. **f** Schematics of edge and motion detection based on PPC and NPC. **g** Output results during motion detection. **h** Pixel brightness distribution of images before and after motion detection. Copyright 2025, Wiley‐VCH GmbH [218]

Inspired by the human retina, Zhang et al*.* [145] developed a retina-inspired device that integrates sensing, memory, and computing capabilities within a single structure. This device, based on a BP/Al_2_O_3_/WSe_2_/h-BN heterostructure, exhibits non-volatile positive and negative photoconductivity for red, green, and blue light, thereby simulating the on/off bipolar cell functions in the retina. As illustrated in Fig. 12a, the device employs a positive conductance matrix (W^1^) and a negative conductance matrix (W^2^) to mimic the center-surround antagonistic mechanism of retinal bipolar cells. During motion detection, the images from different time frames are mapped onto W^1^ and W^2^ matrices, and frame-to-frame differential calculations are applied to filter out static backgrounds while emphasizing moving targets. Figure 12b shows the pixel distribution of an initial moving image. In Fig. 12c, if no moving object is present within a given time interval (Δ*t*), the absolute values of the positive and negative conductance matrices are nearly identical, resulting in a near-zero pixel output across the image. However, when a moving object is detected within the time frame, the pixel output shows distinct brightness variations. This brightness contrast enables motion object recognition by analyzing the output pixel values. To further refine the motion detection strategy, the study used a moving red, green, and blue cart as an example and introduced the concept of “separation rate” to minimize ghosting by adjusting the frame differential time. By incrementally increasing the frame differential time (e.g., 33.3, 66.7, and 133.3 ms), ghosting artifacts were significantly reduced in motion images, as shown in Fig. 12d. This result confirms that extending the frame differential time can enhance object separation and improve detection accuracy. To assess the device’s noise robustness, a database of images with varied noise levels (10%-90%) was constructed, simulating real-world environmental interferences like dust and sand. A convolutional neural network (CNN) was then employed to train on these noisy images, testing the device's resistance to interference. As shown in Fig. 12e, the device maintained high recognition accuracy even at elevated noise levels. This robust performance is attributed to its linear response across multiple conductance states, enabling efficient mapping to neural network weights and enhancing recognition accuracy under challenging conditions.

Motion detection is achieved by analyzing the changes between consecutive frames. Recent studies have demonstrated the use of all-optical synaptic devices for monitoring and processing image data, thereby detecting and tracking the movement of dynamic objects [67]. The ZnO-UCNPs memristor reported by Han et al*.* [218] exhibits excellent all-optical modulation, thereby providing a highly energy-efficient method for motion detection. Each memristor unit responds to changes in light within its field of view, recording variations in light intensity and position across each frame. Through differential calculations of consecutive frames and all-optical modulation, the system can effectively identify moving targets within complex backgrounds. As illustrated in Fig. 12f, the changes in continuous frame images are converted into positive and negative photocurrent (PPC and NPC) matrices, allowing for the comparison of these matrix outputs to distinguish between dynamic and static states. Specifically, each frame captured at time t_1_ is transformed into a PPC matrix, which is then compared to the subsequent NPC matrix at time *t*_1_ + Δ*t*. The detected spatiotemporal information is input into the network in the form of optical information *x*(*t*), and the entire dynamic information can be regarded as images at different moments. This comparison reveals pixel changes between the two-time points, effectively capturing the variations in photocurrent across the frames. Furthermore, after conducting local difference calculations, the edges of objects can be effectively extracted, thereby achieving the recognition and tracking of moving targets. Based on the above technical strategies and in combination with the excellent response characteristics of ZnO-UCNPs synaptic devices under near-infrared light, Han et al*.* [218] successfully achieved dual monitoring of the static and dynamic states of chloroplasts, which can not only extract edge features but also capture dynamic information. In edge detection scenarios, still images within a specific frame difference time will correspond to the superposition effect of positive and negative conductance matrices, and ultimately output high-definition edge features (as shown in Fig. 12g). In the motion detection scenario, the positive and negative conductance matrices generated by the moving images at adjacent moments will achieve signal optimization through addition operations. The conductance signals in the static area cancel each other out, while the dynamic information is precisely extracted through the superposition effect of pixel output, as shown in Fig. 12h. It clearly presents the luminance distribution of the chloroplast movement flow at time t_0_ and time t, intuitively reflecting the movement trajectory and dynamic characteristics of the target. This analytical approach enhances the system’s ability to accurately recognize and track moving targets amidst complex backgrounds, significantly improving motion detection accuracy and efficiency.

The detection method based on AOC synaptic devices allows the system to track moving objects in real-time using only optical signal processing, without the need for external sensor input. This capability can efficiently adapt to varying environmental conditions and increase processing speed and responsiveness, which is crucial for applications such as real-time monitoring and dynamic scene analysis. This technology offers a high-performance solution for next-generation smart vision systems, paving the way for more responsive and efficient IoT applications.

### Visual Perception

The diverse types of neurons in the retina enable humans to perceive light, with photoreceptor cells responsible for converting light signals into electrical signals [219]. The functionality of the visual nervous system relies heavily on synaptic plasticity, which underpins the adaptability and efficiency of sensory perception [220]. AOC synaptic devices exhibit significant promise in the realm of visual perception applications.

**Fig. 13:**
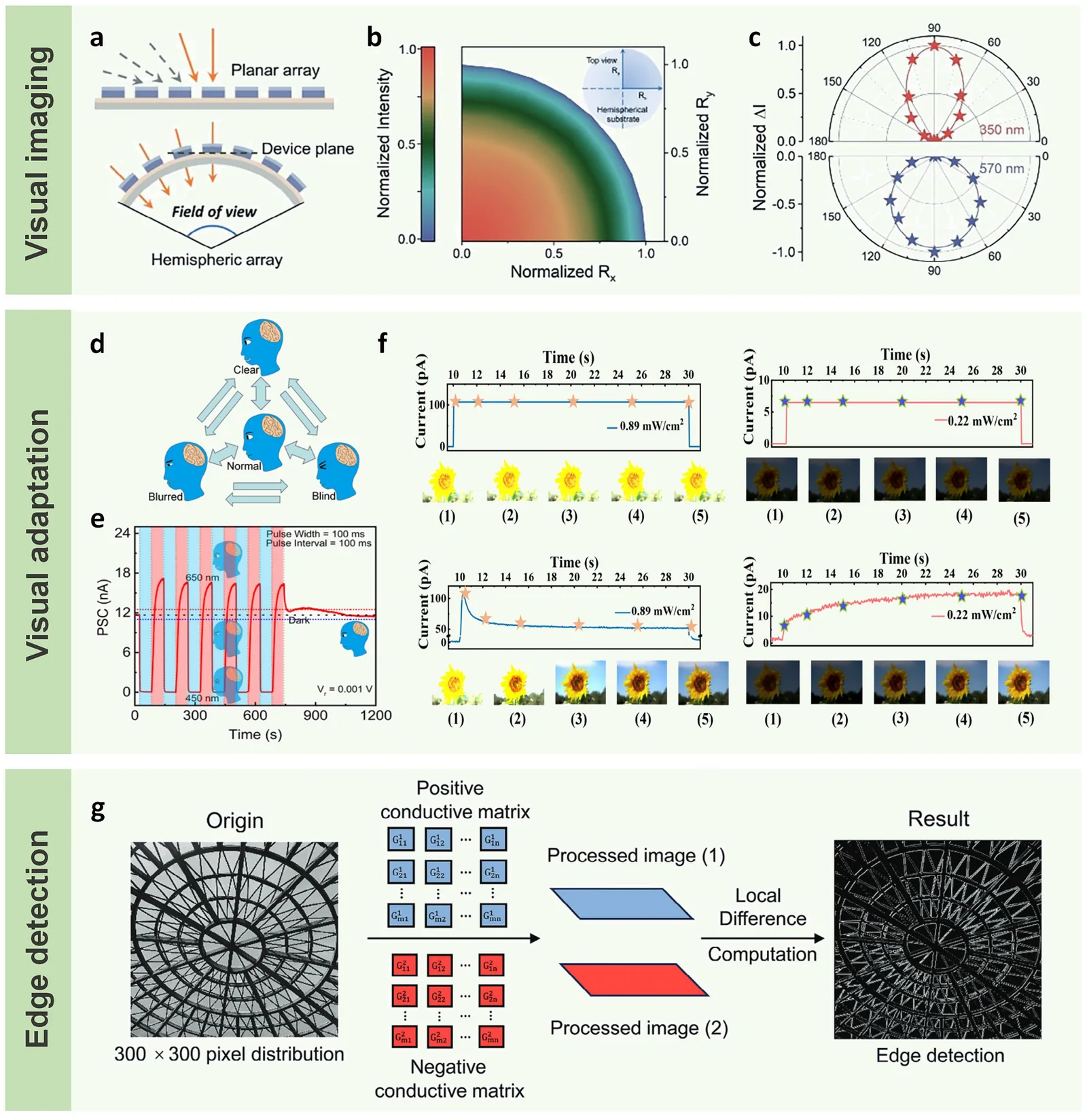
Application of AOC synaptic devices in visual perception. **a** Optical signals from various spatial angles in the planar and hemispheric array. **b** Illumination intensity dependent on the incident angle. **c** Angle-sensitive photocurrents under the irradiation of ultraviolet and visible spike Copyright 2024 The Author(s). Advanced Science published by Wiley–VCH GmbH [67]. **d** Schematic illustration of the all-optical artificial synapse mimicking the adaptive process in humans after 450 nm and 650 nm light stimulations. **e** PSC of the artificial synapse under the 450 nm and 650 nm light. Copyright 2024, American Chemical Society [155]. **f** Comparisons between the images with and without adjustment function in dim and bright conditions, respectively. Copyright 2022, The Authors. Advanced Intelligent Systems published by Wiley–VCH GmbH [69]. **g** Retina-like edge detection implemented in Chl heterojunction-based optoelectronic memristor. Copyright 2024 Wiley–VCH GmbH [35]

To simulate the human retina, Shan et al*.* [67] developed a hemispherical array of photoelectric memristors composed of Ag-TiO_2_ hybrid nanoclusters and sodium alginate (SA). Under stimulation from ultraviolet and visible light, the device demonstrated optically tunable synaptic plasticity. The array was flexibly transferred onto a hemispherical model using a PDMS substrate. As shown in Fig. 13a, this unique hemispherical geometry helps mitigate light attenuation caused by large incident angles, thereby enhancing imaging quality at the edges and achieving a wide field of view. In terms of spatial angle detection, the arrangement of the hemispherical devices increases the spatial angle differences between individual units, resulting in varying illumination intensities. Figure 13b illustrates the differing light intensities received by various memristor units within the hemispherical array. Based on this illumination intensity-dependent plasticity, spatial angle detection can be achieved. Figure 13c presents the excitatory and inhibitory postsynaptic current variations of individual Ag-TiO_2_ nanocluster/SA-based devices at different spatial angles (ranging from 0° to 180°), offering a method for precise spatial angle detection.

Furthermore, AOC synaptic devices can be applied to mimic the human eye’s adaptive response to rapidly changing light conditions, providing significant technical support for autonomous driving and artificial vision systems. Fu et al*.* [155] have demonstrated this by leveraging a reversible switching effect between photo-induced Ag and iodine ions to emulate visual synaptic functions. By alternating between three conditions, 650 nm, 450 nm, and darkness, four distinct visual states emerge, reflecting the reversible adaptation observed in human vision (Fig. 13d). After extended blue light exposure, the device can recover swiftly to a “clear state” upon red light illumination, simulating the mitigation of glare, akin to the human eye’s adjustment when transitioning from a bright setting to a darker environment (Fig. 13e). This rapid adaptation capability is particularly relevant to drivers encountering sudden glare when entering tunnels from bright surroundings. A similar example is the Au/ZnS/Pt device, which independently achieves STP and STD under varying light intensities, enabling it to autonomously regulate its response to external stimuli [69]. For instance, when a vehicle traverses a sequence of alternating bright and dark tunnels, this ZnS-based device can dynamically adjust its output, adapting to the fast shifts from brightness to darkness and vice versa. This self-regulation feature allows the device to adapt rapidly to abrupt lighting changes, preventing issues like overexposure or underexposure in captured images. As depicted in Fig. 13f, white light intensities of 0.22 and 0.89 mW cm^−2^ are used to simulate the contrasting lighting conditions inside and outside a tunnel. A comparison of the adjusted and unadjusted images (top and bottom halves of Fig. 13f, respectively) reveals that the adjusted images maintain clearer visual quality across varied lighting environments. Such exceptional adaptive capacity makes these devices particularly suited for applications requiring rapid response to environmental changes, like autonomous vehicles operating under fluctuating light and weather conditions.

The human visual system relies on retinal edge detection to differentiate object contours and details, an essential step in object recognition and scene understanding [221]. Conventional edge detection methods often depend on complex algorithms and high-energy computational resources, making it challenging to mimic the adaptive nature of biological vision at the hardware level. Retinal bipolar cells achieve this by utilizing the “On-center” and “Off-center” receptive fields (RFs) to detect brightness contrasts, a mechanism that is highly sensitive to changes in luminance and thus integral to effective edge detection [222]. Jiang et al*.* [35] successfully emulated retinal edge detection using a heterojunction AOC memristor composed of ZnO and two chlorophyll derivatives (Chl-A and Chl-D). The edge detection process is illustrated in Fig. 13g. First, an image is converted into a grayscale pixel matrix and mapped through a linear light response to generate separate positive and negative conductance matrices. Edge extraction is achieved through local differentiation along diagonal directions, where the contrast between adjacent pixels serves as a gradient magnitude, highlighting edge features. For refined edge visualization, the positive and negative conductance matrices are offset by one pixel at a 45° angle and then combined. Grayscale levels are adjusted from 0 to 255, producing a final edge detection image where pixels near the object’s edge display a strong gradient while the surrounding regions remain close to zero. This approach effectively enhances edge clarity by emphasizing prominent gradient transitions along object boundaries, providing a low-power, hardware-efficient solution for edge extraction in AOC synaptic devices.

### Scene Simulation

AOC synaptic devices achieve PPC and NPC by modulating the wavelength, frequency, and intensity of light, demonstrating remarkable versatility in replicating complex neural behaviors. Leveraging the distinct optoelectronic properties of various materials, these devices can simulate a wide range of neuronal activities within neural computation models, from rapid action potentials to long-term synaptic plasticity. This adaptability allows AOC synaptic devices to be optimized for specific applications, such as pain sensation emulation, environmental sensing, and complex behavioral prediction. By employing this approach, researchers can design targeted application scenarios that replicate the dynamic characteristics of human and animal neural systems, paving the way for advanced neuromorphic technologies.

**Fig. 14:**
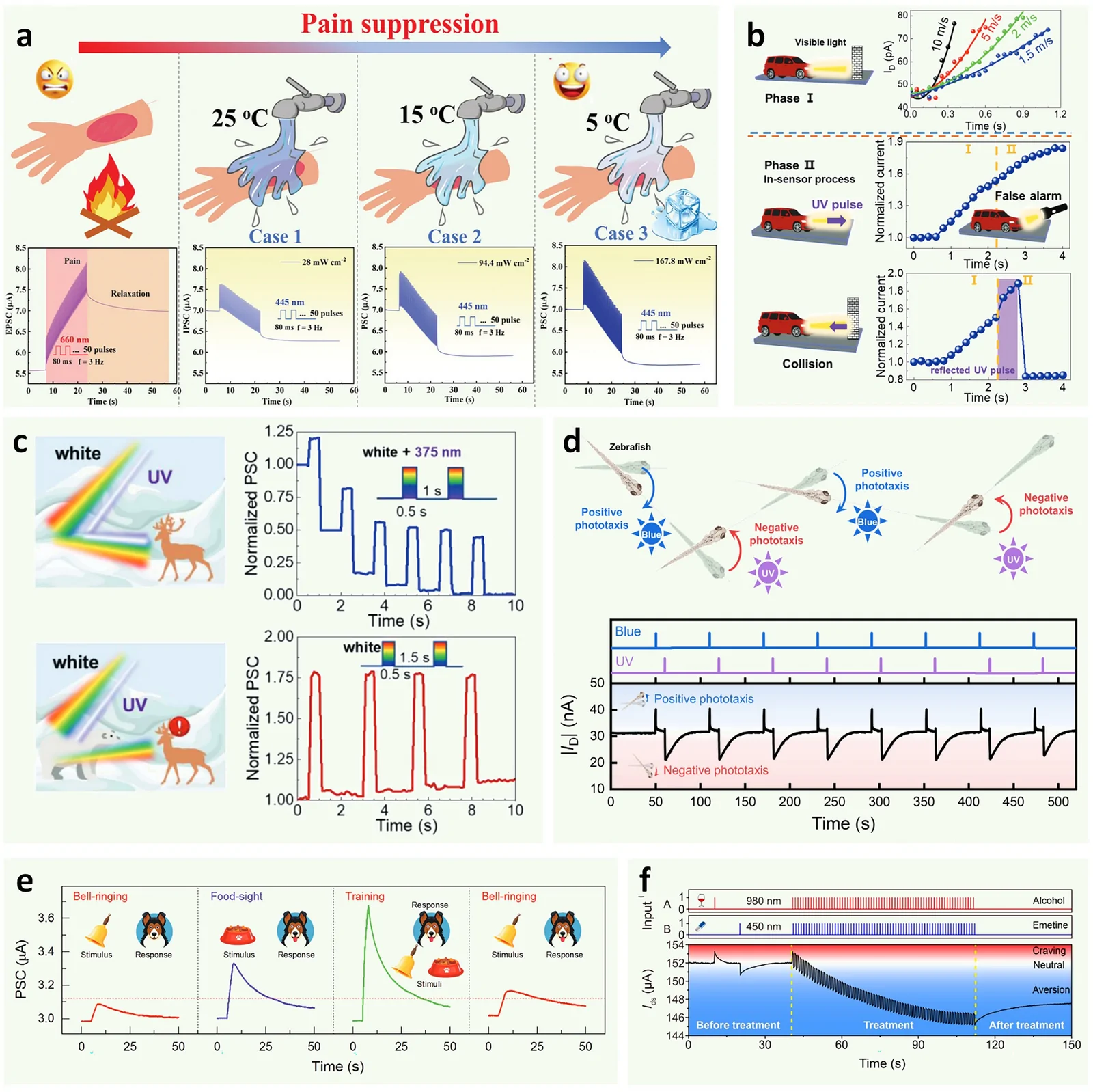
Application of AOC synaptic devices in scene simulation. **a** Simulation of pain perception and relief processes based on CuPc/P (VDF-TrFE) synapses Copyright 2022, Wiley–VCH GmbH [85]. **b** Collision detection using WSe_2_ optoelectronic synapse. **c** Simulation of alerting and foraging behavior of four colored reindeer based on WSe_2_ synapses. Copyright 2023, Wiley–VCH GmbH [133]. **d** All-optical synaptic response of a-Si:H/a-Ga_2_O_3_ phototransistor simulating zebrafish phototaxis. Copyright 2024, Tsinghua University Press [66]. **e** Classical condition simulation in Pavlov’s dog experiments. Copyright 2019, WILEY‐VCH Verlag GmbH & Co. KGaA, Weinheim [27]. **f** Aversion learning. Copyright 2021, American Chemical Society [24]

AOC synaptic devices have been successfully applied to simulate and simulate the basic functions of biological synapses, especially pain perception and relief processes. Ji et al*.* [85] fabricated an AOC synaptic device based on an organic semiconductor (CuPc) and ferroelectric polymer (P(VDF-TrFE)), which exhibited bidirectional responses at 660 and 445 nm wavelengths, successfully replicating essential synaptic functions like PPF and STP/LTP. This bidirectional response characteristic enabled the device to simulate the action of nociceptors, responsible for pain sensation and relief. For instance, touching a hot object induces an immediate sense of pain that gradually subsides over time, and exposure to cool water alleviates the pain further, with colder temperatures enhancing the effect. In this model, 660 and 445 nm light pulses correspond to thermal (excitatory) and cooling (inhibitory) signals, respectively. As depicted in Fig. 14a, when stimulated with a 660 nm pulse, the device generates sustained PPF, analogous to the release of excitatory neurotransmitters in biological synapses during thermal pain perception, thus mimicking the excitatory response in pain processing. Conversely, consecutive 445 nm pulses significantly suppress the PPF induced by previous 660 nm pulses, simulating pain relief upon exposure to cooling stimuli. The inhibitory effect on photocurrent is amplified with increased pulse intensities at 445 nm (e.g., 28, 94.4, and 167.8 mW cm^−2^), akin to pain relief experienced with progressively colder water (such as at 25, 15, and 5 °C). This bidirectional light modulation capability effectively simulates the dynamic responses involved in pain perception and relief, demonstrating promising potential for applications in bioelectronics.

In traditional collision detection systems, optical sensors typically operate within a single wavelength band (visible or infrared), leading to inevitable delays, data redundancy, and increased energy consumption. Wu et al*.* [133] have demonstrated that artificial synaptic devices based on WSe_2_ can respond to light of different colors (such as red, green, blue, and ultraviolet). This property enables WSe_2_ artificial four-color light sensors to perceive the position and distance of surrounding objects by detecting light of varying wavelengths, offering an effective solution for collision detection. As illustrated in Fig. 14b, when the WSe_2_ device detects an approaching object, an increase in light intensity results in significant current variations, indicating potential collision risks. Further analysis of wavelength and intensity changes allows precise calculation of object position, approach velocity, and potential collision time. However, relying solely on changes in photocurrent to determine collisions may be insufficient due to strong interferences such as intense reflection or variations in light sources, which could lead to false alarms. To address this issue, researchers introduced a two-stage logical algorithm into the system. In the first stage, the device monitors changes in photocurrent to assess potential collisions. In the second stage, if an increase in photocurrent occurs, the system emits ultraviolet light pulses for further confirmation. Only in the presence of a reflecting object does the UV pulse reflect, resulting in a significant current decrease, indicating an imminent collision. In this study, they also utilized WSe_2_-based synapses to mimic reindeer behavior in snow environments where they identify potential threats and food sources. This simulation process triggers physiological responses in reindeer under different lighting conditions through a combination of UV and white light stimuli. To simulate background light reflections in snowy environments, a mixed light pulse consisting of UV (0.3 mW cm^−2^) and white light (1.2 mW cm^−2^) was first applied to the WSe_2_ synaptic device. Such light pulses reproduce lighting conditions in snow environments devoid of predators or vegetation. As shown in Fig. 14c, under intermittent pulses, the PSC gradually decreases to near-zero values, indicating that UV light stimulation predominantly induces strong LTD, allowing the device to enter a relaxed state with minimal power consumption. This state simulates the low-power resting state of organisms in the absence of predator threats. To further simulate the presence of potential threats or food, subsequent stimulation with white light alone, without UV light input, triggers LTP in the synaptic device. This increased current consumption simulates the anxious response of organisms when detecting predators or the excitement upon discovering food. This process replicates the physiological responses of reindeer sensing potential threats or food in their environment. In summary, multi-wavelength responsive AOC synaptic devices not only effectively perceive changes in light sources but also reduce delays and improve energy efficiency through processing capabilities, offering new avenues for developing smarter environmental sensing devices.

Zebrafish, as a common model organism, is widely utilized in neurobiology research, particularly in behavioral and photoreception studies. Zebrafish exhibit phototactic behaviors in response to light of different wavelengths: They are attracted to visible light and avoid UV light. This behavior offers clues to understanding biological photoreception mechanisms. Yoon et al*.* [66] effectively replicated zebrafish phototaxis using a-Si:H/a-Ga_2_O_3_ phototransistors, successfully mimicking their physiological responses to different light wavelengths. In their experiments, researchers applied light pulses of 455 nm (blue light) and 245 nm (UV) separately (Fig. 14d). The 455 nm light pulse simulated the zebrafish's positive phototactic response, where the device generated EPSC indicating attraction to the light source. Conversely, the 245 nm light pulse simulated the negative phototactic response, generating IPSC indicating avoidance of UV light. Through this approach, a-Si:H/a-Ga_2_O_3_ synaptic device achieves precise responses to light stimuli and simulates organismal behaviors under different environmental conditions.

Associative learning is a process in which neural connections in the brain are formed through repeated stimulation within a short time frame. This form of learning encompasses behaviors such as classical conditioning, where distinct stimuli are paired repeatedly to establish a connection. A classic example of this is Pavlov's experiment with dogs, which highlights the fundamental principles of associative learning. In this experiment, food serves as an unconditioned stimulus that directly induces salivation in the dog, while the ringing of a bell, initially a neutral stimulus, does not evoke such a response. However, after repeated pairings of the bell with food, the dog begins to salivate merely at the sound of the bell, even in the absence of food. This phenomenon illustrates the formation of a conditioned reflex and the learning process, in which the association between food and bell is gradually established through training. In efforts to simulate this process, researchers have successfully recreated Pavlov’s classic experiment using neuromorphic devices. For instance, Ahamed et al*.* [27] employed BP-based synaptic devices to simulate this process under full optical control, as shown in Fig. 14e. They used light pulses of 280 nm and 660 nm wavelengths to represent food and bell stimuli, respectively. When only a 660 nm light pulse was applied, the resulting photocurrent did not exceed the threshold, simulating the lack of salivation. Conversely, the 280 nm light pulse, mimicking the food stimulus, generated a photocurrent above the threshold, simulating salivation. After repeated training, when both bell and food stimuli were applied together, the dog would salivate at the bell sound alone, illustrating the successful formation of the conditioned reflex. In the context of aversive learning, synaptic devices must also possess the ability to simultaneously modulate excitatory and inhibitory signals. In AOC synaptic devices, light pulses of varying wavelengths induce long-term potentiation or depression of postsynaptic currents, enabling the simulation of aversive learning. This form of learning is crucial in the treatment of addictive behaviors such as alcohol dependence. In the therapeutic process, patients combine alcohol consumption with emetic drugs to gradually enhance their aversion to alcohol. Following alcohol consumption, the brain enters an excitatory state, which is then followed by the induction of vomiting through the administration of emetic drugs. This pairing gradually increases the patient's aversion to alcohol. In the simulation [24], alternating positive and negative photoconductance in the synaptic devices mirrors the process of drinking and subsequent emetic response, effectively modeling aversive learning, as illustrated in Fig. 14f. Through the use of AOC synaptic devices, associative learning not only holds potential for simulating biological behaviors but also opens new avenues for simulating advanced learning and behavioral training within neuromorphic computing. These devices, capable of mimicking complex learning processes with low power consumption and high hardware efficiency, represent a promising frontier for advancing neuromorphic computing and artificial intelligence.

### Flexible Electronics

With the rapid development of wearable electronics, there is an increasing demand for flexible devices that combine ultralow power consumption, robust bending stability, and on-device data processing capability. Consequently, researchers have begun to explore the integration of AOC synaptic devices with flexible substrates to enable mechanically compliant AOC synapses, addressing emerging application requirements in wearable electronics and optical information processing systems.

**Fig. 15:**
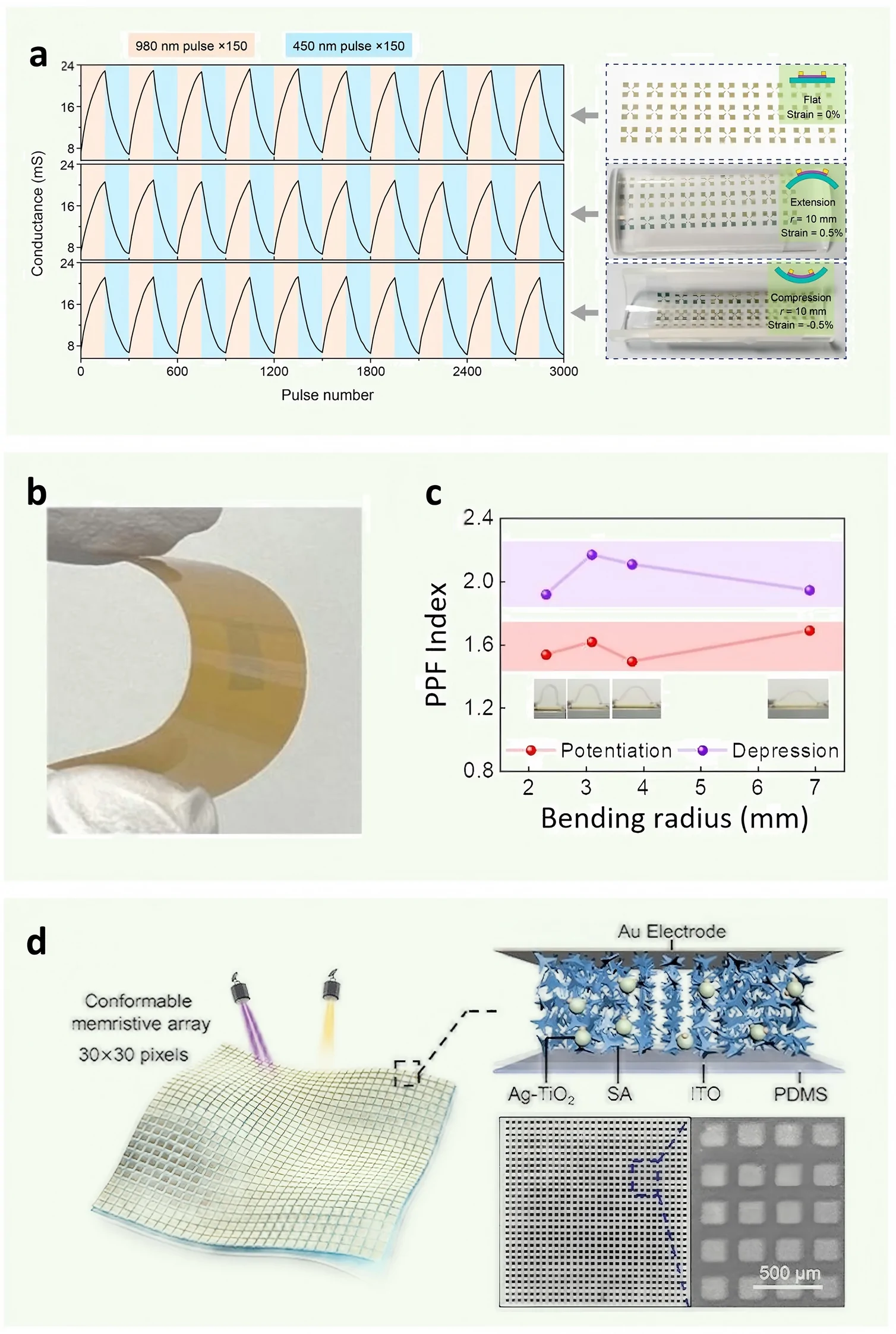
Application of AOC synaptic devices in flexible electronics. **a** Cyclic LTP/LTD curves of the device in the flat (top), bending (middle), and folding (bottom) states. The right panel shows the corresponding photographs of the device in the flat, bending, and folding states, respectively Copyright 2021, American Chemical Society [24]. **b** Retention of synaptic plasticity under consecutively applied potentiation/depression pulses. **c** PPF index as a function of banding radius. Published by Elsevier Ltd [223]. **d** Diagrams of the conformable optoelectronic memristor array and the device structure. Copyright 2024 The Author(s). Advanced Science published by Wiley–VCH GmbH [67]

Hou et al*.* [24] demonstrated a flexible AOC synaptic device based on a minimalist two-terminal architecture, in which graphene served as the conductive channel, while Pyr-GDY and PbS quantum dots functioned as charge trapping layers responsible for inhibitory and excitatory photoresponses, respectively. A wafer-scale heterojunction thin film with an area of 6 cm × 6 cm was fabricated and subsequently transferred onto a 0.1-mm-thick flexible PET substrate, enabling the construction of a 14 × 30 flexible synaptic array. When subjected to tensile or compressive strains below 1%, the PSC degradation remained below 7%, and the PPF index fluctuated by less than 5%. As shown in Fig. 15e, the device preserved stable LTP and LTD characteristics under flat, bent, and folded states, exhibiting reproducible cycling performance without noticeable degradation. Han et al*.* [223] employed CVD to synthesize wafer-scale two-dimensional PtSe_2_ films exhibiting a unique wavelength-dependent photoconductive response. PPC was observed under long-wavelength illumination (625–940 nm), whereas NPC emerged under short-wavelength excitation (405 nm). Using a PMMA-assisted transfer process, the PtSe_2_ film was nondestructively integrated onto a flexible PI substrate. As shown in Fig. 15b, the resulting flexible AOC synaptic device demonstrated excellent mechanical compliance. Moreover, as illustrated in Fig. 15c, the PPF index showed negligible variation under both mild bending (radius = 6.9 mm) and severe bending (radius = 2.3 mm). The PtSe_2_ layer retained complete optical synaptic functionality after flexible integration. Such low-dimensional materials endow AOC synapses with outstanding mechanical flexibility, allowing them to withstand repeated deformations in wearable scenarios, while simultaneously enabling integrated optical sensing, memory, and processing for real-time image detection, storage, and recognition with reduced energy consumption and latency.

In addition, organic materials, owing to their intrinsic flexibility and uniform film-forming capability, are particularly well suited for flexible AOC synaptic devices. Shan et al*.* [67] reported a flexible plasmonic optoelectronic memristor array based on Ag-TiO_2_ nanoclusters embedded in a SA film. The naturally derived polymer SA provides high mechanical flexibility and excellent film uniformity, enabling homogeneous encapsulation of Ag-TiO_2_ nanoclusters while maintaining structural integrity under bending. Using PDMS as the substrate, a mechanically compliant 30 × 30 pixel array was realized, as shown in Fig. 15d. The PDMS-based memristor array could bend naturally under gravity and be conformally transferred onto a three-dimensional artificial eyeball surface without wrinkles or air bubbles, demonstrating excellent deformation adaptability and laying the foundation for hemispherical device architectures. The efficient integration of flexible substrates with functional layers provides a solid hardware basis for wearable vision systems and biomimetic robotic vision.

## Challenges

AOC synaptic devices, as a burgeoning component in neuromorphic computing, exhibit tremendous potential through their bidirectional regulation of synaptic behavior solely via optical signals [224–226]. These devices significantly reduce energy consumption, offer ultra-fast signal processing, and enable unique advantages in non-contact communication. These attributes position them as highly effective in advanced neural network computation, intelligent visual systems, and biomedical sensing applications. Recent advancements in the research surrounding AOC synaptic devices have been notable across mechanisms, material synthesis, structural design, and practical applications. Despite their considerable benefits, the development of AOC synaptic devices faces numerous challenges.

The foremost among these challenges is the stable realization of PPC and NPC all-optical modulation behaviors, which are crucial to the operation of AOC synaptic devices. The operational mechanics of AOC involve intricate processes such as charge transfer, defect trapping, and polarization control. These processes exhibit significant variability across different material systems, complicating the development of targeted solutions. This underscores the dependency of device mechanisms and performance largely on the intrinsic properties of the active/functional materials employed. Unfortunately, current materials often fail to meet simultaneously the demands of low energy consumption, high photoresponse efficiency, and long-term stability [193]. For example, most oxide semiconductors have wide bandgaps, which restrict the spectral range of photoresponse; low-dimensional materials and perovskites still lack sufficient long-term stability and environmental robustness; organic materials often have low carrier mobility. Additionally, most materials are susceptible to degradation and defect accumulation under prolonged illumination, particularly under high-intensity or continuous light.

Furthermore, the performance of AOC synaptic devices is also highly sensitive to environmental factors, including gas composition, temperature, humidity, and prolonged light exposure. Variations in ambient conditions can significantly affect charge trapping dynamics, carrier transport, and optical absorption properties, leading to instability in synaptic weight modulation. Photothermal effects and energy consumption issues are inevitable considerations in the commercial development of AOC synaptic devices [24]. While optical signals enable non-contact and ultra-fast modulation, frequent optical stimulation leads to increased device temperatures, which can negatively impact material stability and device longevity, a critical hurdle for future low-power neuromorphic computing. From an engineering perspective, manufacturability and yield pose additional challenges. The multilayer structures and stringent process control required for AOC devices often result in low fabrication reproducibility and limited yield, especially when scaling toward wafer-level production.

The complexity of the fabrication process and the difficulty of integration have also affected the large-scale application of AOC synaptic devices. The multilayer fabrication processes of photosensitive materials, semiconductors, and insulating layers often result in device inconsistency and repeatability, demanding exceedingly high manufacturing precision. While individual devices may achieve satisfactory light response, challenges in signal consistency and response speeds in complex scenarios are still prevalent in large-scale integrated circuits, particularly for complex visual perception tasks.

CMOS compatibility is another major obstacle hindering the translation of AOC synaptic devices from laboratory demonstrations to industrial applications. Many photosensitive materials used in AOC devices require complex fabrication processes, unconventional materials, or high-temperature treatments that are incompatible with standard CMOS back-end-of-line (BEOL) processes. Additionally, integrating optical components with electronic circuits within a CMOS platform poses challenges in terms of process alignment, thermal budgets, and system complexity. Without ensuring CMOS-compatible materials, fabrication steps, and operating conditions, the large-scale commercialization and system-level integration of AOC synaptic devices remain difficult.

Beyond material- and device-level limitations, the scalability of AOC synaptic devices remains a critical challenge for practical neuromorphic systems. While many studies demonstrate promising synaptic behaviors at the single-device level, scaling these devices into large crossbar arrays or high-density integrated systems often leads to pronounced performance degradation. Issues such as device-to-device variability, non-uniform optical response, and cumulative photothermal effects become increasingly severe as array size increases. Moreover, optical addressing in large-scale architectures introduces additional challenges related to light delivery efficiency, optical crosstalk, and precise spatial control, all of which can adversely affect system-level reliability and learning accuracy. These scalability constraints currently limit the deployment of AOC synaptic devices in large-scale neuromorphic hardware.

It is worth noting that, despite the rapid progress of AOC synaptic devices, uniform performance evaluation standards and benchmarking protocols are still lacking. In particular, the absence of widely accepted quantitative metrics for stability, scalability, and system-level performance makes it difficult to directly compare different materials, device architectures, and operating mechanisms. Establishing standardized evaluation criteria will be essential for accelerating technology maturation and facilitating broader adoption in neuromorphic computing systems.

To overcome these challenges, future research should focus on and break through the following areas in Sect. 9 to further advance the development of neuromorphic computing and intelligent optical computing.

## Future Prospects

### Stability Improvement

Material stability represents one of the key factors limiting the further development of AOC synaptic devices. In particular, AOC devices that rely on gas adsorption and desorption mechanisms to achieve optical modulation of synaptic behaviors exhibit a high sensitivity to ambient environmental conditions, and their long-term reliability and controllability remain to be improved. Variations in gas composition, temperature, and humidity can induce fluctuations in device responses, leading to performance drift and degraded reproducibility. In addition, surface defect states and adsorption states may result in delayed response times or inconsistent device behaviors, thereby constraining the practical application potential of such devices. To address these challenges, future research should focus on systematically enhancing the environmental stability and operational reliability of AOC synaptic devices from both material and device perspectives. Effective strategies may include the introduction of surface passivation or protective coatings, optimization and modification of material systems, and appropriate device encapsulation. Ideally, protective coatings should maintain high optical transparency while effectively suppressing gas adsorption and mitigating environmental disturbances, thereby ensuring stable and reproducible device performance and laying a solid foundation for the large-scale practical deployment of AOC synaptic devices.

### Device Design

In AOC devices, device and structural design are crucial for optimizing optical signal transmission, conversion, carrier dynamics, and energy efficiency. AOC devices are typically divided into two main types: planar/vertical two-terminal memristors and three-terminal transistors [227]. Two-terminal devices offer simplicity and are conducive to large-scale integration, making them well suited for cost-effective production, while three-terminal devices exhibit greater control flexibility, which is particularly advantageous for multi-gate applications in complex neural networks. Thus, the choice of device type should be dictated by specific application scenarios to fully leverage the respective advantages of two-terminal and three-terminal devices. Second, low-power design is the key to device optimization, and energy consumption can be effectively reduced by shrinking the device size and optical pulse width. Furthermore, the use of flexible polymer substrates, such as PDMS or PET, imparts AOC devices with significant mechanical flexibility [228, 229], rendering them suitable for wearable electronics and smart electronic products.

### Application Exploration

AOC synaptic devices have demonstrated great potential for innovation in a variety of cutting-edge application scenarios, such as image recognition, logic operations, motion detection, visual perception and special scenario simulation [51, 84]. Future development should concentrate on pioneering new application paradigms and unique technological combinations to drive the evolution of intelligent computing and sensing systems. In intelligent visual and perception systems, AOC synaptic devices can be employed for dynamic light field reconstruction, enabling refocusing and viewpoint switching by modulating light field parameters. Such capabilities could support challenging tasks like autonomous robotic navigation and real-time scene interpretation, paving the way for ultra-high-resolution all-optical visual chips. Leveraging the wide spectral response of AOC devices, multi-channel spectral response systems can be developed to capture and interpret a broader range of spectral information, which is particularly beneficial for fine spectral analysis in applications like agriculture and environmental monitoring. Furthermore, the flexibility of AOC devices opens new avenues for the development of advanced smart wearable devices. By integrating with virtual reality (VR) and augmented reality (AR) systems, AOC devices can be harnessed to create all-optical control interfaces for human–computer interaction, enhancing the immersive experience and the naturalness of interaction.

### Large-Scale Integration

Current research predominantly focuses on employing individual devices to simulate basic synaptic plasticity, while the replication of complex human visual memory behaviors remains largely unexplored. The development of AOC synaptic arrays and their functional integration is a key driver for advancing large-scale neuromorphic systems [24, 31]. Constructing AOC synaptic arrays that exhibit complex behaviors requires several critical strategies. First, it is essential to select appropriate materials that can provide high optical responsiveness, bidirectional modulation capability, stability, and scalability under continuous light stimulation. Second, an important aspect of constructing functional arrays lies in effectively managing light and achieving multiplexing. Given that each synaptic device requires precise optical stimulation, utilizing waveguides to direct light to specific devices presents a promising approach. By integrating waveguide and optical multiplexing technologies, it becomes possible to utilize different wavelengths simultaneously, enabling complex synaptic training and functional diversity without the need for individual light sources for each synapse. Finally, the manufacturing of large-scale arrays necessitates scalable techniques. It is crucial to explore and optimize fabrication processes to ensure the consistency of thousands of synaptic devices, thereby facilitating their integration into comprehensive neuromorphic systems.

### Algorithm-Device Co-Development

The integration of neural network algorithms with AOC synaptic devices still faces several prominent challenges, including limitations in temporal synchronization, device-to-device variability in large-scale arrays, and the complexity of associated optical-electronic circuitry. Therefore, the development of a unified AOC-based hardware platform capable of supporting hybrid neural network architectures will be essential for enabling multidimensional perception and processing in complex environments. Future research should emphasize algorithm-driven device design, wherein the material and operational characteristics of AOC synaptic devices are customized to meet the requirements of specific algorithms. For instance, oxide-based materials, which can exhibit multi-level synaptic weight states, offer promising opportunities for advancing ANN-related implementations. Strengthening multi-modal data-fusion capabilities will also be critical. AOC device arrays have the potential to perform in situ fusion of optical, thermal, acoustic, and other sensory modalities, thereby enhancing system adaptability in real-world scenarios. In parallel, specialized optimization for edge intelligent applications is needed. For lightweight algorithms such as KNN, simplifying the optical correlation modules within AOC arrays and developing low-complexity, low-latency AOC-based edge-computing chips will greatly expand their deployment in IoT devices and energy-constrained environments. Together, these directions point toward a future in which tightly coupled algorithm-device co-design enables efficient, scalable, and adaptive neuromorphic systems.

### Standardized Evaluation System

As neuromorphic computing and synaptic devices continue to evolve, the demand for standardized evaluation systems has become increasingly critical. A standardized framework is essential for the ongoing assessment of AOC synaptic devices and neuromorphic systems in terms of performance, reliability, and scalability. Such systems ensure that research advancements can be directly compared, promoting reproducibility and aiding the acceleration of practical large-scale neuromorphic technologies. To establish a credible evaluation system, collaboration among researchers is necessary to consolidate a vast array of materials and systems, thereby constructing reliable reference frameworks. Moreover, interdisciplinary cooperation is vital for developing a universal evaluation framework, as AOC synaptic devices span multiple fields, including materials science, optics, electronics, biology, and neuroscience. Through collective efforts and knowledge sharing, the standardization of AOC synaptic technologies can be expedited, facilitating their transition to practical applications and large-scale deployment. This review aims to provide direction and insights for the construction of such standardized systems, assisting researchers in developing AOC synaptic devices that are both practical and scalable in their future work.

**Fig. 16:**
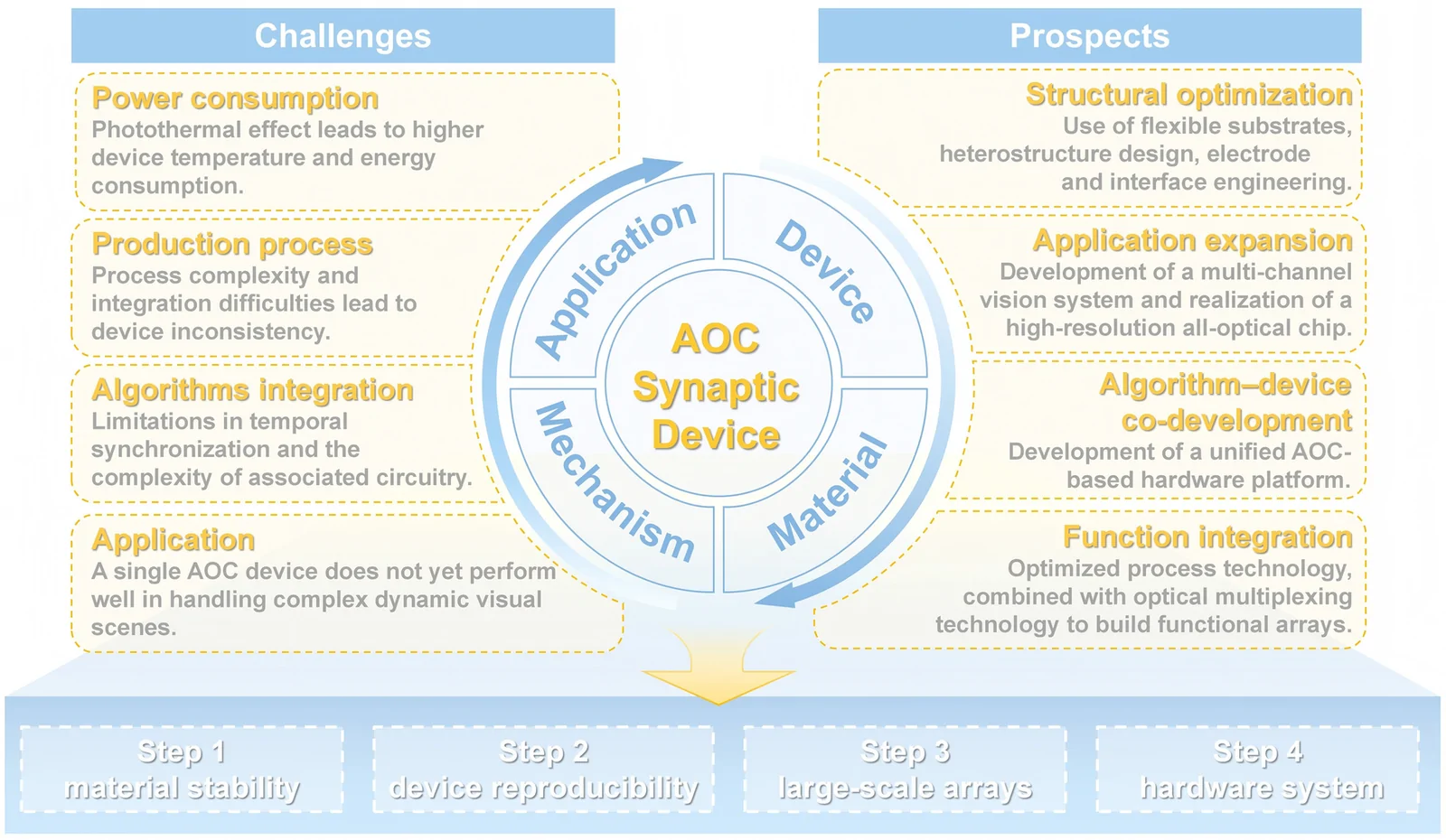
Challenges and prospects of AOC synaptic devices

In summary, although AOC synaptic devices have experienced rapid progress in recent years and demonstrated significant advantages in terms of energy efficiency, response speed, and functional integration, numerous critical challenges must still be addressed before their large-scale deployment in practical neuromorphic systems. The unresolved key bottlenecks include the long-term operational stability of devices under repeated optical stimulation, device-to-device variability in large-scale arrays, compatibility with CMOS fabrication processes, and sensitivity to environmental factors such as temperature and humidity. In addition, the lack of standardized performance metrics and benchmarking protocols further hampers objective comparisons among different material systems and device architectures. In response to these challenges, this chapter outlines the future development directions and potential improvement strategies for AOC synaptic devices. From a practical perspective, near-term research efforts should focus on enhancing material stability and device reproducibility while maintaining ultra-low-power operation. A key mid-term objective is the realization of CMOS-compatible AOC synaptic arrays with uniform optical responses and scalable manufacturing processes. In the long term, the co-design of materials, device architectures, and neuromorphic algorithms, together with the establishment of unified evaluation standards, will be crucial for translating AOC synaptic devices from laboratory demonstrations into reliable, application-ready neuromorphic hardware. The challenges and prospects for AOC synaptic devices are summarized in Fig. 16.

## Conclusion

AOC synaptic devices, as a promising neuromorphic computing paradigm, completely eliminate the problem of separation between sensing and processing by integrating computing functions into sensors. The advancement of AOC synaptic devices offers a transformative pathway to overcome the limitations of traditional computing architectures. Their potential in future neuromorphic computing is evident, offering the ability of both optical writing and optical erasing of information, as well as ultra-fast processing and low-power operation. However, the scalable development of high-performance AOC synaptic devices for neuromorphic computing remains challenging and requires a systematic exploration of their underlying physical mechanisms and design principles. In this review, we discuss the physical mechanisms and material applications of AOC synaptic devices, emphasizing the groundbreaking progress and application potential of these devices in neuromorphic computing. Specifically, we present an underlying framework for replicable and scalable design of AOC synaptic devices, which is expected to play a key role in advancing the development of neuromorphic computing. We believe that this framework provides universally applicable guidelines for the next-generation of AOC synaptic devices, serving as a fundamental hardware basis of neuromorphic computing for artificial intelligence.
